# Identifying locations susceptible to micro-anatomical reentry using a spatial network representation of atrial fibre maps

**DOI:** 10.1371/journal.pone.0267166

**Published:** 2022-06-23

**Authors:** Max Falkenberg, James A. Coleman, Sam Dobson, David J. Hickey, Louie Terrill, Alberto Ciacci, Belvin Thomas, Arunashis Sau, Fu Siong Ng, Jichao Zhao, Nicholas S. Peters, Kim Christensen

**Affiliations:** 1 Centre for Complexity Science, Imperial College London, London, United Kingdom; 2 Department of Physics, Imperial College London, London, United Kingdom; 3 ElectroCardioMaths Programme, Imperial Centre for Cardiac Engineering, National Heart & Lung Institute, Imperial College London, London, United Kingdom; 4 Auckland Bioengineering Institute, The University of Auckland, Auckland, New Zealand; University of Minnesota, UNITED STATES

## Abstract

Micro-anatomical reentry has been identified as a potential driver of atrial fibrillation (AF). In this paper, we introduce a novel computational method which aims to identify which atrial regions are most susceptible to micro-reentry. The approach, which considers the structural basis for micro-reentry only, is based on the premise that the accumulation of electrically insulating interstitial fibrosis can be modelled by simulating percolation-like phenomena on spatial networks. Our results suggest that at high coupling, where micro-reentry is rare, the micro-reentrant substrate is highly clustered in areas where the atrial walls are thin and have convex wall morphology, likely facilitating localised treatment via ablation. However, as transverse connections between fibres are removed, mimicking the accumulation of interstitial fibrosis, the substrate becomes less spatially clustered, and the bias to forming in thin, convex regions of the atria is reduced, possibly restricting the efficacy of localised ablation. Comparing our algorithm on image-based models with and without atrial fibre structure, we find that strong longitudinal fibre coupling can suppress the micro-reentrant substrate, whereas regions with disordered fibre orientations have an enhanced risk of micro-reentry. With further development, these methods may be useful for modelling the temporal development of the fibrotic substrate on an individualised basis.

## 1 Introduction

Atrial fibrillation (AF) is the most common cardiac arrhythmia with significant impacts on both morbidity and mortality [1]. Despite extensive research, there are significant disagreements within the cardiac electrophysiology community as to the mechanisms underlying AF [2, 3].

Most studies into the mechanistic origin of AF focus on the maintenance of AF, typically arguing for either organised (mother waves or stable rotors) or disorganised mechanisms (multiple reentrant wavelets). However, if both organised and disorganised mechanisms of AF coexist on a continuous spectrum of electromechanical organisation, as suggested recently [4], it is unlikely that any single treatment strategy will be successful across the full spectrum of AF mechanisms. It is for this reason that personalised, patient-specific approaches to AF treatment have become a key research focus in recent years [5, 6].

Here we computationally investigate one mechanism of AF initiation and maintenance: the formation of micro-anatomical reentrant circuits, which are continuously activated electrical circuits anchored to the fibre structure of the atria [7]. This mechanism is controversial, with supporting clinical evidence still required, but it has some “potential to unify … previous discrepant observations” in the AF literature [8]. Importantly, the size of experimentally identified micro-reentrant circuits is often at, or below, the spatial resolution which can be resolved with conventional multi-electrode mapping [7, 9]. Similarly, the interstitial fibrosis which insulates these circuits is often not easy to detect using conventional LGE-MRI [4], and extracting precise fibrosis densities is challenging given the variability in signal thresholding choices between scans and patients [10]. Hence, computational approaches allow for a degree of hypothesis testing which avoids some of these challenges.

In this proof of concept, we introduce a novel method to assess the feasibility of patient-specific predictions for the distribution of micro-anatomical reentry across the atria. In particular, the method is inspired by the idea that it may be possible to predict the emergence of micro-reentry before the atria have accumulated sufficient interstitial fibrosis, in contrast to most other approaches which take a static view of the AF substrate [5, 11].

Starting from image-based models of the atria, we combine data regarding the atrial geometry and the underlying myocardial fibre structure to form a spatial network. By progressively removing connections in the atrial structure, we assess where in the network a path exists which is sufficiently long to harbour micro-reentry, an approach related to the study of percolation in network science [12]. In the current work, our primary goal is to understand the utility of the method and demonstrate its future potential. With this in mind, our research focuses on how the regions predicted by our method of being susceptible to micro-reentry depend on fibre structure and atrial geometry.

## 2 Aims

Experimental evidence from explanted human hearts has suggested that “AF may be driven by microanatomic reentrant AF drivers anchored to fibrotically insulated tracks within the complex atrial wall” [9]. To ensure that a given fibre tract can sustain micro-reentry, the reentrant pathway through a fibrotically insulated fibre tract must be at least one refractory wavelength long.

**Fig 1:**
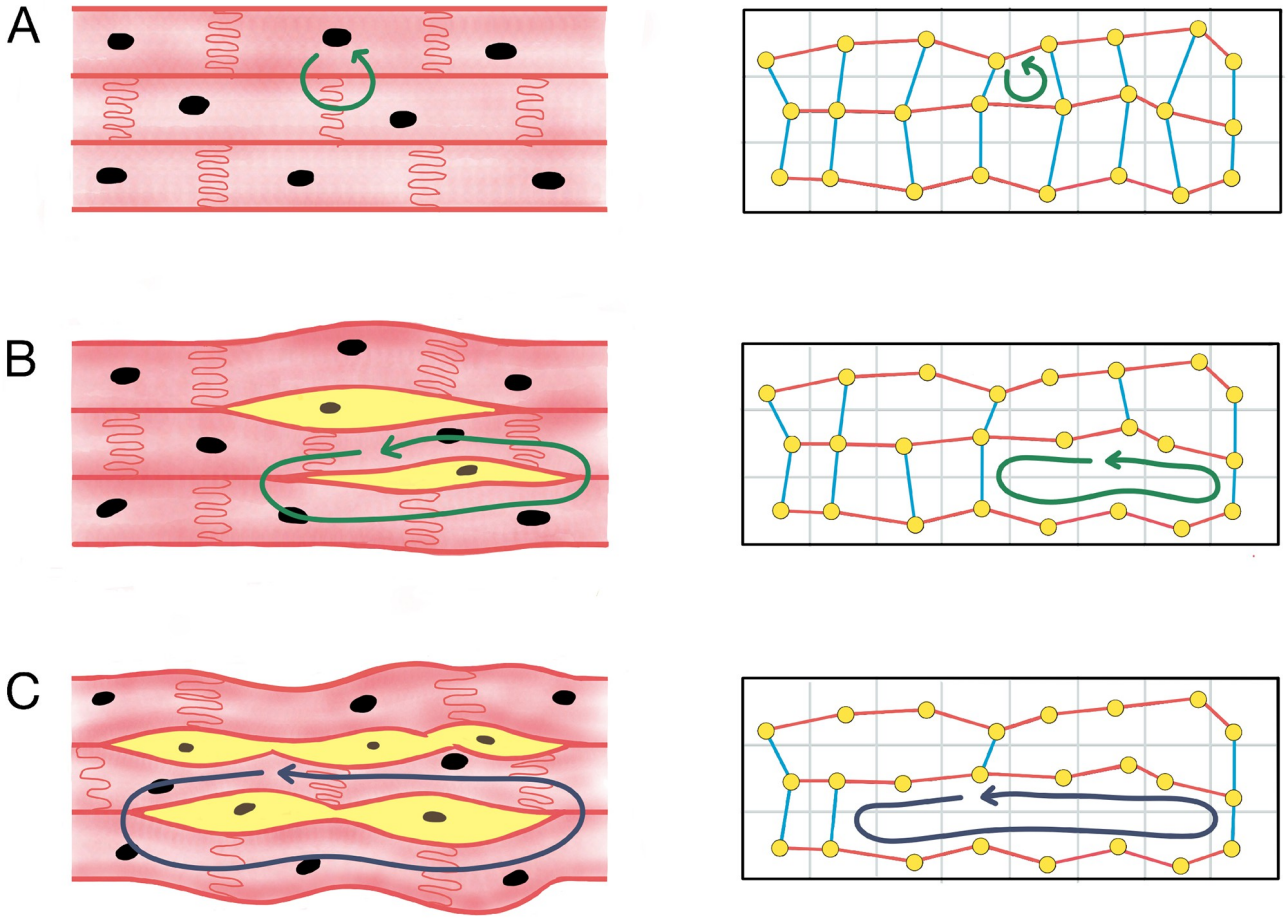
A schematic illustrating our approach. Left column: (A) In the healthy atrial myocardium, cardiomyocytes (pink cells) are well coupled along and across the principle fibre directions, such that the longest reentrant loop is too short to sustain micro-anatomical reentry (green arrows). (B) Over time, the atria accumulate interstitial fibrosis (yellow cells) forming short segments of electrically isolated fibre. (C) The length of these segments grows as more interstitial fibrosis accumulates until the isolated segments are sufficiently long to harbour micro-reentry (blue arrows). We hypothesise that the accumulation of interstitial fibrosis can be modelled as a spatial network. Right column: Nodes (yellow points) representing a group of cardiomyocytes are connected to their neighbours along fibres (red links) and across fibres (blue links). If the density of interstitial fibrosis is low, there are many transverse (blue) links. We model the increase in the density of interstitial fibrosis as the progressive removal of transverse links, and equate the micro-anatomical reentrant substrate to regions of the spatial network where a loop exists that is longer than one refractory wavelength.

Our approach is illustrated schematically in Fig 1. Denoting the minimum refractory wavelength during fibrillation as *τ*, our aim is to assess where in the atrial structure fibrotically insulated reentrant pathways exist of length *ℓ*, such that *ℓ* ≥ *τ*. In a healthy heart with minimal interstitial fibrosis, atrial myofibres are well coupled in both the longitudinal and transverse directions. Hence, the longest possible reentrant path is significantly shorter than one refractory wavelength, *ℓ* ≪ *τ*. As the density of electrically insulating interstitial fibrosis increases, the longest reentrant pathway increases until a threshold density is reached at which *ℓ* ≥ *τ* and the fibrotically insulated fibre tract can sustain continuous reentry during AF. Importantly however, this threshold density may vary across different regions of the atria; some regions may be susceptible to micro-reentry at a low fibrosis density, whereas other regions may require a significantly higher fibrosis density before micro-reentry can be induced. If the density of fibrosis is too high in a region, micro-reentry may be prevented due to the absence of any closed loops (compact fibrosis).

Our hypothesis is that the process of electrically insulating fibre tracts with interstitial fibrosis can be modelled using spatial networks. A spatial network is a graph consisting of nodes representing entities in space, and edges representing spatial connections between those entities [13]. In our case, a node represents one or more atrial cardiomyocytes in a specific atrial region, and an edge between two nodes indicates whether those groups of myocytes are electrically coupled, that is whether an activation wavefront can pass directly from the group of myocytes represented by one node, to the group of myocytes at the other node.

Starting from a network in which the local density of edges is high, our assertion is that the accumulation of insulating interstitial fibrosis in the atria is structurally equivalent to the progressive removal of edges in our spatial network. If the density of fibrosis is low, then the density of edges in the spatial network is high, and similarly, if the density of fibrosis is high, the density of edges in the spatial network is low. Then, by identifying loops in the spatial network of length *ℓ* ≥ *τ*, we can identify which atrial regions may act as a substrate for micro-anatomical reentry at a given fibrosis density.

## 3 Materials and methods

**Fig 2:**
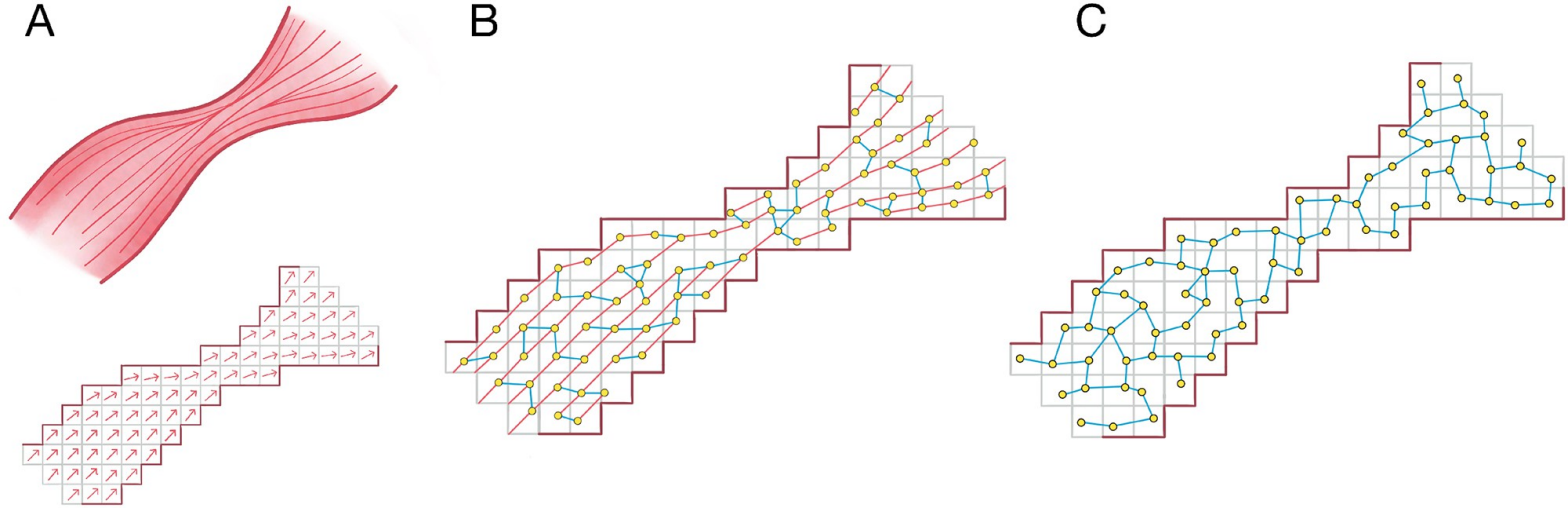
A schematic showing the construction of spatial networks from atrial imaging data. (A) An image based model of the atria is derived from atrial imaging. The model, represented by a mesh of voxels, is coupled with vectors representing the underlying fibre orientation at each point. (B) In the fibre model, a spatial network is generated where nodes (yellow points) are connected with probability 1 along fibre tracts (pink edges), and low probability across fibre tracts (blue edges). (C) In the fibre-less null model, the probability of forming an edge is not biased by the underlying fibre orientation.

In this paper, we generate spatial networks from three atrial datasets (see below), each consisting of a voxel mesh with local fibre orientation vectors. For each dataset, we generate spatial networks according to two different approaches: one which preserves atrial fibre orientations in the spatial network (the fibre model), and a null model approach which ignores the fibre orientation data but retains other structural information such as wall thickness (the fibre-less null model). These two approaches are shown schematically in Fig 2. By utilising both approaches, we are able to identify how the underlying atrial fibre structure affects the observed micro-reentrant substrate.

### 3.1 Atrial datasets

The spatial network algorithm is tested on three atrial datasets, one human dataset with MRI derived geometry, but synthetic fibre orientations, and two sheep atrial datasets where the fibre structure has been inferred from high resolution serial surface imaging. Each dataset provides a 3d grid of vectors indicating the local fibre orientation at each point in the atria. The sheep datasets are high resolution and inferred directly from the anatomy of each dataset. Summary statistics for the three image-based atrial fibre maps are given in Table 1.

**Table 1:** Summary information for the three atrial fibre orientation datasets.

	Human	Sheep Healthy	Sheep Heart failure
**Imaging Method**	MRI	Serial Surface Imaging	Serial Surface Imaging
**Original Image Resolution**	330*μm*	50*μm*	25*μm*
**Fibre Orientation Extraction**	Estimated	Anatomically derived	Anatomically derived
**Fibre Orientation Method**	Semi-automated rule based approach focusing on key bundles	Eigen-analysis of structure tensor	Eigen-analysis of structure tensor
**Resolution after Coarse Graining**	330*μm*	300 *μm*	300*μm*
**Dataset Reference**	[14, 15]	[16]	[17]

#### 3.1.1 Synthetic atrial fibre map

The human dataset is detailed in [15] based on techniques developed in [14]. The atrial geometry used provides no information as to the atrial fibre structure. Therefore, a synthetic fibre map is constructed using a semi-automated rules-based approach which is described in the supplementary material (SM) section 1.1.1.

#### 3.1.2 Anatomically derived fibre maps

We study two anatomically derived sheep atrial datasets: one healthy sheep acquired in [16] and a sheep with pacing-induced heart failure [17]. These datasets are individual-specific, retaining significant local heterogeneity in the fibre maps, and are provided at high resolution, in contrast to the synthetic human fibre map. Information regarding the data acquisition process is provided in SM section 1.1.2. In this proof of concept, our aim is not to assess the differences in micro-anatomical reentry between humans and sheep, or between healthy and heart failure (HF) sheep, but to understand how different sources of fibre orientation data affect our results.

### 3.2 Constructing spatial networks with fibre structure

**Fig 3:**
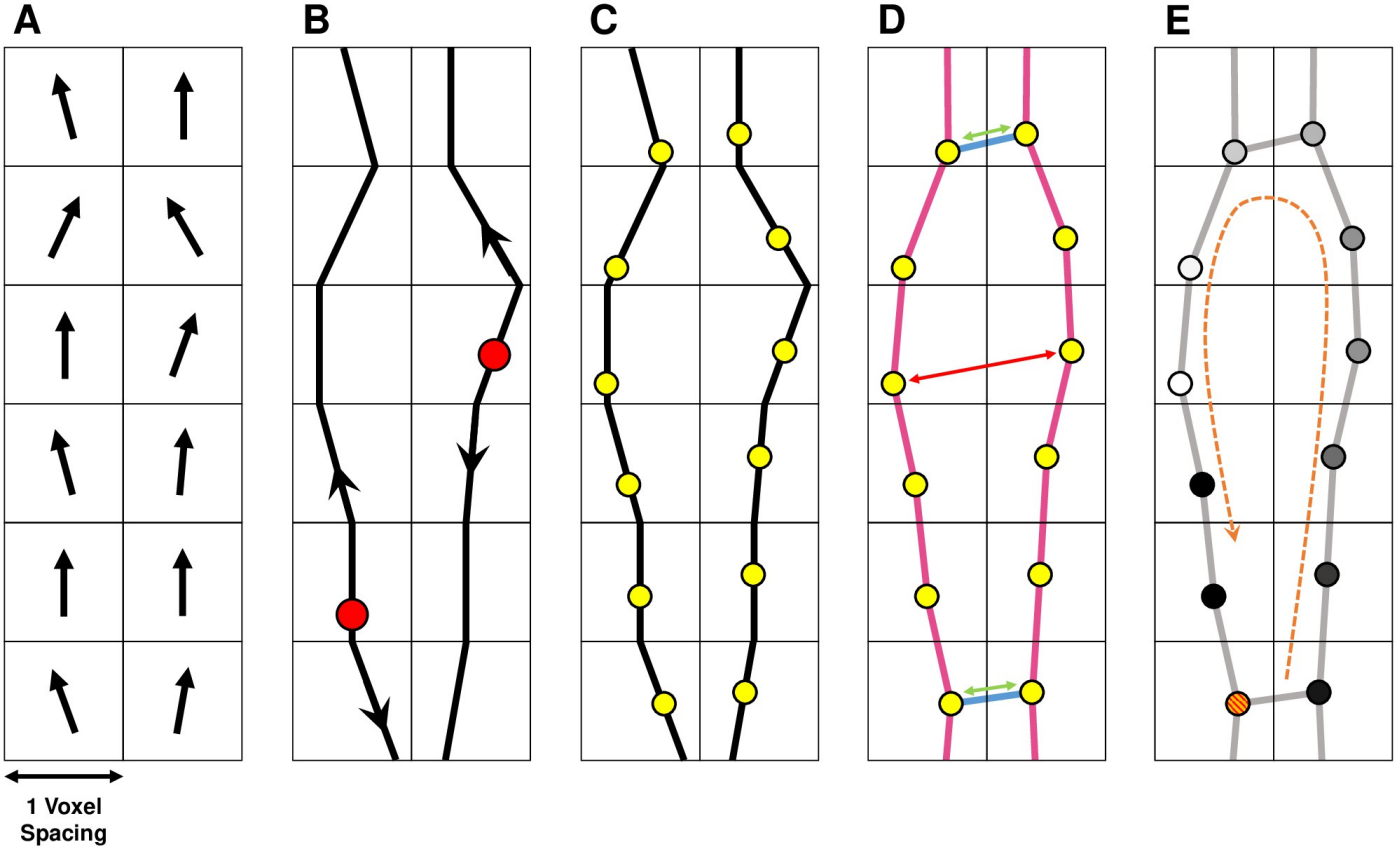
A 2D schematic showing the conversion of local fibre orientation vectors into global fibre tracts which are seeded with nodes and coupled into a spatial network. (A) Each image-based model provides a vector field for the local fibre orientation. (B) Beginning from a set of random seed points (red circles), fibres are generated using tractography, propagating forward and backwards in the direction of the local orientation vector. (C) Nodes (yellow circles) are placed at approximately equal intervals along the fibres. (D) Nodes are coupled to neighbouring nodes with probability 1 if nodes are adjacent and in the same fibre (pink edges), and according to a monotonically decreasing function of distance, *p*(*x*;*r*, *c*), otherwise (blue edges). Nodes which are close together (green arrows) are more likely to be connected than those which are far apart (red arrows). (E) By applying a discrete diffusion model (see section 3.4) to the network, we identify the micro-reentrant substrate by observing where conduction block (red/orange circle) results in a wavefront (white circle) reentering a blocked fibre.

The schematic shown in Fig 3 outlines the process by which each image-based model is converted into a spatial network in which the underlying fibre structure is retained.

#### 3.2.1 Fibre tractography

Fibre tracts are generated across the atrial geometry by applying a modified version of the Evenly Spaced Streamlines (ESS) algorithm, see [18]: (1) Local fibre orientation data for a given atrial dataset is coarse grained to a specific voxel resolution, Fig 3(A). (2) Placing seed points randomly in the atrial mesh, separated by at least *d*_*sep*_ = 0.7, global fibre tracts are constructed by propagating forwards and backwards along the local fibre orientation vectors until terminated according to a set of pre-specified rules, Fig 3(B). (3) After fibre tracts have been generated to cover the atrial mesh at an approximately fixed density of 1 node per voxel, points are placed evenly along each fibre, Fig 3(C). The ESS algorithm was modified to maximise fibre length by generating longer fibres first. Importantly, unlike other common tractography methods, this modified ESS approach ensures that the density of fibres is approximately uniform across the atrial structure. Technical details for the tractography process are provided in SM section 1.2.

#### 3.2.2 Generation of spatial network structure

The fibres generated from tractography are not connected to each other, Fig 3(C). To connect fibres in a single spatial network, nodes are placed at even intervals (steps of 1 voxel length) along fibres starting at a random seed point. These nodes connect to neighbouring nodes in the same fibre with probability *p* = 1. Any two nodes which do not lie on the same fibre are connected with probability

$$\begin{array}{c} p\left(x;r,c\right)=\frac{1}{e^{r(x-c)}+1}, \end{array}$$ (1)

where *x* is the distance between the nodes, *r* = 7 is an arbitrary steepness parameter, and *c* is a characteristic distance. Connecting nodes according to a distance dependent attachment function is a standard technique when studying percolation on spatial networks [12]. For better performance, only nodes separated by *x* < 2 are considered for connection. By varying the spatial coupling parameter *c*, the number of transverse connections between fibres can be controlled from a state where fibres are strongly connected at *c* ≈ 1, to a state where fibres become increasingly disconnected as *c* → 0. We can think of the coupling *c* as representing the grade of fibrosis. If the coupling is large, there is no, or only low-grade, interstitial fibrosis. As coupling is reduced, this is equivalent to a greater burden of interstitial fibrosis. Ideally, our distribution of spatial coupling should be modulated using individual-specific fibrosis data, however, this information is not available with the current datasets.

For each dataset, spatial networks are generated with five values of the characteristic distance, *c*. The specific values used and corresponding risk parameters (see section 3.5.1), are given in S2 Table in SM section 1.3.1 in S1 File. For convenience, specific parameter choices are labelled as having a low, medium or high global risk of micro-reentry.

### 3.3 Constructing spatial networks without fibre structure

**Fig 4:**
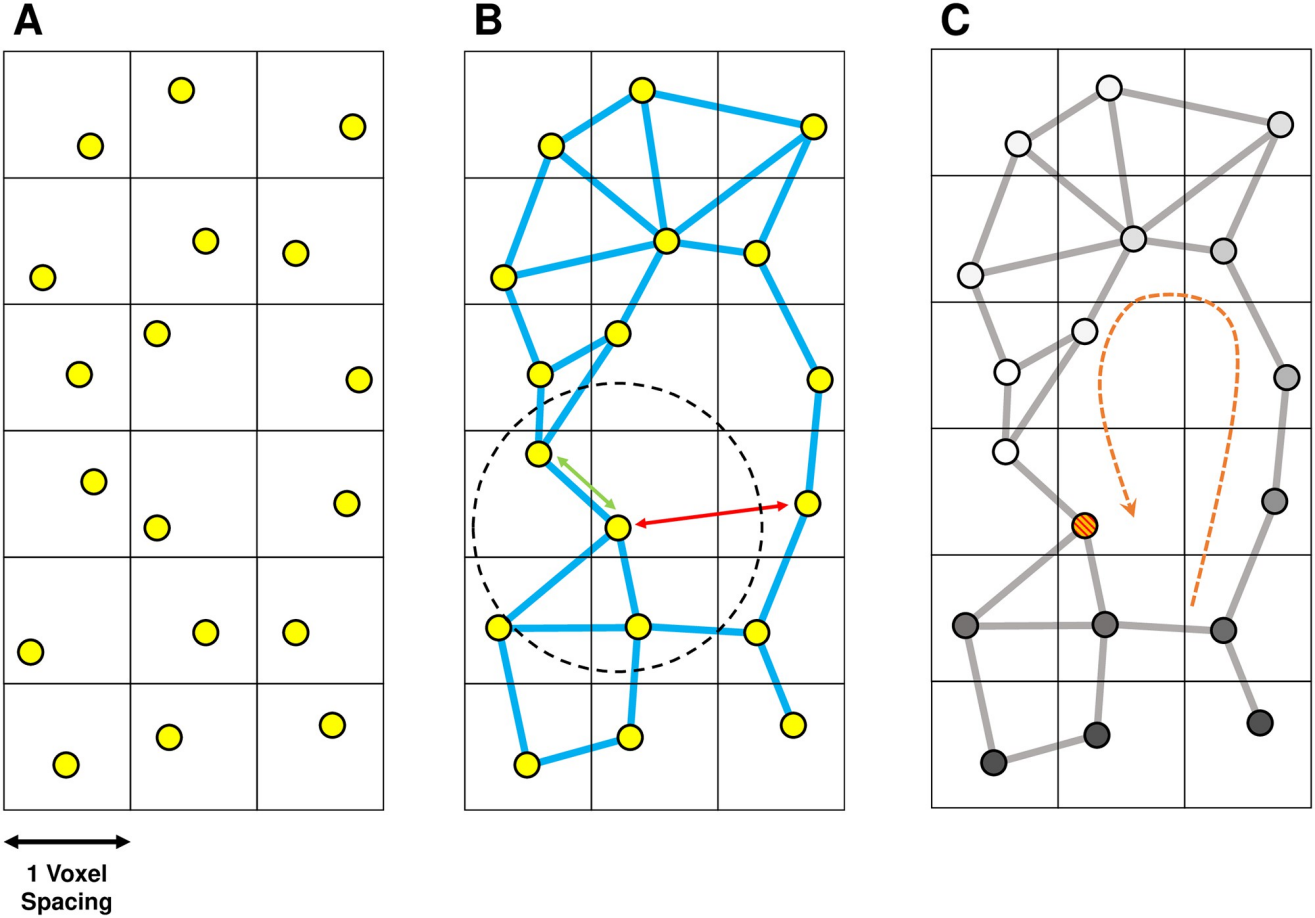
A 2d schematic demonstrating the construction of the spatial network using the fibre-less null model. The process is similar to Fig 3 where steps (A) and (B) are skipped. (A) Nodes are distributed uniformly in each voxel. (B) Nodes are connected according to a monotonically decreasing function of distance, *p*(*x*;*r*, *c*), Eq (1), where *c* is a characteristic distance (dashed circle). There is no directional bias in the connection probability (blue edges only). Nodes which are closer together than the characteristic distance (green arrows) are more likely to be connected than those which are far apart (red arrows). (C) The micro-reentrant substrate is identified using the DDM.

For comparison purposes, we consider a null model where the spatial network excludes fibre orientation. All other geometric information is retained. The fibre-less spatial network is generated analogously to the fibre model, with the omission of the initial fibre tractography steps, illustrated schematically in Fig 4. Nodes are randomly distributed across each atrial mesh at a density of approximately 1 node per voxel and are added to the spatial network if the distance between the new node and any existing nodes is greater than or equal to *d*_*sep*_ = 0.7.

To construct the spatial network, nodes are connected using the same distance dependent probability function as the fibre model given in Eq (1). Unlike the fibre model, none of the nodes are already connected by edges which represent the underlying fibre structure of the atria (no pink edges); the probability of connecting two nodes is independent of their orientation.

For each atrial dataset, we generate spatial networks for three values of the characteristic coupling, corresponding to high, medium and low risk cases, see S3 Table in SM section 1.3.2 in S1 File. The characteristic coupling values for the fibre-less null model are significantly larger than for the fibre model since there are no permanent longitudinal edges.

### 3.4 Identifying the substrate for micro-anatomical reentry

For both the fibre and fibre-less models, we associate the substrate for micro-anatomical reentry with regions in the corresponding spatial network where closed loops exist of length *ℓ* ≥ *τ*. Here, *τ* represents a typical refractory wavelength. These are found by applying a discrete diffusion model (DDM) to the network, see SM section 1.4.

The DDM is related to several extremely simple physics models of micro-anatomical reentry [19–23]. These models are not electrophysiologically realistic models of AF. The strength of discrete models lies in the ease with which structural discontinuities can be modelled, in contrast to conventional models which are significantly complicated when no-flux boundary conditions are imposed at the local level [24]. This justifies the use of the DDM in the current context where we focus exclusively on the structural basis for micro-reentry. However, this approach is not suitable for studying the dynamics of micro-anatomical reentry. For a wider discussion of the role of model choice see [25].

### 3.5 Analysis methods

#### 3.5.1 Defining risk of micro-anatomical reentry

To derive a measure for the risk of micro-reentry in each spatial network, we use the DDM and assume that all nodes in the network are equally susceptible to conduction block. If each structure has a similar number of nodes which, if blocked, may initiate a micro-anatomical reentry, then the probability of initiating any given circuit is approximately constant. Hence, for a spatial network with *N* potential substrates, the rate, λ, at which new micro-reentries are initiated in the DDM is directly related to the number of substrates *N*. This argument follows directly from similar arguments in [21]. A mathematically precise formulation of this argument is provided in SM section 1.5. The rate parameter, λ, is used as our measure of the *global risk* of micro-anatomical reentry across the atria as a whole. A uniform probability of conduction block is likely an unrealistic assumption. However, realistic conduction block behaviour is difficult to obtain in the DDM framework. These issues are discussed in more detail in the limitations section.

To compute the risk of micro-anatomical in a given atrial region, we define the *local risk* for a specific voxel, *v*, by calculating the number of circuits which form in that particular voxel, *N*_*v*_, when sampling a fixed number of identified circuits, *N*. The rescaled rate of reentry for the voxel *v* is then given by

$$\begin{array}{c} {\tilde{R}}_{v}=\frac{\lambda \cdot N_{v}}{\sum_{v}N_{v}}, \end{array}$$ (2)

where the denominator provides a normalisation. If a specific atrial region is referred to as “at risk”, this signifies a non-zero value of the local risk, ${\tilde{R}}_{v}$, in that region. Note that summing the local risk over all the voxels in the atrial geometry gives the global risk, $\lambda =\sum_{v}{\tilde{R}}_{v}$.

Given that there are typically many more voxels in each atrial geometry than sampled circuits, we smooth the local risk using a 3d Gaussian convolution with a standard deviation of 5 voxels (∼ 1.5*mm*) when plotting onto the atrial surfaces.

#### 3.5.2 Spatial clustering of reentrant circuits

For each spatial network, we measure how spatially clustered the identified micro-reentrant substrate is. To do so, we sample 1000 voxels from the set of voxels in which micro-anatomical reentry is identified for each network and extract the coordinate of each sampled voxel. The number of independent spatial clusters is then derived by using the DBSCAN algorithm, a standard algorithm for spatial data clustering, see [26]. We set eps = 10 (≈ 3*mm*), which is the parameter controlling the distance between points for these to be considered in the same cluster. The number of points required for a cluster is set to 1. If only one cluster is identified by DBSCAN, this indicates that all 1000 sampled circuits in a given network fall within a single, small region of the atrial structure. Conversely, if 1000 clusters are identified, no two circuits are identified in the same location.

#### 3.5.3 Atrial wall thickness & occupied voxel fraction

Wall thickness is a common measurement used when analysing atrial geometry. One issue when calculating wall thickness is that it is not easily defined in the atrial bulk. On the surfaces, wall thickness can be defined as the distance through the atrial wall along the perpendicular surface vector (there are competing, but similar definitions). However, in the bulk many perpendicular surface vectors pass through the same voxel so that the thickness is not uniquely defined.

To resolve this issue, we introduce a novel measure analogous to wall thickness, the occupied voxel fraction (OVF), which quantifies the proximity of voxels to the atrial walls and captures differences in the local wall curvature and thickness gradients, see SM section 1.6. The measure is defined as the number of voxels within a radius $\tilde{r}=5$ (approximately 1.5mm) which are inside the atrial structure, normalised by the total number of voxels within a sphere of radius $\tilde{r}$. The OVF increases (decreases) if the atrial walls become thicker (thinner), and distinguishes between regions with convex, flat, or concave wall morphologies.

#### 3.5.4 Longitudinal connection fraction

For the spatial networks with fibre structure, we would like to associate the identified micro-reentrant substrate with the degree of local fibre alignment in a particular atrial region. Fibre alignment is not naturally defined in a spatial network. Therefore, we use the longitudinal connection fraction (LCF) as a proxy measure. This is defined as the average number of longitudinal connections (along the fibre direction), normalised by the total number of connections, either longitudinal or transverse. If the LCF in a voxel is large, then fibres in that region are well aligned. Conversely, if the LCF is small, the fibre structure is highly disordered in a local area. Note, however, that the LCF can increase both from an increase in the number of longitudinal connections, but also from a reduction in the number of transverse connections. Therefore, larger LCF does not necessarily imply more longitudinal connections. More detail is provided in SM section 1.7 where we validate that the LCF in each spatial network accurately reflects the expected degree of fibre misalignment measured in the underlying data.

It is interesting to note that we can compare the LCF to the degree of microscopic anisotropy, calculated using Eq (1), at the highest level of spatial coupling where micro-anatomical reentry is inducible in our spatial networks. This demonstrates that the onset of micro-reentry in our spatial networks occurs at approximately the same degree of structural anisotropy as found experimentally by Spach *et al*. [27], see SM section 1.8 for details.

## 4 Results

### 4.1 Spatial distribution of identified micro-reentrant substrate

**Fig 5:**
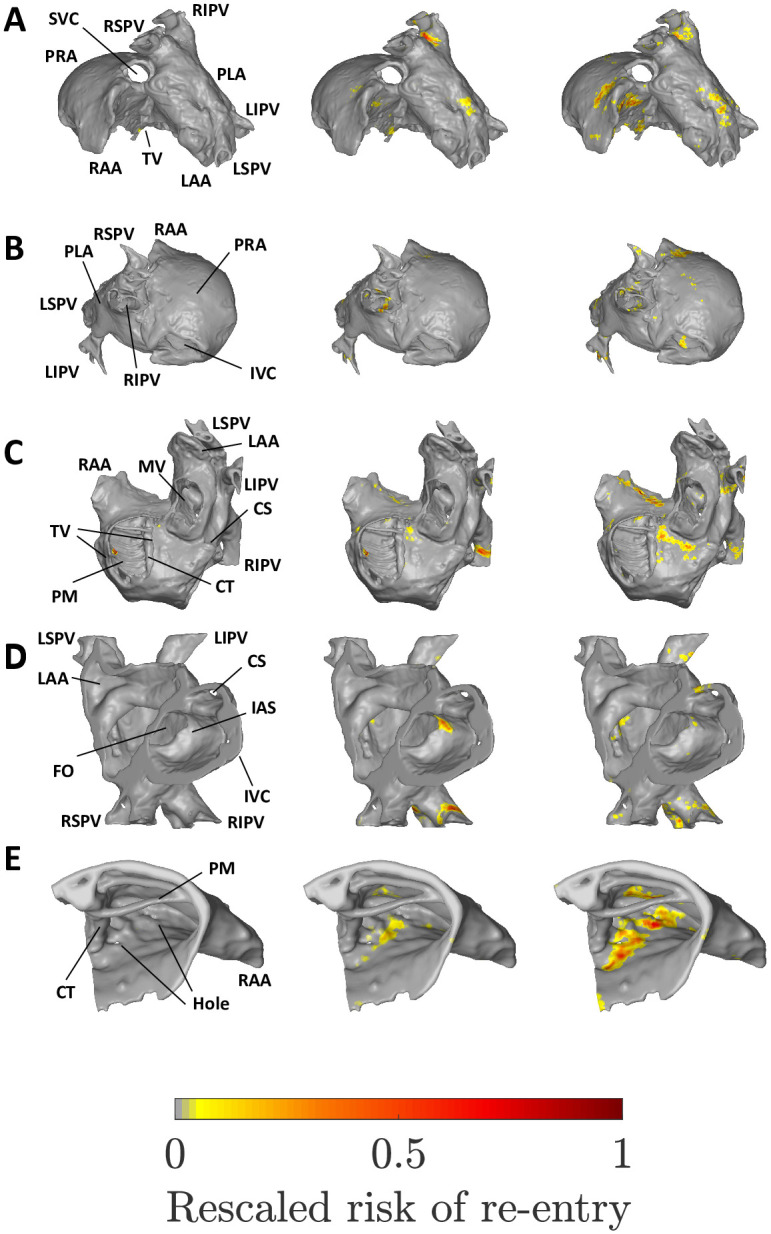
The micro-reentrant substrate for the human atria at low (left column), medium (middle) and high global risk of micro-reentry (right). A: Superior view. B: Posterior view. C: Inferior view. D: Cut-through. E: Superior right atrial zoom. IVC/SVC: Inferior/superior vena cava. PRA/PLA: Posterior right/left atrium. RAA/LAA: Right/left atrial appendage. TV/MV: Tricuspid/mitral valve opening. CS: Coronary sinus. FO: Fossa ovalis. PM: Pectinate muscles. CT: Crista terminalis. IAS: Inter-atrial septum. RIPV/LIPV/RSPV/LSPV: Right/left, inferior/superior, pulmonary vein.

**Fig 6:**
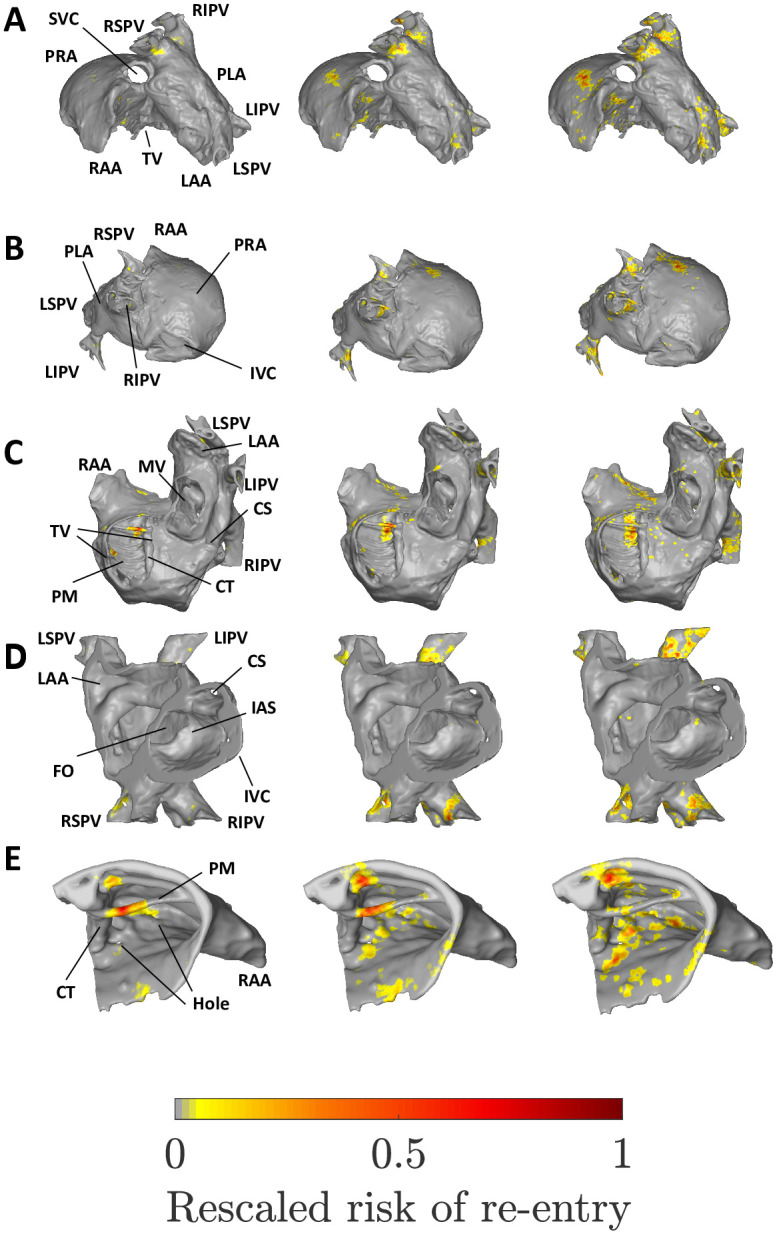
The micro-reentrant substrate for the human atria using the fibre-less null model at low (left column), medium (middle) and high global risk of micro-reentry (right). A: Superior view. B: Posterior view. C: Inferior view. D: Cut-through. E: Superior right atrial zoom. See Fig 5 for labels.

Figs 5 & 6 show the substrate identified as susceptible to micro-reentry in the human atria using the fibre and fibre-less models respectively, at low, medium and high global risk (progressively larger reentry rate, λ). Note that low, medium and high global risk refers to the overall risk of micro-reentry across the whole atrial tissue, and does not refer to whether specific regions identified are at low or high risk (which is captured by the local risk, ${\tilde{R}}_{v}$). The spatial distributions of local risk for the sheep atria are shown in SM section 2.1. How the differences in our datasets affect our results is discussed in SM section 2.2.

The reader should be conscious that the local risk distributions shown do not represent the regions at risk for a single atrial tissue, but rather show the collation of the local risk regions identified across 1000 randomly generated spatial networks. If a region is highlighted in the left column, the low global risk case, this may be interpreted as an area which is susceptible to micro-reentry even if the fibrosis density is low (but non-zero). Regions identified in the middle and right columns are regions susceptible to micro-reentry at moderate and high fibrosis densities respectively. This illustrates a key result: different regions of the atria are susceptible to micro-reentry at different fibrosis densities. In particular, regions may have a characteristic fibrosis density range within which they are susceptible to micro-reentry; if the fibrosis density is too high or low these regions will not exhibit micro-reentry.

#### 4.1.1 Micro-reentrant substrate in spatial network with fibre structure

At low global risk, micro-reentry is exclusively observed along a spatially isolated ridge of the pectinate muscles (PMs) in the right atrium, see Fig 5. At medium global risk, the pulmonary veins (PVs), in particular the right inferior pulmonary vein (RIPV), emerge as the dominant micro-reentrant substrate. Secondary substrates include the junction of the left LSPV and the PLA, and the inter-atrial septum (IAS). However, the hole in the IAS may play a role in it emerging as a local risk region. An additional strip of low local risk is observed in the superior right atrium, adjacent to the crista terminalis (CT), see Fig 5(E). The strip in question lies between two small holes in the atrial dataset, with sub-millimetre diameters.

At high global risk, the susceptible substrate is significantly more diffuse than at lower global risk. However, there remain multiple regions with very low or zero local risk, in particular the posterior right atrium (PRA), left atrial appendage (LAA) and inferior vena cava (IVC). The dominant local risk substrate lies along the strip between two small holes in the superior right atrium. Secondary regions of high local risk are maintained in the PVs, with local risk migrating slightly further from the LSPV junction into the PLA. The opening of the the coronary sinus forms a new substrate which is not observed at higher coupling. The local risk observed previously at the IAS and PMs is largely absent.

#### 4.1.2 Micro-reentrant substrate in spatial network without fibre structure

The local risk substrate identified by the fibre-less null model, see Fig 6, is qualitatively similar to the local risk regions identified in the fibre model. Key local risk regions including the superior right atrium and the PV sleeves are common to both models. Likewise, the PRA, LAA and IVC are not susceptible to micro-reentry in either model.

At low global risk, the dominant local risk substrate in the fibre-less null model is confined to two spatially isolated PM ridges, one of which exhibits local risk in the fibre model. Additional diffuse local risk is observed in the superior right atrium and in the right PV sleeves. At medium global risk, the low local risk substrate is retained, with additional local risk in all four PVs and diffuse risk across the superior right atrium. These local risk regions are consolidated at high global risk, with a small reduction in local risk observed along the spatially isolated PV ridges. The substrates observed for the fibre model along the coronary sinus (CS) opening and at the IAS are not observed in the fibre-less null model.

### 4.2 Clustering of micro-reentrant substrate

**Fig 7:**
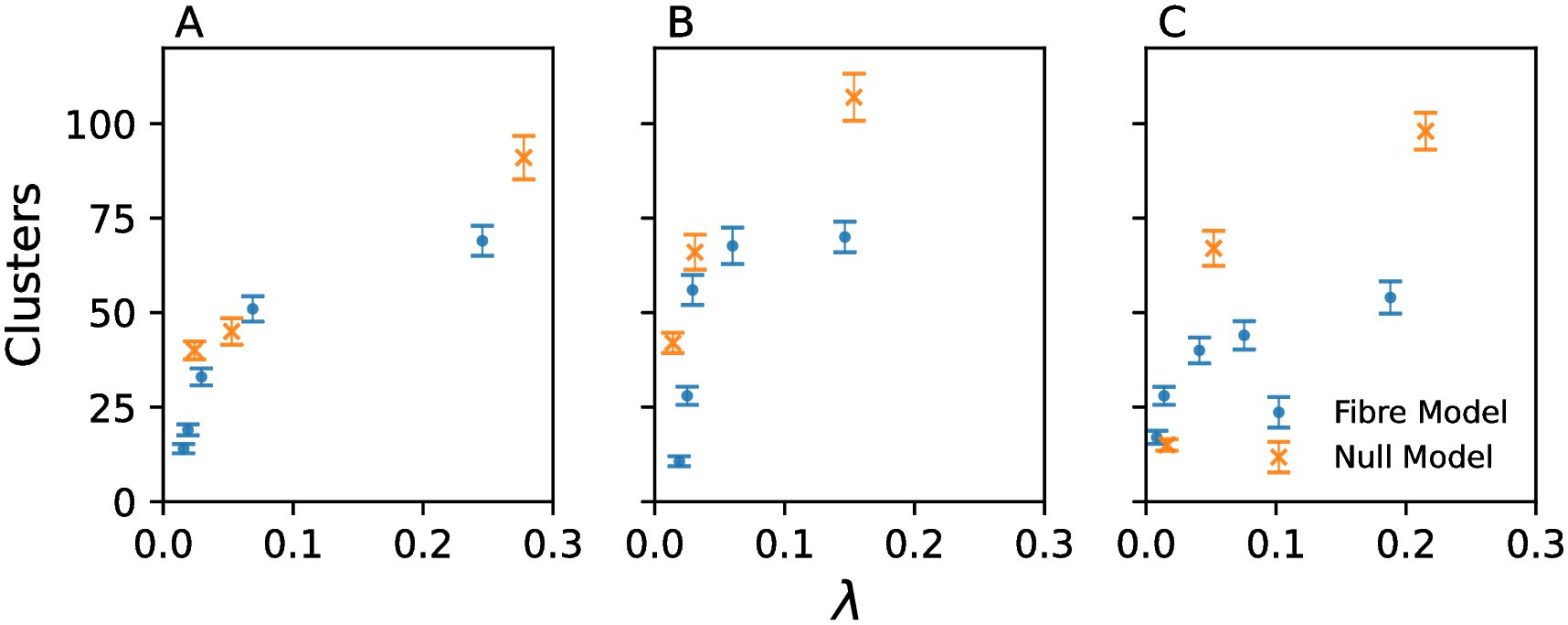
The number of independent spatial clusters identified as at local risk of micro-anatomical reentry. Data for the fibre model (blue points) and the fibre-less null model (orange crosses) for (A) the human atria, (B) the healthy sheep atria, and (C) the HF sheep atria, as a function of the global risk parameter, λ. Each clustering value is calculated from 1000 sampled micro-reentrant circuits. Error bars from bootstrapping.

The observed micro-reentrant risk substrates for the fibre and fibre-less models suggest that micro-reentry is spatially confined for both models at low global risk, but that the substrate covers a wider area as the global risk of micro-reentry increases. Fig 7 shows that the substrate is more clustered for the fibre model than for the fibre-less null model. One possible interpretation of this result is that if all micro-reentrant circuits are confined to a small number of clusters, these will be easy to destroy or isolate via catheter ablation. However, if circuits are widely distributed, they will be more difficult, if not impossible, to destroy or isolate.

### 4.3 Occupied voxel fraction

**Fig 8:**
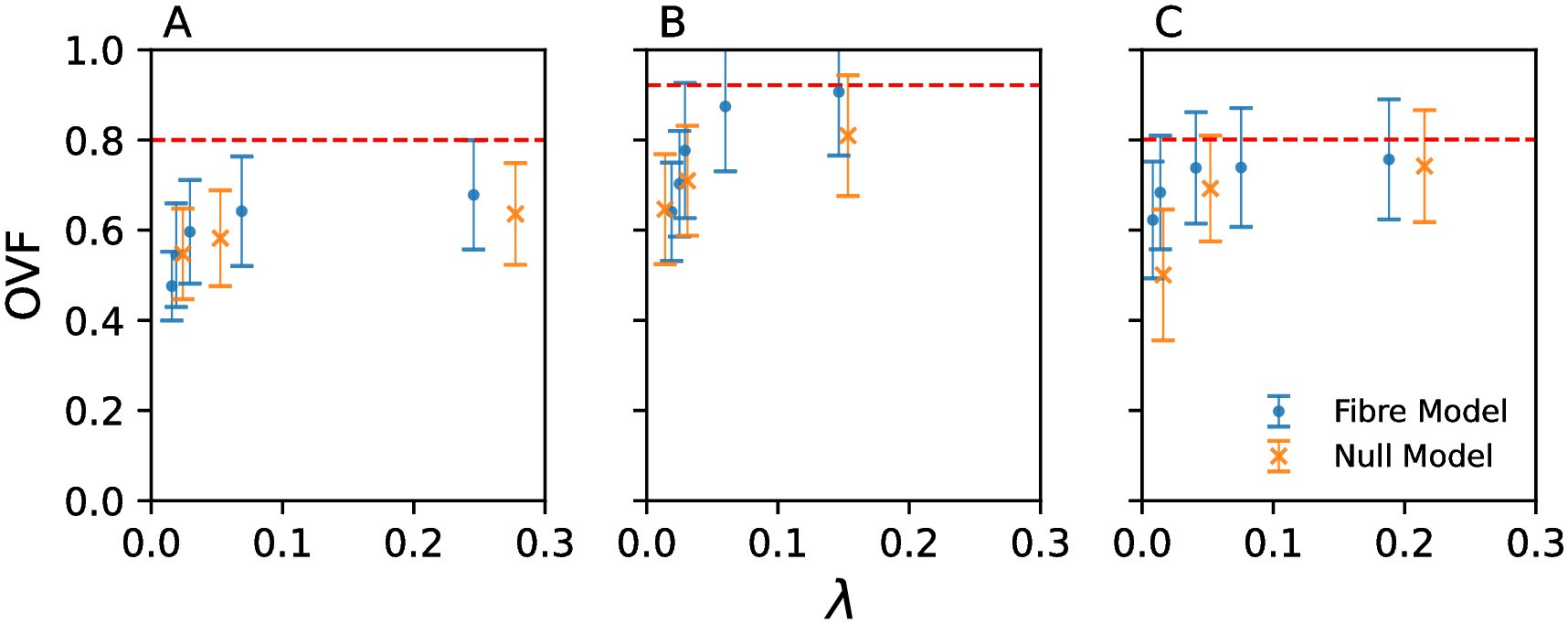
The occupied voxel fraction averaged over voxels where micro-reentry is detected. Data for the fibre model (blue points) and the fibre-less null model (orange crosses) for (A) the human atria, (B) the healthy sheep atria, and (C) the HF sheep atria, as a function of the global risk parameter, λ. The dashed line in each subfigure is the average value of the occupied voxel fraction for all the voxels in the atrial geometry.

Fig 8 shows the average occupied voxel fraction for the identified micro-reentrant substrates in the fibre and fibre-less models. For all three atrial datasets, the figure demonstrates that at low global risk, the micro-reentrant substrate is confined to regions with low OVF, significantly below the average value for each dataset. As global risk increases (increasing λ), the mean OVF increases. This indicates that the bias to thin convex regions of the atria is reduced as edges are progressively removed in each spatial network. Framed in terms of fibrosis densities, this result illustrates that regions with low OVF are susceptible to micro-reentry at a lower fibrosis density than regions with larger OVF. These results are supported by statistical analysis in SM section 2.3.

### 4.4 Comparing fibre and fibre-less micro-reentrant substrates

**Fig 9:**
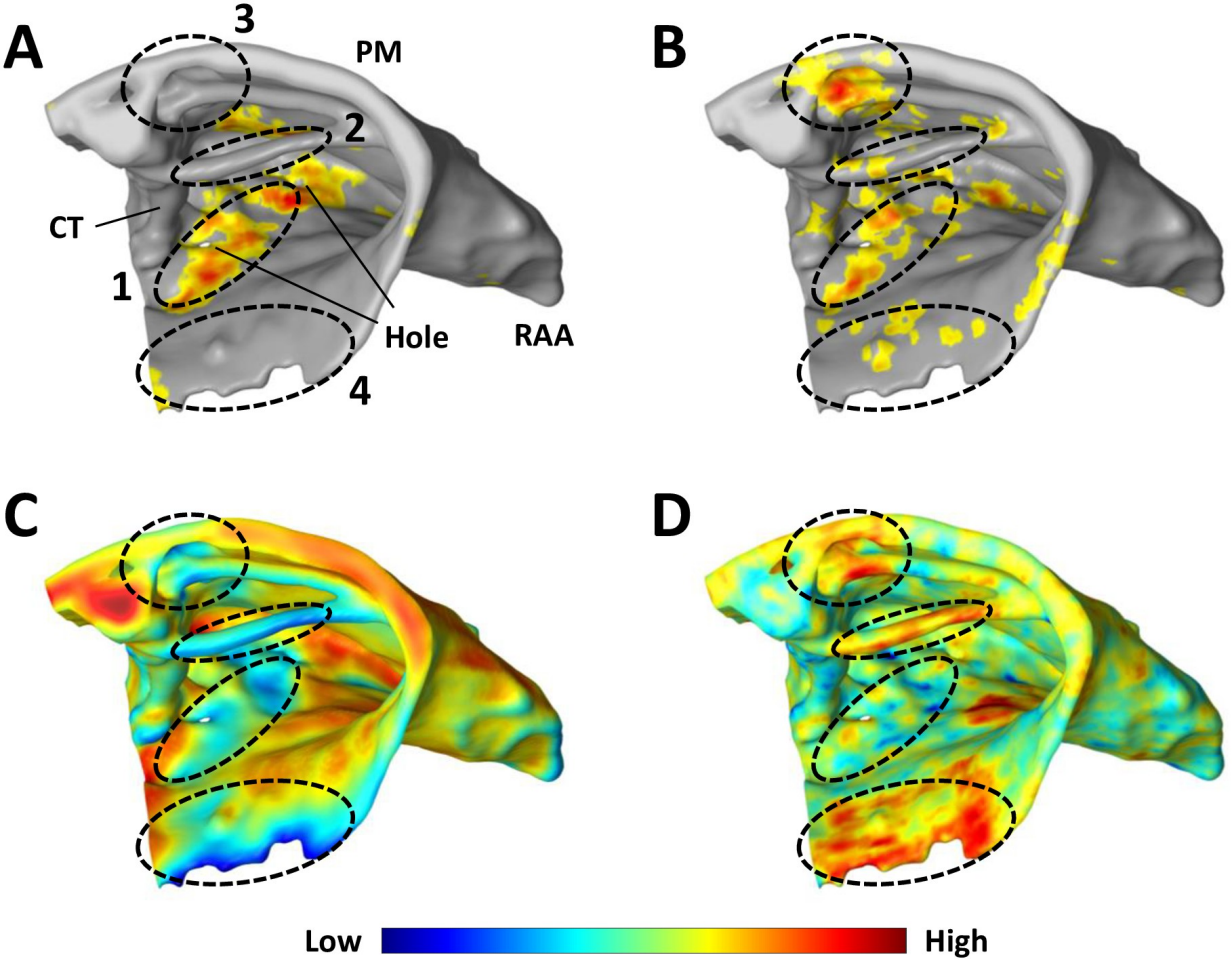
A view of the human superior right atrium illustrating factors influencing the spatial distribution of local risk. Risk regions for (A) the fibre model, and (B) the fibre-less null model. (C) The occupied voxel fraction (OVF). (D) The longitudinal connection fraction in the fibre model (LCF). Regions (1–4) are referenced in the main text. Colourbar limits: OVF ∈ [0.3, 0.9], LCF ∈ [0.4, 0.8].

Fig 9 shows an example of the micro-reentrant substrate for the fibre and fibre-less models alongside their corresponding OVF and LCF values in the superior right atrium. The figure is for the human atria at high global risk (right column in Figs 5 and 6) where the bias to thin atrial regions is small but non-zero.

In the superior right atrium, the micro-reentrant substrate at high global risk is concentrated along a strip between two small structural holes (region 1) for the fibre model, Fig 9(A). This region exhibits moderate risk in the fibre-less null model, Fig 9(B), with additional risk along the spatially isolated PM (region 2), the junction of the CT and a second PM ridge (region 3), and around the opening of the TV (region 4).

Region 2 exhibits low local risk in the fibre model, despite being a low OVF region, but is the dominant risk region for the fibre-less null model when the rate of micro-reentry is low or medium, see Fig 6(E) left and centre columns. This is because the strong longitudinal coupling in region 2 suppresses micro-reentry in the fibre model. This effect is absent in the null model where fibre structure is omitted. Region 2 is most susceptible to micro-reentry in the fibre-less null model at a low and moderate fibrosis densities, with the local risk reducing at higher densities. Regions 3 and 4 show very low or zero local risk in the fibre model.

Comparing the identified risk regions to the OVF values shown in Fig 9(C), we note that regions 1–4 all exhibit low OVF values (blue on the colour scale). Of the four regions identified, we observe that the LCF values, Fig 9(D), in region 1 are low (blue), indicating fibre misalignment, whereas strong longitudinal coupling is observed in regions 2–4. This suggests that the local risk of micro-reentry is suppressed in the fibre model in regions with high LCF, but can be enhanced in regions with low LCF.

**Fig 10:**
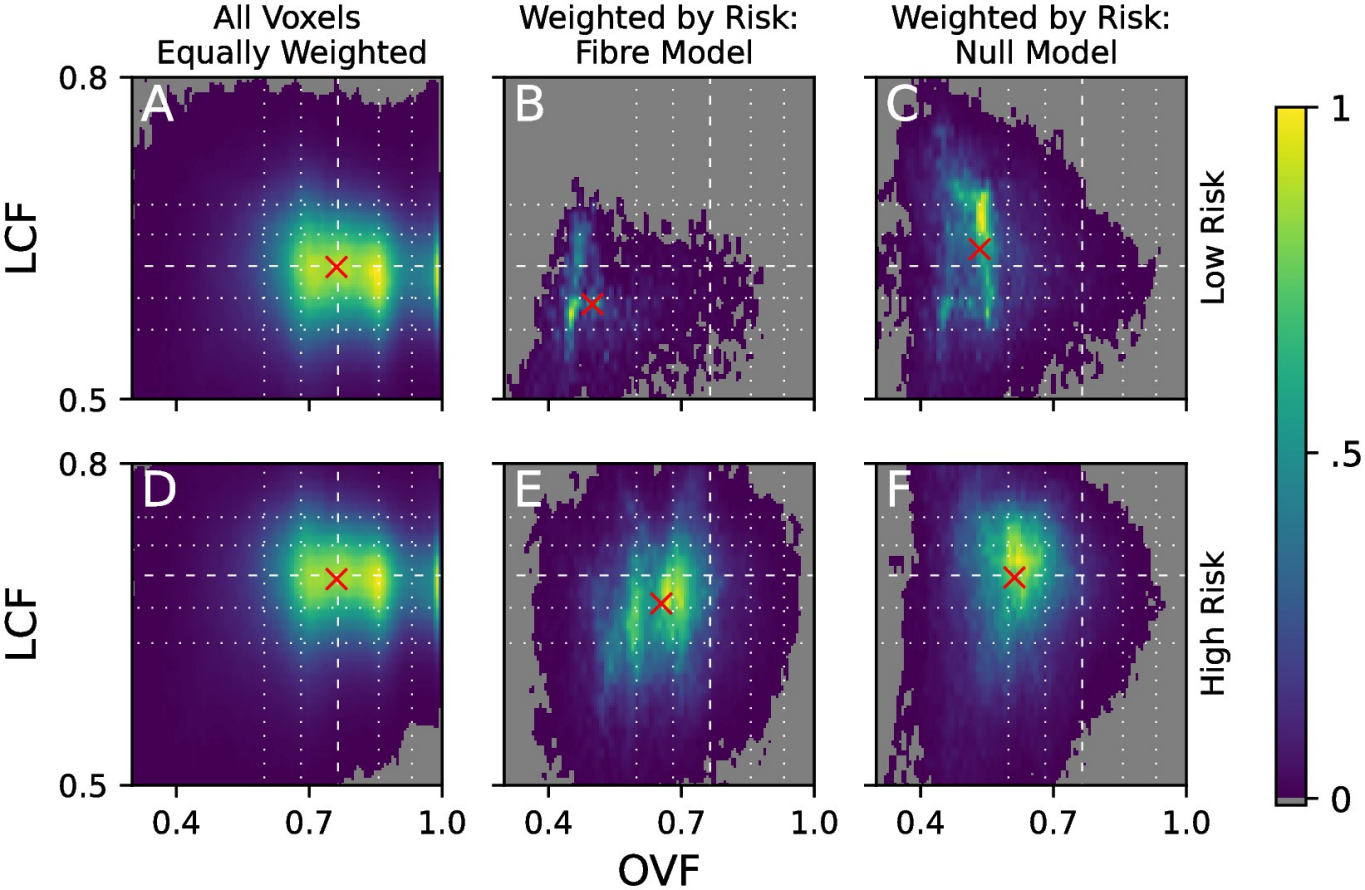
The distribution of LCF and OVF values in the human atria at low (A-C) and high (D-F) global risk. (A) & (D) The distribution across all the voxels in the human atria. (B) & (E) The distribution weighted by the local risk of reentry in each voxel for the fibre model. (C) & (F) The equivalent for the fibre-less null model. Note, the LCF is not defined for the fibre-less null model, but for illustrative purposes we include the LCF for the equivalent fibre model. Dashed lines: median. Dotted lines: 10^*th*^, 25^*th*^, 75^*th*^ and 90^*th*^ percentiles. Colourbar indicates voxel density relative to distribution maximum; regions in grey correspond to a relative density of zero. Red crosses: weighted mean of distribution.

These patterns can be quantified by plotting the distribution of LCF and OVF values across all the voxels in the human atria. Fig 10(A) & 10(D) show the distribution where all voxels are equally weighted (i.e., we count the number of voxels in the whole geometry with a specific value of the OVF and LCF) at (A) low and (D) high global risk. In Fig 10(B) & 10(E) these distributions are weighted by the local risk (i.e., we sum the local risk, ${\tilde{R}}_{v}$, over all voxels with fixed OVF and LCF) of micro-reentry observed in each voxel for the fibre model. The equivalent is shown in Fig 10(C) & 10(F) where voxels are weighted by their local risk in the fibre-less null model. Although the LCF is not defined for the fibre-less null model, it is illustrative to label null model voxels according to the corresponding fibre model LCF values.

Recall that the OVF is a geometric property of the atrial structure which is unaffected by the choice of coupling parameter, *c*, used to initialise the spatial networks. Hence, the distributions in Fig 10(A) & 10(D) align along the OVF axis. However, the LCF is not independent of the coupling parameter *c* used to initialise each network, and, consequently, is not independent of the global risk, λ. Therefore, the distributions in Fig 10(A) & 10(D) do not align along the LCF axis since an increase in global risk is associated with a decrease in transverse coupling, *c*. This is equivalent to an increase in the LCF.

If the distribution of local risk is unbiased by OVF or LCF, the distributions in Fig 10(B), 10(C), 10(E) & 10(F) should follow the same patterns shown in Fig 10(A) & 10(D). For the fibre model at low global risk, Fig 10(B), the voxel distribution is significantly skewed for both the OVF and the LCF with the weighted mean falling in the first OVF decile and the first LCF quartile. For the fibre-less null model at low global risk, Fig 10(C), the mean OVF value also falls in the first decile, but there is a small skew to LCF values larger than the median. This is an indirect effect and is explained by the strong longitudinal coupling along spatially isolated regions such as the PMs.

For both the fibre and fibre-less models the local risk density lies almost exclusively in the first OVF quartile, with negligible density at higher OVF values. In contrast, LCF values are clearly skewed in the fibre model, although some local risk density remains at larger LCF values. This implies that at low global risk, the OVF is the dominant factor in determining the local risk substrate, with fibre structure playing an important secondary role.

As the global risk of micro-reentry increases, we observe that the bias to low OVF values remains, but is slightly reduced, see Fig 10(C) & 10(F). However, the local risk of micro-reentry is still completely suppressed at the largest OVF values. For the fibre model, a small bias towards reduced LCF is observed. In contrast, the fibre-less null model shows minimal bias in the distribution of LCF values. Statistical arguments supporting the above discussion are provided in SM section 2.3.

Before proceeding, it is important to address what appears to be a contradiction. Comparing either Fig 10(B) to 10(C), or Fig 10(E) to 10(F), reveals how longitudinal coupling (high LCF) has a protective effect, reducing the local risk of micro-reentry, relative to regions with lower LCF. However, if we compare the case of low global risk in Fig 10(A)–10(C), to the case of high global risk, Fig 10(D)–10(F), it appears the the increase in global risk is associated with a shift in the voxel distributions from low LCF to higher LCF. This would would seem to imply, incorrectly, that longitudinal connections enhance the local risk of micro-reentry.

To understand this, note that there are two ways in which the LCF may increase: Firstly, if we fix the number longitudinal connections in the atrial geometry and progressively remove transverse connections, this will result in the average LCF increasing since the relative abundance of longitudinal connections increases. Conversely, if we fix the number of transverse connections and increase the number of longitudinal connections, this will result in an increase in the LCF. The former is the mechanism relevant when comparing the low global risk case to the high global risk case, and is not associated with an increase in the number of longitudinal connections. However, the latter is the mechanism relevant in a given atrial geometry where transverse coupling is effectively fixed, but regions with higher LCF have more longitudinal connections than regions with lower LCF. It is this second mechanism that is associated with the protective effect of longitudinal connections.

## 5 Discussion

In this paper, we introduce a novel computational approach for analysing how the structural substrate for micro-anatomical reentry may develop as the atria accumulate diffuse interstitial fibrosis. The technique combines spatial networks with fibre tractography, and assumes that interstitial fibrosis accumulation can be modelled as the decoupling of a spatial network, a concept closely related to spatial percolation [12]. Although our work focuses on micro-anatomical reentry, we believe our approach is of general interest, offering new methods for modelling atrial fibre structures, as well as metrics for quantifying longitudinal coupling and structural curvature.

Many studies investigate the electro-anatomical basis for micro-reentry as a potential mechanism of AF, although robust clinical evidence to support the mechanism is still lacking. Broadly speaking, such studies fall into three categories. In the first, the substrate can be identified directly, given sufficiently high resolution imaging, by observing the presence of a micro-anatomical driver. Examples include [7, 9], identifying several key factors in the formation of the reentrant substrate. Of particular note are the orientation of muscle fibres, the structure, thickness, and thickness gradients of the atria, and the accumulation of fibrosis, particularly interstitial fibrosis [28].

Attempting to quantify these factors, the authors in [9] applied optical mapping to explanted human atria to identify how specific values of wall thickness and fibrosis burden correlate to the location of the known micro-reentrant substrate [9]. Driver regions were found to correlate well with areas of 20–30% wall thickness and 20–30% fibrotic burden, notably the junction of the RIPV and PLA, and between the CT and fibrotically insulated PMs. Fibre misalignment was also implicated, specifically between the CT and the PMs which also exhibit abrupt changes in the local wall thickness. However, these studies took a largely static view of the micro-reentrant substrate, not considering how the substrate may change under electrical or structural remodelling. Note that these studies have also been criticised for inducing drivers under physiologically unrealistic doses of pinacidil for shortening the action potential duration.

In the second category, the substrate for micro-reentry has been established in the atria, but is hidden due to the lack of a clearly observable driver. One recent study has addressed this problem by noting that the visible micro-reentrant substrate varies strongly with variable atrial refractoriness [29]. Hence, the authors were able to demonstrate that the hidden micro-reentrant substrate could be unmasked and stabilised by shortening atrial refractoriness with adenosine. Such changes, induced pharmacologically, are analogous to some of the electro-mechanical changes that may be expected over time from atrial remodelling [4]. However, a robust framework for predicting the future micro-reentrant substrate is still lacking.

No study is yet to address the third category which is to predict how the substrate for micro-reentry will develop in the future on a patient-specific basis, a problem directly related to the increasing need for arrhythmic risk stratification [30].

In this proof of concept—which we acknowledge is far from clinical applicability—we have attempted to take a step towards addressing this and ask how the substrate for micro-reentry may develop over time. Specifically, we ask how different parts of the atria are susceptible to micro-reentry at different characteristic fibrosis ranges; some regions of the atria, particularly those where the atrial walls are thin and there is significant fibre misalignment, may be susceptible to micro-reentry at a low fibrotic density, whereas other regions may require higher fibrosis densities before micro-reentry can be induced. Such an approach is currently only possible in-silico, primarily due to ongoing challenges with accurate in-vivo atrial imaging [31].

Our results implicate the role of wall thickness and the misalignment of fibres, suggesting that thin atrial walls and reduced longitudinal fibre coupling both enhance the probability that a region is susceptible to micro-reentry at lower fibrosis densities, supporting the findings in [9]. However, our study suggests that the dependence on these factors evolves as the density of interstitial fibrosis grows in the atria. In particular, the spatial spread of the micro-reentrant substrate increases dramatically with small reductions in the spatial network coupling, and indicates that the bias to thin atrial regions with complex fibre morphology is reduced as the micro-reentrant substrate becomes more spatially diverse. Many of the specific regions highlighted as local risk substrates for micro-reentry in [9, 29], such as PM ridges and the superior right atrium, naturally emerge as local risk substrates using our method, most likely due to their position in thin atrial regions.

One explanation for the importance of wall thickness, fibrosis density, and local wall curvature is that driver regions emerge from percolation-like dynamics where the critical fibrosis density is dependent on the thickness of the structure studied [32, 33]. This in turn may result in micro-anatomical circuits anchoring to the atrial surfaces in paroxysmal AF but distributing across the atrial wall in persistent AF [34]. Aside from micro-reentry, modelling fibrosis using percolation-style distributions is known to perform particularly well when simulating patterns of AF maintenance [35], and has been used to explain complex fractionated electrograms and reentries in fibrotic border zones [36, 37].

In the wider AF literature, a number of computational studies consider the role of atrial structure on AF dynamics. In most cases, these studies investigate how structural factors affect electrical wavefront dynamics by solving the mono- or bidomain equation coupled with a suitable atrial cell model [33, 38–43]. However, even the most advanced patient-specific modelling methodologies suffer from the continued struggle to extract precise fibre orientation data and high resolution fibrosis profiles (particularly diffuse interstitial fibrosis) in-vivo [6].

Until these issues are resolved, computational modelling must continue to be used as a platform for hypothesis testing, studying the dynamics of AF initiation and maintenance with a variety of methods and across scales. In particular, the clearest insights may be found from models which contrast the role of a specific electro-anatomical feature with null models in the absence of that feature. Examples include models with and without realistic tissue geometries [38], with and without patient-specific fibrosis [44], isotropic vs. anisotropic fibre structures [41], and continuous vs. discrete modelling choices [25]. We hope that the techniques introduced in this paper can add to the range of techniques employed for hypothesis testing in cardiac electrophysiology.

### 5.1 Micro-anatomical reentry in the wider context

The role of local drivers, and specifically micro-anatomical reentrant circuits, in initiating and maintaining AF is controversial. After initial findings showed promise in 2015 [7], some suggested that the mechanism may have “the potential to unify some of the previous discrepant observations” [8] on AF mechanisms. However, although research is ongoing, reproducible clinical evidence identifying micro-anatomical reentrant circuits in patients is still lacking. Despite this, it is important to reiterate that the size of micro-reentrant circuits are postulated to be at, or below, the spatial resolution which can be resolved with conventional multi-electrode mapping [7, 9]. This may hamper attempts to acquire clinical evidence in support of the mechanism.

Opposing the local drivers hypothesis, many believe that AF is maintained by spatio-temporal chaos in the atria, whereby wavefronts continually collide into each other producing new fibrillatory wavefronts [45–51]; evidence for the mechanism is extensive and extends beyond AF-specific studies to more general studies on spatio-temporal chaos in excitable systems [52–54]. In our view, there is no reason that local drivers cannot co-exist with other non-local mechanisms, especially given recent evidence for a spectrum of mechanisms at different levels of organisation during fibrillation [4]. This is important because the interaction between local drivers and spatio-temporal chaos may have a significant effect on whether the ablation of local drivers is a feasible strategy for terminating AF.

Spatio-temporal chaos may have a non-trivial impact on local driver regions, likely resulting in an increased rate of circuit termination as the fibrotic substrate becomes more spread out, but also resulting in AF becoming more turbulent and difficult to terminate. If local drivers can be ablated, this may be avoided. However, local ablation in this context with numerous drivers across a diffuse substrate may become practically impossible given that (1) circuits are short-lived due to chaotic propagation, (2) circuits are spatially diverse, and (3) even if circuits can be destroyed, damage to the atrial myocardium may promote turbulent electrical propagation, driving more persistent AF. Despite this, local ablation strategies may still be worth pursuing if they lower the probability of initiating AF from sinus rhythm, where micro-reentrant circuits may act as a trigger.

Whether robust evidence for the micro-reentrant driver mechanism can be established is a question for the future. However, even without this evidence, the methods introduced here may be valuable to the wider cardiac electrophysiology community, adding to the computational toolkit for hypothesis testing. In particular, the methods introduced for constructing spatial networks using fibre orientation data may be of general interest to the community. Equally, structural metrics including the occupied voxel fraction and the longitudinal connection fraction may be useful for studying atrial geometries and fibre structures. Finally, although we acknowledge the limitations of the method (see below), the discrete diffusion model used to propagate signals across our spatial networks may be of general use, particularly in cases of high computational complexity where more phenomenologically accurate models constrain the number of simulations that can be run.

### 5.2 Limitations

The aims of this study are highly focused, discussing the structural basis for micro-anatomical reentry only. Many other factors may affect the probability that a micro-reentrant circuit forms in a given region of the atria, including other forms of fibrosis and ionic remodelling. Our approach does not consider these factors and cannot exclude their importance to micro-anatomical reentry.

The key technical limitations of our work come under two main categories: (1) Imaging related limitations and (2) limitations arising from specific modelling choices in the construction of the spatial networks.

In our view, one of the most important limitations relates to the datasets used for analysis. In particular, the human fibre dataset has undergone extensive smoothing, lacking realistic local heterogeneity in the fibre structure; the observed heterogeneous regions are likely artefacts from the synthetic fibre generation method. Similarly, the atrial geometry is heavily smoothed, lacking fine structural detail in regions such as the RAA and LAA, but with numerous structural holes across the geometry. Any future work must ensure that predictions are not simply an artefact of the data acquisition and generation process.

Although using high resolution fibre maps like those in [16, 17] may avoid these issues, acquiring such data is not feasible in-vivo, remaining a major ongoing research challenge [55, 56].

Assuming the imaging data used is of an acceptable quality and accuracy, a number of limitations arise in the fibre map modelling process. Firstly, the fibre tractography methods applied to generate global fibre tracts from local fibre orientation data are only an approximation of reality. Without performing precise histology to determine the position of global fibre tracts in our model, not histology focusing solely on local fibre orientation, it is not possible for us to explicitly validate this approach. Since the imaging data used was acquired from previous studies, such a process is not possible in the current work but may be possible in future studies. As a simple check of the robustness of our results, the full simulation pipeline, including the regeneration of fibres from a different set of random seed points, was carried out three times for the healthy sheep atria, showing no evidence that this significantly affected our final results.

Once fibre tracts are generated, nodes are placed along the fibres, and are coupled to nearby nodes based on their separation. The attachment function to connect two nodes is uniform across the atrial structure and does not consider any possible differences in the local connectivity of cardiomyocytes. Likewise, the characteristic coupling, *c*, that is used in each model is constant across the atrial structure. This can be thought of as applying a uniform density of interstitial fibrosis across the atria. It is known that not all regions of the atria are equally susceptible to the accumulation of fibrosis [57], and likewise, different forms of fibrosis accumulate differently across the atria [58]. Our assumption of uniformity is a reasonable first approximation in the absence of patient-specific information regarding the distribution of interstitial fibrosis which may not be visible with LGE-MRI. We do not consider any macroscopic fibrosis in the model. Such information was not available with the datasets acquired, but should be considered in any future work. However, how to accurately map imaging-derived fibrosis densities to precise spatial network edge densities is so far unclear and will likely pose a future technical challenge.

Once a spatial network has been constructed, the micro-reentrant substrate is identified by applying a simple discrete diffusion model on the network. The approach is loosely based on the techniques discussed in [20, 34], although we stress that its purpose here is strictly to identify locations in which isolated fibres result in micro-anatomical reentry, not to simulate AF dynamics. Assessing the ability of these structures to initiate and maintain AF would warrant a more detailed computational analysis with a phenomenologically accurate model which may be challenging at the resolution of our spatial network, and may struggle to produce large scale statistics. Despite this, the use of phenomenologically accurate propagation models may be necessary within our spatial network framework if we are to fully gauge and weigh up the relative impact of structural factors on the probability of micro-reentry, relative to other considerations such as the formation of functional blocks. Future studies using either method would benefit from a detailed comparison to experimentally acquired atrial functional data.

The regions that are identified by the discrete diffusion model are a set of isolated fibre tracts where unidirectional conduction block at a single node is sufficient to induce a micro-reentrant circuit. The method to apply conduction block assumes uniform risk of block, avoiding the need to specify special electrical properties in key atrial locations, and ignoring the potential impact of functional blocks from source-sink mismatch. In practice, it is known that electrical properties of cardiomyocytes vary significantly across the atria. For instance, the proarrhythmic conditions found in the PVs such as conduction velocity slowing and shortened action potential durations are not considered. However, our results do indicate that the PVs can emerge as a key risk substrate without the inclusion of electrical proarrhythmic effects.

Finally, before our approach can have real clinical relevance, it would benefit from further validation. We have demonstrated that the spatial networks accurately preserve the underlying properties of each atrial fibre map, and that our techniques ensure an even density of nodes across the network. For clinical relevance, it is important to validate the approach directly against experimental data, ideally with datasets with a history of micro-anatomical reentry. If possible, raw data should be available such that fibre maps can be generated using a range of methods at different levels of smoothing, and that the influence of small structural holes can be tested. Data may be acquired at different levels of interstitial fibrotic density to assess the validity of our method’s longitudinal predictions.

## 6 Conclusion

We have introduced a simple, proof of concept framework which attempts to study how the substrate for micro-anatomical reentry develops from the accumulation of interstitial fibrosis. The method, which is based on the application of percolation to spatial networks, suggests that the micro-reentrant substrate is critically dependent of local tissue geometry and areas of fibre misalignment. We suggest that the dependence on these factors is complex, continuously evolving with the absolute level of global micro-reentrant risk.

If robust clinical evidence can be found to support the micro-reentrant driver mechanism, and if our methods develop sufficiently, the approach introduced here may have potential for patient-specific risk stratification and personalised ablation strategies. However, our results also imply that the micro-reentrant substrate may become so spatially diverse, particularly at high fibrosis densities, that ablating these drivers may not be practically feasible.

## Supporting information

S1 File Supplementary information document. This file contains additional details regarding the methods used in this paper, as well as expanded results. In particular, this document includes details of atrial geometry thickness measurements, and figures showing the micro-anatomical risk substrates for the sheep atrial datasets.
(PDF)

## References

[1] Patel NJ, Atti V, Mitrani RD, Viles-Gonzalez JF, Goldberger JJ. Global rising trends of atrial fibrillation: a major public health concern. Heart. 2018;104(24):1989–1990. doi: 10.1136/heartjnl-2018-313350 29907645

[2] Calkins H, Hindricks G, Cappato R, Kim YH, Saad EB, Aguinaga L, et al. 2017 HRS/EHRA/ECAS/APHRS/SOLAECE expert consensus statement on catheter and surgical ablation of atrial fibrillation. EP Europace. 2017;20(1):e1–e160. doi: 10.1093/europace/eux274 PMC5834122 29016840

[3] Ng FS, Handa BS, Li X, Peters NS. Toward Mechanism-Directed Electrophenotype-Based Treatments for Atrial Fibrillation. Frontiers in Physiology. 2020;11:987. doi: 10.3389/fphys.2020.00987 33013435 PMC7493660

[4] Handa BS, Li X, Baxan N, Roney CH, Shchendrygina A, Mansfield CA, et al. Ventricular fibrillation mechanism and global fibrillatory organization are determined by gap junction coupling and fibrosis pattern. Cardiovascular Research. 2020. 10.1093/cvr/cvaa141 PMC7983010 32402067

[5] Boyle PM, Zghaib T, Zahid S, Ali RL, Deng D, Franceschi WH, et al. Computationally guided personalized targeted ablation of persistent atrial fibrillation. Nature Biomedical Engineering. 2019;3(11):870–879. doi: 10.1038/s41551-019-0437-9 31427780 PMC6842421

[6] Niederer SA, Lumens J, Trayanova NA. Computational models in cardiology. Nat Rev Cardiol. 2019;16(2):100–111. doi: 10.1038/s41569-018-0104-y 30361497 PMC6556062

[7] Hansen BJ, Zhao J, Csepe TA, Moore BT, Li N, Jayne LA, et al. Atrial fibrillation driven by micro-anatomic intramural re-entry revealed by simultaneous sub-epicardial and sub-endocardial optical mapping in explanted human hearts. Eur Heart J. 2015;36(35):2390–2401. doi: 10.1093/eurheartj/ehv233 26059724 PMC4568403

[8] Nattel S, Xiong F, Aguilar M. Demystifying rotors and their place in clinical translation of atrial fibrillation mechanisms. Nat Rev Cardiol. 2017;14(9):509–520. doi: 10.1038/nrcardio.2017.37 28383023

[9] Zhao J, Hansen BJ, Wang Y, Csepe TA, Sul LV, Tang A, et al. Three-dimensional integrated functional, structural, and computational mapping to define the structural “fingerprints” of heart-specific atrial fibrillation drivers in human heart ex vivo. Journal of the American Heart Association. 2017;6(8):e005922. doi: 10.1161/JAHA.117.005922 28862969 PMC5586436

[10] Krueger MW, Rhode KS, O‘Neill MD, Rinaldi CA, Gill J, Razavi R, et al. Patient-specific modeling of atrial fibrosis increases the accuracy of sinus rhythm simulations and may explain maintenance of atrial fibrillation. Journal of electrocardiology. 2014;47(3):324–328. doi: 10.1016/j.jelectrocard.2013.11.003 24529989

[11] McDowell KS, Vadakkumpadan F, Blake R, Blauer J, Plank G, MacLeod RS, et al. Methodology for patient-specific modeling of atrial fibrosis as a substrate for atrial fibrillation. Journal of electrocardiology. 2012;45(6):640–645. doi: 10.1016/j.jelectrocard.2012.08.005 22999492 PMC3515859

[12] Dall J, Christensen M. Random geometric graphs. Physical review E. 2002;66(1):016121. doi: 10.1103/PhysRevE.66.016121 12241440

[13] Barthélemy M. Spatial networks. Physics Reports. 2011;499(1-3):1–101. doi: 10.1016/j.physrep.2010.11.002

[14] Krueger MW, Schmidt V, Tobón C, Weber FM, Lorenz C, Keller DUJ, et al. Modeling Atrial Fiber Orientation in Patient-Specific Geometries: A Semi-automatic Rule-Based Approach. In: Metaxas DN, Axel L, editors. Functional Imaging and Modeling of the Heart. Berlin, Heidelberg: Springer Berlin Heidelberg; 2011. p. 223–232.

[15] Zhao J, Krueger MW, Seemann G, Meng S, Zhang H, Dössel O, et al. Myofiber orientation and electrical activation in human and sheep atrial models. In: 2012 Annual International Conference of the IEEE Engineering in Medicine and Biology Society; 2012. p. 6365–6368. 10.1109/EMBC.2012.6347450 23367385

[16] Zhao J, Butters TD, Zhang H, Pullan AJ, LeGrice IJ, Sands GB, et al. An Image-Based Model of Atrial Muscular Architecture: Effects of Structural Anisotropy on Electrical Activation. Circ Arrhythm Electrophysiol. 2012;5(2):361–370. doi: 10.1161/CIRCEP.111.967950 22423141

[17] Thomas B. An imaging pipeline for extracting atrial tissue architecture. University of Auckland. 2020.

[18] Merhof D, Sonntag M, Enders F, Hastreiter P, Fahlbusch R, Nimsky C, et al. Visualization of diffusion tensor data using evenly spaced streamlines. Vision, Modeling and Visualization. 2005; p. 257–264.

[19] Falkenberg M, Hickey D, Terrill L, Ciacci A, Peters NS, Christensen K. Identifying Potential Re-Entrant Circuit Locations From Atrial Fibre Maps. In: 2019 Computing in Cardiology (CinC). IEEE; 2019. p. Page–1. 10.22489/CinC.2019.102 PMC7279949 32514409

[20] Christensen K, Manani KA, Peters NS. Simple Model for Identifying Critical Regions in Atrial Fibrillation. Phys Rev Lett. 2015;114:028104. doi: 10.1103/PhysRevLett.114.028104 25635565 PMC4340556

[21] Ciacci A, Falkenberg M, Manani KA, Evans TS, Peters NS, Christensen K. Understanding the transition from paroxysmal to persistent atrial fibrillation. Physical Review Research. 2020;2(2):023311. doi: 10.1103/PhysRevResearch.2.023311 32607500 PMC7326608

[22] Bub G, Shrier A, Glass L. Spiral wave generation in heterogeneous excitable media. Physical review letters. 2002;88(5):058101. doi: 10.1103/PhysRevLett.88.058101 11863783

[23] Ciaccio EJ, Peters NS, Garan H. Use of an automaton model to suggest methods for cessation of intractable fibrillatory activity. Computers in biology and medicine. 2018;102:357–368. doi: 10.1016/j.compbiomed.2018.07.020 30097173

[24] Clayton R, Bernus O, Cherry E, Dierckx H, Fenton FH, Mirabella L, et al. Models of cardiac tissue electrophysiology: progress, challenges and open questions. Progress in biophysics and molecular biology. 2011;104(1-3):22–48. doi: 10.1016/j.pbiomolbio.2010.05.008 20553746

[25] Gokhale TA, Medvescek E, Henriquez CS. Modeling dynamics in diseased cardiac tissue: Impact of model choice. Chaos: An Interdisciplinary Journal of Nonlinear Science. 2017;27(9):093909. doi: 10.1063/1.4999605 28964161 PMC5568867

[26] Ester M, Kriegel HP, Sander J, Xu X. A Density-Based Algorithm for Discovering Clusters in Large Spatial Databases with Noise. In: Proceedings of the Second International Conference on Knowledge Discovery and Data Mining. KDD’96. AAAI Press; 1996. p. 226–231.

[27] Spach M, Dolber P, Heidlage J. Influence of the passive anisotropic properties on directional differences in propagation following modification of the sodium conductance in human atrial muscle. A model of reentry based on anisotropic discontinuous propagation. Circulation research. 1988;62(4):811–832. doi: 10.1161/01.RES.62.4.811 2450697

[28] Mikhailov AV, Kalyanasundaram A, Li N, Scott SS, Artiga EJ, Subr MM, et al. Comprehensive evaluation of electrophysiological and 3D structural features of human atrial myocardium with insights on atrial fibrillation maintenance mechanisms. Journal of Molecular and Cellular Cardiology. 2021;151:56–71. doi: 10.1016/j.yjmcc.2020.10.012 33130148 PMC7880876

[29] Hansen BJ, Zhao J, Helfrich KM, Li N, Iancau A, Zolotarev AM, et al. Unmasking Arrhythmogenic Hubs of Reentry Driving Persistent Atrial Fibrillation for Patient-Specific Treatment. Journal of the American Heart Association. 2020;9:e017789. doi: 10.1161/JAHA.120.017789 33006292 PMC7792422

[30] Arevalo HJ, Vadakkumpadan F, Guallar E, Jebb A, Malamas P, Wu KC, et al. Arrhythmia risk stratification of patients after myocardial infarction using personalized heart models. Nature communications. 2016;7(1):1–8. doi: 10.1038/ncomms11437 27164184 PMC4866040

[31] Sohns C, Marrouche NF. Atrial fibrillation and cardiac fibrosis. European heart journal. 2020;41(10):1123–1131. doi: 10.1093/eurheartj/ehz786 31713590

[32] Alonso S, dos Santos RW, Bär M. Reentry and ectopic pacemakers emerge in a three-dimensional model for a slab of cardiac tissue with diffuse microfibrosis near the percolation threshold. PloS one. 2016;11(11):e0166972. doi: 10.1371/journal.pone.0166972 27875591 PMC5119821

[33] Sachetto R, Alonso S, dos Santos RW. Killing Many Birds With Two Stones: Hypoxia and Fibrosis Can Generate Ectopic Beats in a Human Ventricular Model. Frontiers in Physiology. 2018;9:764. doi: 10.3389/fphys.2018.00764 29988469 PMC6024351

[34] Falkenberg M, Ford AJ, Li AC, Lawrence R, Ciacci A, Peters NS, et al. Unified mechanism of local drivers in a percolation model of atrial fibrillation. Physical Review E. 2019;100(6):062406. doi: 10.1103/PhysRevE.100.062406 31962501 PMC7314598

[35] Roney CH, Bayer JD, Zahid S, Meo M, Boyle PMJ, Trayanova NA, et al. Modelling methodology of atrial fibrosis affects rotor dynamics and electrograms. EP Europace. 2016;18:iv146–iv155. doi: 10.1093/europace/euw365 28011842 PMC6279153

[36] Vigmond E, Pashaei A, Amraoui S, Cochet H, Hassaguerre M. Percolation as a mechanism to explain atrial fractionated electrograms and reentry in a fibrosis model based on imaging data. Heart rhythm. 2016;13(7):1536–1543. doi: 10.1016/j.hrthm.2016.03.019 26976038

[37] Haissaguerre M, Shah AJ, Cochet H, Hocini M, Dubois R, Efimov I, et al. Intermittent drivers anchoring to structural heterogeneities as a major pathophysiological mechanism of human persistent atrial fibrillation. The Journal of physiology. 2016;594(9):2387–2398. doi: 10.1113/JP270617 26890861 PMC4850206

[38] Roy A, Varela M, Aslanidi O. Image-based computational evaluation of the effects of atrial wall thickness and fibrosis on re-entrant drivers for atrial fibrillation. Frontiers in physiology. 2018;9:1352. doi: 10.3389/fphys.2018.01352 30349483 PMC6187302

[39] Kharche S, Castro S, Thomas B, Colman M, Jarvis J, Smaill B, et al. Role of fiber orientation in atrial arrythmogenesis. In: Computing in Cardiology 2014. IEEE; 2014. p. 1041–1044.

[40] Roney CH, Bayer JD, Cochet H, Meo M, Dubois R, Jaïs P, et al. Variability in pulmonary vein electrophysiology and fibrosis determines arrhythmia susceptibility and dynamics. PLoS computational biology. 2018;14(5):e1006166. doi: 10.1371/journal.pcbi.1006166 29795549 PMC5997352

[41] Roney C, Bendikas R, Pashakhanloo F, Corrado C, Vigmond E, McVeigh E, et al. Constructing a Human Atrial Fibre Atlas. Annals of Biomedical Engineering. 2020. doi: 10.1007/s10439-020-02525-w 32458222 PMC7773625

[42] Yamazaki M, Mironov S, Taravant C, Brec J, Vaquero LM, Bandaru K, et al. Heterogeneous atrial wall thickness and stretch promote scroll waves anchoring during atrial fibrillation. Cardiovascular research. 2012;94(1):48–57. doi: 10.1093/cvr/cvr357 22227155 PMC3307378

[43] Aslanidi OV, Colman MA, Stott J, Dobrzynski H, Boyett MR, Holden AV, et al. 3D virtual human atria: A computational platform for studying clinical atrial fibrillation. Progress in biophysics and molecular biology. 2011;107(1):156–168. doi: 10.1016/j.pbiomolbio.2011.06.011 21762716 PMC3211061

[44] Roy A, Varela M, Chubb H, MacLeod R, Hancox JC, Schaeffter T, et al. Identifying locations of re-entrant drivers from patient-specific distribution of fibrosis in the left atrium. PLoS computational biology. 2020;16(9):e1008086. doi: 10.1371/journal.pcbi.1008086 32966275 PMC7535127

[45] Garrey WE. Auricular fibrillation. Physiological Reviews. 1924;4(2):215–250. doi: 10.1152/physrev.1924.4.2.215

[46] Dharmaprani D, Schopp M, Kuklik P, Chapman D, Lahiri A, Dykes L, et al. Renewal Theory as a Universal Quantitative Framework to Characterize Phase Singularity Regeneration in Mammalian Cardiac Fibrillation. Circulation: Arrhythmia and Electrophysiology. 2019;12(12):e007569. 31813270 10.1161/CIRCEP.119.007569

[47] Dharmaprani D, Jenkins E, Aguilar M, Quah JX, Lahiri A, Tiver K, et al. M/M/Infinity Birth-Death Processes—A Quantitative Representational Framework to Summarize and Explain Phase Singularity and Wavelet Dynamics in Atrial Fibrillation. Frontiers in Physiology. 2021;11:1786. doi: 10.3389/fphys.2020.616866 33519522 PMC7841497

[48] Dharmaprani D, Jenkins EV, Quah JX, Lahiri A, Tiver K, Mitchell L, et al. A governing equation for rotor and wavelet number in human clinical ventricular fibrillation: Implications for sudden cardiac death. Heart Rhythm. 2022;19(2):295–305. doi: 10.1016/j.hrthm.2021.10.008 34662707

[49] Chen J, Mandapati R, Berenfeld O, Skanes AC, Gray RA, Jalife J. Dynamics of wavelets and their role in atrial fibrillation in the isolated sheep heart. Cardiovascular research. 2000;48(2):220–232. doi: 10.1016/S0008-6363(00)00177-2 11054469

[50] Christoph J, Chebbok M, Richter C, Schröder-Schetelig J, Bittihn P, Stein S, et al. Electromechanical vortex filaments during cardiac fibrillation. Nature. 2018;555(7698):667–672. doi: 10.1038/nature26001 29466325

[51] Kay MW, Rogers JM. Epicardial rotors in panoramic optical maps of fibrillating swine ventricles. In: 2006 International Conference of the IEEE Engineering in Medicine and Biology Society. IEEE; 2006. p. 2268–2271. 10.1109/IEMBS.2006.260635 17946949

[52] Lechleiter J, Girard S, Peralta E, Clapham D. Spiral calcium wave propagation and annihilation in Xenopus laevis oocytes. Science. 1991;252(5002):123–126. doi: 10.1126/science.2011747 2011747

[53] Morris SW, Bodenschatz E, Cannell DS, Ahlers G. Spiral defect chaos in large aspect ratio Rayleigh-Bénard convection. Physical review letters. 1993;71(13):2026. doi: 10.1103/PhysRevLett.71.2026 10054564

[54] Tan TH, Liu J, Miller PW, Tekant M, Dunkel J, Fakhri N. Topological turbulence in the membrane of a living cell. Nature Physics. 2020;16(6):657–662. doi: 10.1038/s41567-020-0841-9

[55] Aronis KN, Ali R, Trayanova NA. The role of personalized atrial modeling in understanding atrial fibrillation mechanisms and improving treatment. International Journal of Cardiology. 2019;287:139–147. doi: 10.1016/j.ijcard.2019.01.096 30755334 PMC6513696

[56] Pashakhanloo F, Herzka DA, Ashikaga H, Mori S, Gai N, Bluemke DA, et al. Myofiber Architecture of the Human Atria as Revealed by Submillimeter Diffusion Tensor Imaging. Circulation: Arrhythmia and Electrophysiology. 2016;9(4):e004133. doi: 10.1161/CIRCEP.116.004133 27071829 PMC7035884

[57] Benito EM, Cabanelas N, Nuñez-Garcia M, Alarcón F, Figueras I Ventura RM, Soto-Iglesias D, et al. Preferential regional distribution of atrial fibrosis in posterior wall around left inferior pulmonary vein as identified by late gadolinium enhancement cardiac magnetic resonance in patients with atrial fibrillation. Ep Europace. 2018;20(12):1959–1965. doi: 10.1093/europace/euy095 29860416

[58] Anderson K, Sutton M, Lie J. Histopathological types of cardiac fibrosis in myocardial disease. The Journal of pathology. 1979;128(2):79. doi: 10.1002/path.1711280205 572867

