# Supplementary material for: Identifying locations susceptible to micro-anatomical reentry using a spatial network representation of atrial fibre maps
Source: PLoS One. 2022 Jun 23;17(6):e0267166. doi: 10.1371/journal.pone.0267166 (PMC9223322; doi:10.1371/journal.pone.0267166)
Supplement: S1 File — This file contains additional details regarding the methods used in this paper, as well as expanded results. In particular, this document includes details of atrial geometry thickness measurements, and figures showing the micro-anatomical risk substrates for the sheep atrial datasets. (PDF) [file pone.0267166.s001.pdf]

Supplementary Information: Identifying locations susceptible to micro-anatomical reentry using a spatial network representation of atrial fibre maps

April 2022

Max Falkenberg[1,2,3,*], James A Coleman[2], Sam Dobson[2], David J Hickey[2], Louie Terrill[2], Alberto Ciacci[1,2,3], Belvin Thomas[4], Arunashis Sau[3], Fu Siong Ng[3], Jichao Zhao[4], Nicholas S Peters[3], Kim Christensen[1,2,3]

1 Centre for Complexity Science, Imperial College London, London, United Kingdom
2 Department of Physics, Imperial College London, London, United Kingdom
3 ElectroCardioMaths Programme, Imperial Centre for Cardiac Engineering, National Heart & Lung Institute, Imperial College London, London, United Kingdom
4 Auckland Bioengineering Institute, The University of Auckland, Auckland, New Zealand

* max.falkenberg@protonmail.com

OVERVIEW

This supplementary document is split into two sections.

In section 1, we provide additional information on the methods underlying our approach. In section 1.1 we discuss the three atrial fibre datasets used in this paper, before outlining our choice of tractography algorithm in section 1.2. In section 1.3 we list the characteristic coupling values and corresponding risk parameters used to initialise our spatial networks. In section 1.4 we define the discrete diffusion model (DDM) which we use to identify the micro-reentrant substrate in each fibre map. In section 1.5 we outline how the DDM is used to define the risk of micro-reentry in each spatial network by defining the rate parameter, λ. In section 1.6, we present a calculation for the atrial wall thickness for each of the three datasets. We also provide a formal definition of our alternative measure, the occupied voxel fraction (OVF) and compare these values to the thickness of each dataset. In section 1.7 we demonstrate that the degree of fibre alignment in the underlying fibre maps is accurately captured by the spatial networks by comparing the fibre orientation correlation in each dataset (FOC) to the degree of longitudinal coupling in each spatial network, the longitudinal connection fraction (LCF). These results are supported by section 1.8, where we outline a simple analytical calculation for the anisotropy between the number of longitudinal and transverse connections, and compare these to values in (Spach et al. (1988)). Finally, in section 1.9, we provide an additional discussion of the technical limitations of our spatial network method, supplementing the limitations section in the main manuscript.

Section 2 provides additional results to accompany those in the main paper. In section 2.1 we plot the spatial distribution of the micro-reentrant substrate at low, medium and high risk for the two sheep atrial datasets, and discuss the influence of fibre structure in the sheep hearts. In section 2.2 we comment on the difference in results between the synthetic human data and the anatomically derived sheep data. Finally, in section 2.3 we provide a statistical analysis which quantifies the role of the wall thickness and fibre alignment in determining the micro-reentrant substrate at different risk levels in our spatial networks.

1 SUPPLEMENTARY METHODS

1.1 Atrial fibre datasets

1.1.1 Synthetically generated human fibre maps

The human dataset is detailed in (Zhao et al. (2012b)) based on techniques developed in (Krueger et al. (2011)). The atrial geometry used provides no information as to the atrial fibre structure. Therefore, a synthetic fibre map is constructed using a semi-automated rule-based approach.

From a set of 22 user-selected seed points, auxiliary lines and landmarks are drawn using a modified version of the 3d fast marching algorithm (Bærentzen (2001)). This method constructs fibre bundles by following the shortest path between seed points. Specific fibre structures which do not follow a clear shortest path between seed points, such as the crista terminalis and the pulmonary veins, are accounted for by the inclusion of additional auxiliary lines which form corridors for the fibre bundles along the anatomical feature in question. The inclusion of these auxiliary lines is important since they provide necessary boundary conditions for the fast marching algorithm, see (Krueger et al. (2011)) for details.

The method successfully reproduces key fibre bundles; validating the approach, the authors in (Krueger et al. (2011)) found that the principal fibre directions showed good correspondence with data and reproduced realistic activation time patterns when the fibre map was used in a standard electrophysiological model. However, a number of artefacts remain at the micro-level. In particular, discretisation effects are found at the tips of the atrial appendages, and the method cannot account for patient specific heterogeneity in the local fibre architecture. The lack of local heterogeneity is likely of particular importance in regions such as the atrial appendages which are known to exhibit disorganised fibre structure in some patients (Ho and Sanchez-Quintana (2009)).

Although the lack of local heterogeneity cannot be corrected for with this method, discretisation errors are prevented by averaging the local fibre orientation vectors over the five preceding and successive vectors in a given bundle. However, there are a number of small structural holes at various points in the atria which remain open. The resulting fibre map is significantly smoother than the anatomically derived fibre maps.

1.1.2 Anatomically derived sheep fibre maps

Two anatomically derived sheep atrial datasets are tested using our spatial network method; one healthy sheep acquired in (Zhao et al. (2012a)) and a sheep with pacing-induced heart failure (Thomas (2020)). These datasets are individual-specific, retaining significant local heterogeneity in the fibre maps, and are provided at high resolution, in contrast to the synthetic human fibre map. In this paper, our aim is not to assess the differences in micro-anatomical reentry between humans and sheep, or between healthy and heart failure (heart failure) sheep, but to understand how different sources of fibre orientation information effect our results.

The two sheep atrial models were acquired ex-vivo by perfusing the atria at physiologically realistic filling conditions and sealing the atria in parafin wax. The atria were then sliced using an ultramiller in $50\mu m$ ($25\mu m$) steps, and imaged at $8.33\mu m$ ($6.25\mu m$) pixel resolution for the healthy (HF) sheep. Individual images were pre-processed using a suite of techniques to minimise the presence of artefacts. In particular, and in contrast to the human dataset, small holes in the 3d atrial structure were filled and islands of isolated cells were removed from the model. A Gaussian convolution was applied to each image before extracting fibre orientation data. From each image-based model, fibre orientations are estimated from eigen-analysis of the structure tensor, where the principal fibre direction is taken as the eigenvector with the smallest

eigenvalue. A second phase of smoothing was applied to the model after estimating the fibre vectors. Although the exact level of smoothing is not specified in (Zhao et al. (2012a)), local variation in fibre orientation is significantly higher than for the synthetic human datasets.

To validate the structure tensor approach, the authors of (Zhao et al. (2012a)) and (Thomas (2020)) systematically checked the calculated fibre orientations against high resolution images of the individually imaged slices where the orientation of individual cardiomyoctes can be seen. Testing the fibre maps using a simple electrophysiology model, the authors find preferential conduction along key fibre bundles including the crista terminalis, pectinate muscles and the Bachmann bundle.

1.2 Fibre tractography

Each atrial dataset provides a 3D vector field representing the best estimate for the local fibre orientation at each point in the 3D image of the atria. However, there is no information in the datasets regarding fibre connections. To apply our spatial network methods, we generate fibre tracts across the atrial structure from the underlying local fibre orientations. For a summary of standard fibre tractography approaches, principally pioneered in neuroscience, see (Caan (2016)).

In developing our tractography procedure, we experimented with two existing algorithms: Fibre Assignment by Continuous Tracking (FACT) and Evenly Spaced Streamlines (ESS). FACT (Mori et al. (1999)) is often used for modelling purposes, whereas the ESS algorithm was developed in neuroscience for visualisation purposes. The principal differences between the methods are described in Table (S1). The qualitative differences between fibre maps generated using FACT and ESS are shown in Fig. S1 for the healthy sheep atria. The principal limitation of each method is that in FACT, arbitrary fluctuations in density of fibres existed (shown in Fig. S2), and in ESS the mean fibre length was far shorter than that in FACT. This motivated the use of an adapted version of ESS that prioritised the generation of long fibres over short fibres. Given that no density information is supplied in the atrial datasets, an algorithm that favoured a uniform distribution of fibre density was preferable.

Table S1. The key differences between the fibre tractography approaches FACT and ESS. Generation of a fibre in ESS had an extra termination condition compared to FACT.

	ESS	FACT
Tracking Method	Step along local orientation with fixed step size d_{step}	Follow local orientation until a voxel boundary is reached
Density Regulation	Generation of a fibre terminated if closer than d_{sep} to a different fibre	Global cost function is applied after fibre tracking to selectively remove fibres
Limitation	Fibres generated were short	Arbitrary density fluctuations existed in the fibre map

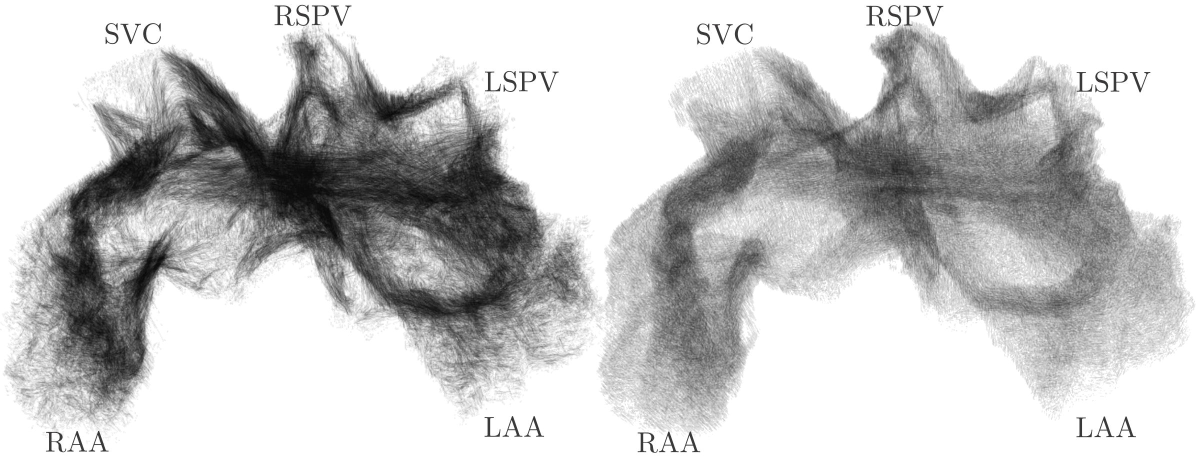

Figure S1. Visualised fibre maps generated from the healthy sheep atria using FACT (left) and ESS (right), shown from the anterior view. Qualitatively, FACT resulted in arbitrarily patchy regions, whereas ESS had a uniform coverage of short fibres. Our adapted tractography approach addresses both of these limitations.

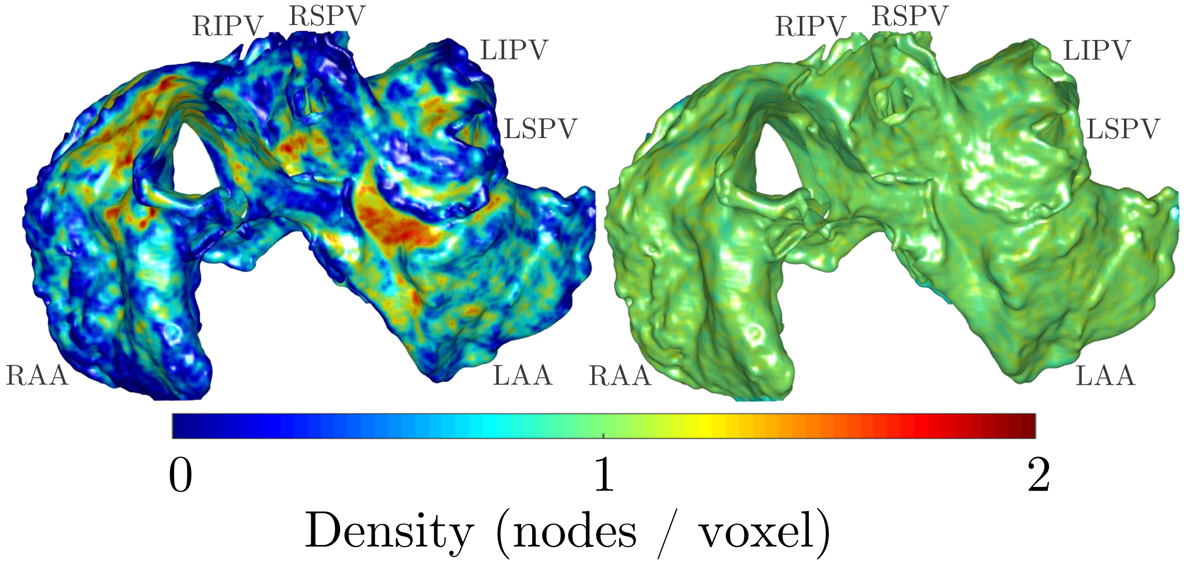

Figure S2. Heatmaps of node density, from networks generated on the healthy sheep atria using FACT (left) and ESS (right), shown from the superior view. The density regulation following FACT proved mostly ineffective, creating arbitrary regions of under/overdensity. ESS, by comparison, produced a network of near uniform density.

By modifying the ESS algorithm, the fibre maps generated were of approximately uniform density and fibre length was maximised. This modified approach is as follows:

1. Seed points (an equal number to the number of occupied voxels) are generated randomly across the atrial structure in voxels not containing any existing fibre vertices.

2. Fibres are generated from seed points by Euler stepping along the local voxel orientation with fixed step size $d_{step} = 1$ in both the forwards and backwards directions.
3. For each fibre being propagated in the forward and backward direction, the fibre is terminated if, (a) the edge of the dataset is reached, (b) an empty voxel is reached, (c) the difference in angle between the current and next voxel orientation exceeds a threshold of $45°$, see (Sun et al. (2019)), (d) the distance between the new vertex and any vertex on a different fibre is less than $d_{sep} = 0.7$ (Merhof et al. (2005)).
4. Once a fibre has terminated, it is added to the final fibre map if its length exceeds a threshold l, otherwise the fibre is discarded.
5. If all seed points have been checked and no new fibres exceeding the threshold length l can be added to the fibre map, steps 1-4 are repeated with a new threshold. In order, the thresholds used in each cycle are $l \in [30, 25, 20, 15, 10, 5, 0]$. This is a novel approach to fibre generation and aims to maximise atrial coverage while also ensuring that the fibres generated are as long as possible. The l values were chosen by trial and error, with the maximum length corresponding approximately to the longest fibres we observe using traditional tractography methods such as FACT.
6. Once a threshold of $l = 0$ has been reached, steps 1-4 are repeated until vertices placed on existing fibres are present in at least 99% of the voxels occupied by tissue in each dataset.

Some applications in computational cardiology, which implement a fibre structure in the atria for modelling purposes, explicitly build in specific known fibre bundles which are important for electrical propagation in the atria (Ho et al. (2002)). For our application, we do not give any special treatment to specific fibre bundles, relying solely on the local fibre orientation data in each dataset. However, we note that the importance of certain fibre bundles is preserved in local data as noted in (Zhao et al. (2012a)).

1.3 Spatial network coupling parameters

1.3.1 Spatial networks with fibre structure

For each dataset, spatial networks were generated with five values of the characteristic distance, c. The specific values used and corresponding risk parameters, λ, are given in Table S2. For a precise definition of the risk parameter, λ, see section 1.5. Specific parameter choices are labelled as having low, medium or high risk of micro-reentry. These are qualitative markers and are introduced to simplify comparison. The low risk parameter values are chosen by finding the largest characteristic coupling distance for which the risk of micro-reentry is non-negligible; here the rate of micro-reentry, λ, is very low. Different values of the characteristic coupling used across the three datasets reflects differences in the largest characteristic coupling at which micro-reentry is detected. Reductions in the characteristic coupling distance increase the risk of micro-reentry, with the medium risk label being assigned to intermediate parameter values where the rate of micro-reentry is moderate, and the high risk label being assigned to parameter values where micro-reentry is detected almost immediately after initiating a simulation.

	**Human**				
**Characteristic Distance,** c	0.5250 (L)	0.5000	0.4750 (M)	0.4500	0.4250 (H)
**Risk Parameter,** λ	0.01569 (L)	0.0190	0.0294 (M)	0.0689	0.2456 (H)

	**Sheep: Healthy**				
**Characteristic Distance,** c	0.5500 (L)	0.5250	0.5000	0.4750 (M)	0.4625 (H)
**Risk Parameter,** λ	0.01900 (L)	0.0250	0.0292	0.0600 (M)	0.1464 (H)

	**Sheep: Heart failure**				
**Characteristic Distance,** c	0.5250 (L)	0.5000	0.4750 (M)	0.4625	0.4500 (H)
**Risk Parameter,** λ	0.0082 (L)	0.0138	0.0410 (M)	0.0754	0.1881 (H)

Table S2. Fibre model characteristic distance values, c, and corresponding risk parameter, λ, for each atrial dataset. The labels (L), (M) and (H) indicate the parameter values corresponding to the low, medium and high rate of micro-reentry (risk) spatial networks referred to throughout the text.

1.3.2 Spatial networks excluding fibre structure

For each atrial dataset, we generate null model spatial networks, excluding the underlying fibre structure, for three values of the characteristic coupling, corresponding to high, medium and low risk cases, see Table S3. The characteristic coupling values in Table S3 are significantly larger than the coupling values for the fibre model, see Table S2, since there are no permanent longitudinal edges.

	Human		
**Characteristic Distance, c**	0.6500 (L)	0.6375 (M)	0.6250 (H)
**Risk Parameter, λ**	0.0240 (L)	0.0527 (M)	0.2775 (H)

	Healthy Sheep		
**Characteristic Distance, c**	0.6375 (L)	0.6250 (M)	0.6125 (H)
**Risk Parameter, λ**	0.0141 (L)	0.0310 (M)	0.1534 (H)

	HF Sheep		
**Characteristic Distance, c**	0.6500 (L)	0.6250 (M)	0.6175 (H)
**Risk Parameter, λ**	0.0161 (L)	0.0520 (M)	0.2152 (H)

Table S3. Fibre-less null model characteristic distance values, c, and corresponding risk parameter, λ, for each atrial dataset. The labels (L), (M) and (H) indicate the parameter values corresponding to the low, medium and high rate of micro-reentry (risk) spatial networks referred to throughout the text.

.

1.4 Discrete Diffusion Model

To identify the locations in the spatial network capable of forming micro-anatomical reentrant circuits, we search the network structure for an isolated fibre tract, defined as a continuous string of transversely disconnected nodes, such that a non-backtracking path from the start of the fibre tract, through the fibre tract, and back around the fibre tract exists and is at least length τ, where τ is the refractory wavelength.

Computationally, such locations can easily be identified by applying a discrete diffusion model to the network. The approach is based on previous simple models of micro-anatomical reentry in (Falkenberg et al. (2019); Christensen et al. (2015)). For simulation purposes we set $\tau = 83$ such that the threshold length for a micro-reentrant circuit is marginally smaller than the circuits identified in (Hansen et al. (2015)) [1]. Note, however, that this does not exclude circuits forming that are significantly larger than the threshold, $\ell \gg \tau$, only preventing excessively small circuits of length $\ell < \tau$. The specific value of τ is largely irrelevant since increases (decreases) in τ can be counteracted by an appropriate decrease (increase) in the characteristic coupling c (Ciacci et al. (2020)).

First, we define a point in the right atrium as the sinus node, around which all nodes within an arbitrary radius 2 are automatically excited every $T = 1000$ time steps. All nodes which are in the excited state at time t_i enter the refractory state at $t_i + 1$, during which nodes cannot be re-excited, and remain in this state for τ time steps. At time $t_i + \tau + 1$, these nodes enter the resting state. Nodes in the resting state at time t_i enter the excited state if (1) at $t_i + 1$ they are directly connected to a node which is excited at time t_i, and (2), conduction is not blocked. Nodes which should enter the excited state at time $t_i + 1$ may fail to

[1] Starting with voxels with $100\mu m$ spacing, a value of $\tau = 250$ gives a minimum refractory wavelength of 25mm. To ensure a fixed refractory wavelength, τ must be divided by a factor of 3, $\tau = 250/3 \approx 83$, when the vector field is down-resolved to $300\mu m$ resolution.

do so with small probability $\epsilon = 0.0025$. This simulates potential unidirectional conduction block at the micro-level in a simple and efficient way, while avoiding extensive wavefront breakup (i.e. wavefronts should remain approximately planar as is expected during sinus rhythm). The specific value of ϵ is arbitrary, but must be sufficiently small such that in any given activation cycle, the probability of a micro-anatomical reentrant circuit forming is small (Ciacci et al. (2020)).

A micro-reentrant circuit is identified by allowing the excited wavefront to diffuse through the spatial network from the sinus node, and observing where isolated stochastic conduction blocks result in the formation of a circuit, shown schematically in Fig. S3. This emerges naturally in the DDM, and is identified by locating the first node which activates multiple times within a single sinus activation cycle of length T. We have visually verified that the location identified computationally corresponds to the location in which we observe rapidly firing micro-reentrant triggers in the simulations.

As a result of the stochastic nature of conduction block, a potential reentrant circuit only has a small probability of triggering with each sinus wavefront which crosses it. Therefore, for simulation purposes we run the discrete diffusion model for 10^5 time steps to allow sufficient time for a reentrant circuit to activate if present. Once a circuit is identified, the simulation is terminated. In the case where multiple circuits coexist within a single spatial network, the first to activate is recorded, although this is largely mitigated by an appropriately small choice of ϵ. By repeating this process until at least 10^3 circuits have been identified for each fibre map and each value of the characteristic coupling distance c, we can construct a probabilistic risk map for the micro-reentrant substrate across the atria.

To ensure robustness to different randomly generated atrial micro-structures, after each micro-reentrant circuit is identified, we return to the uncoupled spatial network shown in Fig. 3(C) of the main paper and generate new transverse connections using Eq. (1) in the main paper. By averaging over a large set of diverse atrial micro-structures, our final results give a robust probabilistic overview of where in the atrial structure micro-reentrant circuits emerge at different levels of coupling.

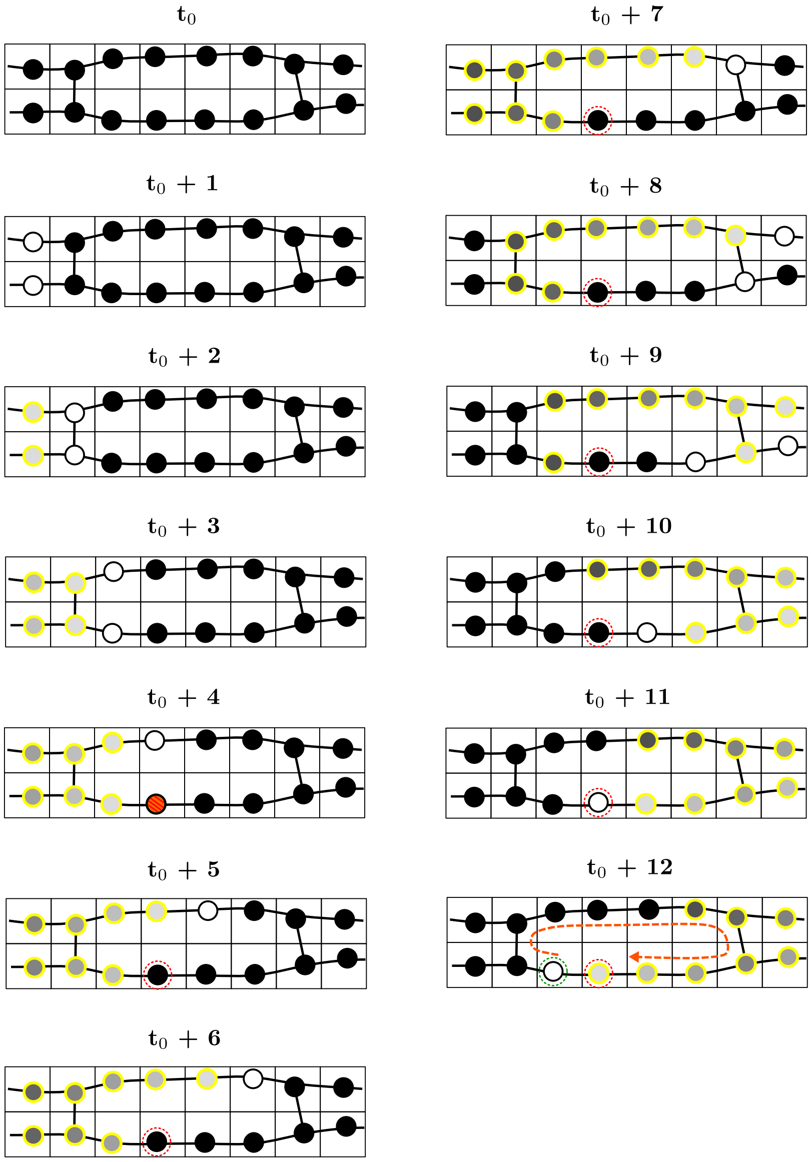

Figure S3. A schematic illustrating the mechanism by which a micro-reentrant substrate is detected by the discrete diffusion model. A dormant structure is present in the spatial network at time t_0 (black circles). A wavefront enters from left to right (white cells). Excited cells remain refractory for τ timesteps after excitation (greyscale with yellow border). The wavefront is spontaneously blocked in the lower fibre at $t_0 + 4$ (orange-red checked circle). The wavefront proceeds along the top fibre and reenters the lower fibre at time $t_0 + 8$. If the closed loop is sufficiently long to form a micro-reentrant circuit, at time $t_0 + 12$, the node to the left of the previously blocked node will excite (circled in green). Otherwise, if the loop is too short, the node will be in the refractory state and will not excite. The DDM detects the repeated activation of the node circled in green as the location of a micro-reentrant substrate.

1.5 Calculating the risk of micro-anatomical reentry

We would like to derive a measure of the risk of micro-reentry for each spatial network. Ideally, we would count the absolute number of loops in each network which are of length $\ell \geq \tau$, but for computational reasons related to the NP-hardness of finding path lengths in networks, this is not practically feasible. As an alternative, we return to the DDM and note that all nodes in the network are equally susceptible to conduction block. If each structure has a similar number of nodes which, if blocked, may initiate a micro-anatomical reentry, then the probability of initiating any given micro-reentrant circuit is approximately constant. Hence, for a spatial network with N potential substrates, the rate, λ, at which new micro-reentries are initiated in the DDM is directly related to the number of substrates N. This argument follows directly from similar arguments in (Ciacci et al. (2020)).

A process where events take place independently at a constant rate λ is known to follow a Poisson point process.[2] Hence, from the start of a DDM simulation, the time until the first micro-anatomical substrate is identified follows an exponential decay distribution, $\rho(t) \propto e^{-\lambda t}$. The distributions of the time taken to identify a micro-anatomical substrate using the DDM are shown in Figs. S4 and S5. Fitting these distributions on a log-linear scale lets us extract the rate parameter λ, which varies depending on the choice of characteristic coupling distance, c, in each spatial network. The rate λ is directly related to the number of potential micro-reentrant substrates in each spatial network. Therefore, λ can be used as a measure for the risk of micro-reentry in our framework.

The activation rate λ measures the risk of micro-reentry for each spatial network as a whole. In a local area, we consider a specific voxel, v, and calculate the number of circuits which form in that particular voxel of the DDM, N_v, when sampling a fixed number of identified circuits, N. The rescaled rate of reentry for the voxel v is then given by

$$\tilde{R_v} = \frac{\lambda \cdot N_v}{\sum_v N_v}, \tag{S1}$$

where the denominator provides a normalisation. When plotting the rescaled rate of reentry onto the atrial surfaces, we smooth $\tilde{R_v}$ using a 3d Gaussian convolution with a standard deviation of 5 voxels ($\sim 1.5mm$).

1.5.1 Time to reentry distributions

Figure S4 shows the distribution for the time needed in the discrete diffusion model for a micro-reentrant circuit to activate in the spatial networks including fibre structure with different values of the characteristic coupling distance, c. The rate at which circuits form, λ, is fit to each distribution on a log-linear scale. A shallow gradient (small λ) indicates that there are very few regions in the fibre map which act as a substrate for micro-reentry (low risk), whereas a steep gradient (large λ) indicates that there are many distinct regions which may act as a substrate for reentry (high risk). Our results indicate that the network representation of the atria undergoes a transition from a state where there are no regions susceptible to micro-reentry at low levels of structural remodelling, to a state where a rapidly increasing number of independent regions are a potential substrate for micro-reentry as the network undergoes moderate to severe structural remodelling, simulated by lowering the coupling parameter, c.

Figure S5 shows the probability density for the time to reentry for the three null models where the underlying atrial fibre structure is neglected.

[2] It is interesting to note that the Poisson point process has recently been used to explain the generation and destruction of phase singularities during fibrillation, see (Dharmaprani et al. (2019, 2021)).

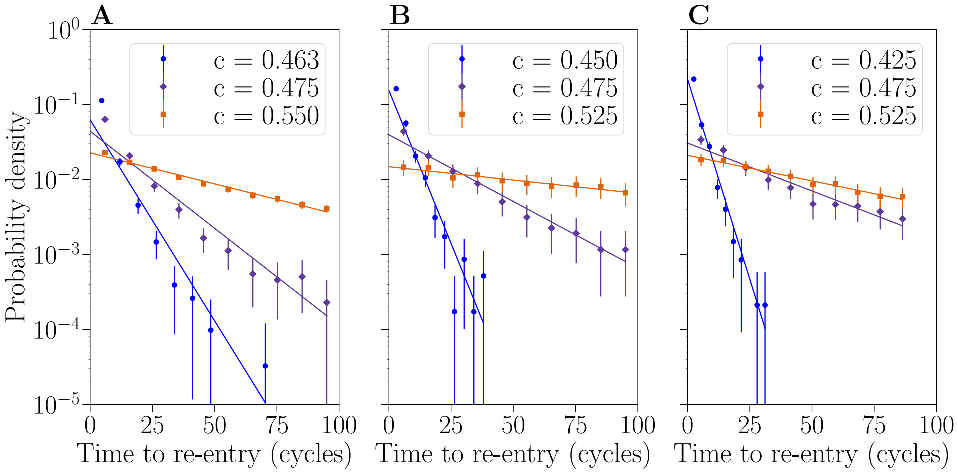

Figure S4. The time to reentry distribution, in units of the cycle length T, for different values of the characteristic coupling, c. (a) The healthy sheep atria, (b) the heart failure sheep atria, and (c) the human atria. Each probability distribution is fit to an exponential decay curve $\propto e^{-\lambda t}$, indicated by the solid lines, where the magnitude of λ corresponds to the gradient of each fit on a log-linear scale.

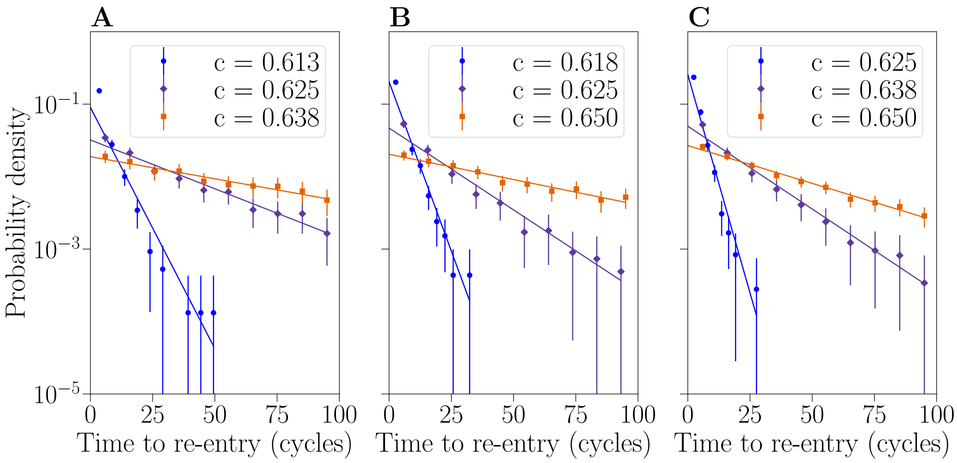

Figure S5. The time to reentry distribution in the fibre-less null model, in units of the cycle length T, for different values of the characteristic coupling, c. (a) The healthy sheep atria, (b) the heart failure sheep atria, and (c) the human atria. Each probability distribution is fit to an exponential decay curve $\propto e^{-\lambda t}$, indicated by the solid lines. For all three atrial datasets, λ is largest at low spatial coupling (small c) suggesting a high risk for reentry, and λ is smallest at high spatial coupling, suggesting that reentry is rare. For values of the characteristic distance much larger than the values shown, the risk of reentry is effectively zero within the simulation timescale.

1.6 Atrial Wall Thickness & Occupied Voxel Fraction

In this section we calculate the atrial wall thickness and the corresponding occupied voxel fraction (OVF) values. The two distributions are not easy to compare since thickness is not defined in the bulk using our method. However, we illustrate that the OVF naturally increases in thicker regions of the atria, and that the two measures behave differently in regions of high wall curvature.

1.6.1 Calculating Atrial Thickness

Tissue thickness is a key factor implicated in studies investigating the risk of re-entry both experimentally and computationally (Zhao et al. (2017); Alonso et al. (2016)). Calculating the thickness of such a topology is non-trivial. Naive methods, such as following the surface normal into the volume until the outside of the mesh is reached results in large inaccuracies. As an alternative, we follow the approach of (Wang et al. (2019)) and solve the 3D Laplace equation with special epi-endocardial boundary conditions, then measure the distance travelled following the gradient $\nabla\phi$ of the Laplace solution ϕ between the epi- and endocardium. This required that the epi-endocardial surfaces be defined. First, the holes in each atrial dataset were manually sealed. Surface voxels were defined as any full voxel with an adjacent empty voxel. By placing three anchor nodes, one outside the atrial mesh, and two inside the atrial mesh, one in each of the left and right atrium, the inside and outside atrial surfaces were defined based on which anchor node a surface voxel could connect to through a continuous path without passing through any occupied voxels of the atrial dataset. Surface voxels which were unable to connect to any anchor node correspond to internal voids, although we note that these are generally very rare. After defining the internal and external atrial surfaces, previously sealed structural holes are reopened. A visual representation of the internal and external surfaces of the healthy sheep atria is shown in Fig. (S6).

The 3D Laplace equation is given by

$$\nabla^2\phi = 0. \tag{S2}$$

Equation (S2) was solved on a uniform cartesian grid (x_i, y_j, z_k) of grid spacing h over the region $x \in [0, L_x]$, $y \in [0, L_y]$, $z \in [0, L_z]$ where

$$x_i = ih, \qquad i = 0, 1, 2, ..., p, \qquad p = \frac{L_x}{h}, \tag{S3}$$

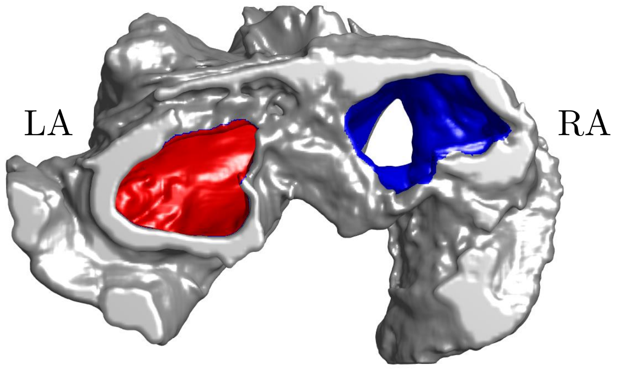

Figure S6. The definition of the atrial surfaces for the healthy sheep. Epicardium in grey. Left endocardium in red. Right endocardium in blue.

$$y_j = jh, \qquad j = 0, 1, 2, ..., q, \qquad q = \frac{L_y}{h}, \tag{S4}$$

$$z_k = kh, \qquad k = 0, 1, 2, ..., r, \qquad r = \frac{L_z}{h}. \tag{S5}$$

Equation (S2) was discretised using a central difference method. The discrete second derivative in x is given by

$$\frac{\partial^2 \phi}{\partial x^2} = \frac{\phi_{i+1,j,k} + \phi_{i-1,j,k} - 2\phi_{i,j,k}}{h^2} \tag{S6}$$

and the second derivatives in y and z are analogous. The discretised 3D Laplace equation is therefore given by

$$\frac{\phi_{i+1,j,k} + \phi_{i-1,j,k} + \phi_{i,j+1,k} + \phi_{i,j-1,k} + \phi_{i,j,k+1} + \phi_{i,j,k-1} - 6\phi_{i,j,k}}{h^2} = 0. \tag{S7}$$

Equation (S7) was solved iteratively for ϕ using successive over-relaxation

$$\phi^l_{i,j,k} = (1-\omega)\phi^{l-1}_{i,j,k} + \omega\phi_{i,j,k} \tag{S8}$$

where l denotes iteration number and $\omega = 1.4$ is a recommended solving parameter (Wang et al. (2019)). A cross-section of the Laplace solution is shown in Fig. (S7) for the healthy sheep atrium. Distance travelled along $\nabla\phi$ between the epi-endocardial surfaces was used as the measure of atrial thickness, rescaled such that the units are given in millimetres.

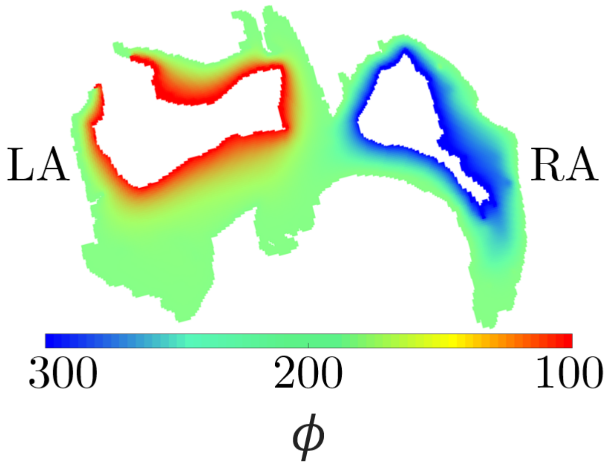

Figure S7. A cross-section of the healthy sheep atria showing the Laplace solution across the atrial structure with the left atrial endocardium (red), right atrial endocardium (blue) and the epicardium (green).

1.6.2 Calculating the occupied voxel fraction

The occupied voxel fraction (OVF) is defined as the number of voxels within a radius $\tilde{r} = 5$ (approximately 1.5mm) which are inside the atrial structure, normalised by the total number of voxels within a sphere of radius $\tilde{r}$. See Fig. S8 for a schematic describing the calculation. The OVF is not normalised by wall thickness, capturing the qualitative properties of traditional wall thickness measurements implicitly. The OVF increases (decreases) if the atrial walls become thicker (thinner), and distinguishes between regions with convex, flat, or concave wall morphologies. A natural consequence of this method is that branchy regions of the atrial myocardium, such as the pectinate muscles, are identified as having a low OVF, whereas voxels in the atrial bulk which are more than 1.5mm from the surfaces will have an OVF of 1. This is not problematic for our purposes where we find that bulk voxels in thick atrial regions almost never harbour micro-anatomical reentry. For general purposes, the OVF radius can be adjusted as required.

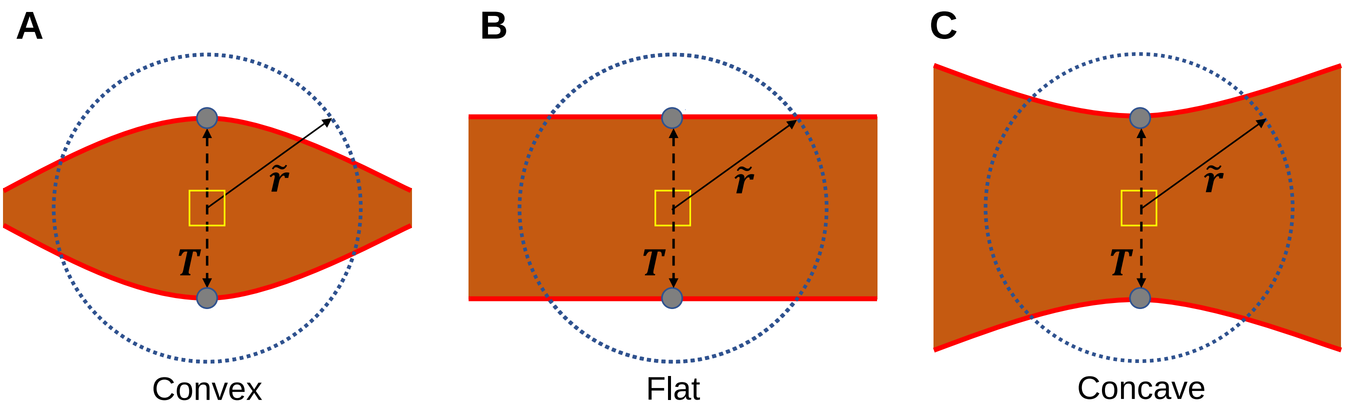

Figure S8. A 2d schematic illustrating the definition of the occupied voxel fraction (OVF); an analogue to wall thickness which is well defined in the atrial bulk. Consider three regions with equal thickness T. If we measure the fraction of myocardial voxels (brown region) enclosed within a sphere of radius $\tilde{r}$ (dotted blue circle), relative to the volume of the sphere, we note that the occupied voxel fraction is lowest for convex wall morphologies, moderate for flat morphologies, and largest if the walls are concave. Increasing (decreasing) the thickness T typically increases (decreases) the corresponding OVF values.

Qualitatively comparing the OVF and thickness, we note that the OVF appears smoother than the thickness measurement, and is subject to fewer anomalous outlying values (such as very large thickness measurements at the end of a vessel opening). The OVF is mathematically simple and less computationally expensive than traditional thickness measurements.

1.6.3 Calculated OVF and Thickness

Figure S9 shows the atrial wall thickness values for the three atrial datasets. For comparative purposes, all three datasets are plotted using the same colourbar. Regions with a thickness in excess of 4mm are shown in grey to ensure high fidelity in the visualisation of thinner atrial regions. Figure S10 shows the OVF calculations for the three atrial datasets. Visual inspection shows that thickness values fluctuate over a much larger range than the OVF calculations which appear smoother.

Figure S11 shows the OVF for an atrial cross section in each of the three atrial datasets. The figure demonstrates that the largest OVF values are found in the atrial bulk, and that the OVF naturally increased (decreases) with increasing (decreasing) wall thickness. For voxels further than 1.5 mm away from the atrial

surfaces, the OVF is measured as 1. This demonstrates that the OVF captures the qualitative properties of traditional thickness measurements and extends naturally to the atrial bulk.

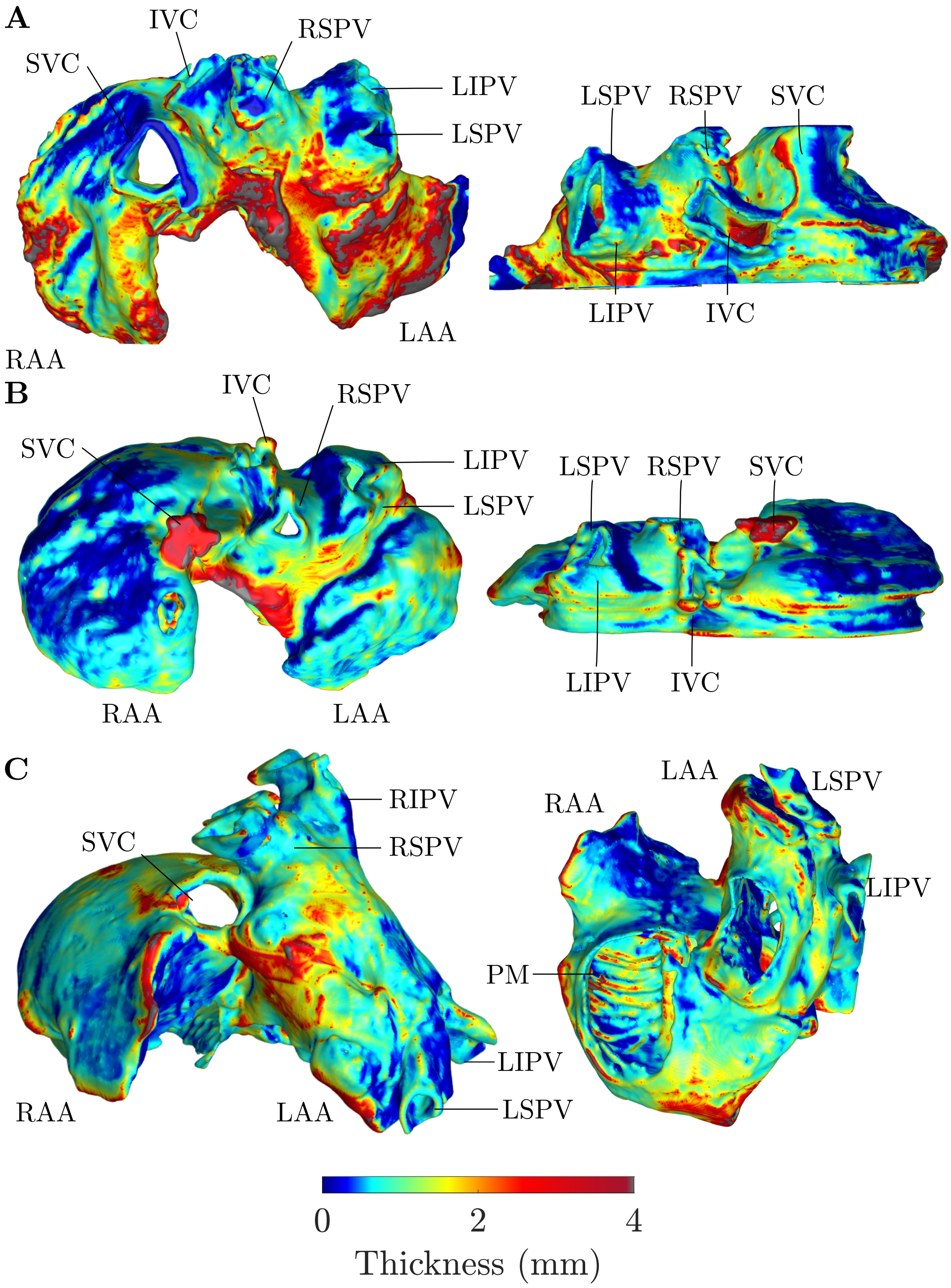

Figure S9. The thickness of the atrial wall for (a) the healthy sheep atria, (b) the heart failure sheep atria, and (c) the human atria. The colour scale is capped at 4mm with thicker regions shown in grey. Regions of very high curvature in some cases appear as points with very large wall thickness.

Apart from issues with defining thickness in the bulk, thickness measurements show small inconsistencies in regions of high curvature such as the pectinate muscles or vessel openings, see Fig. S12. These have

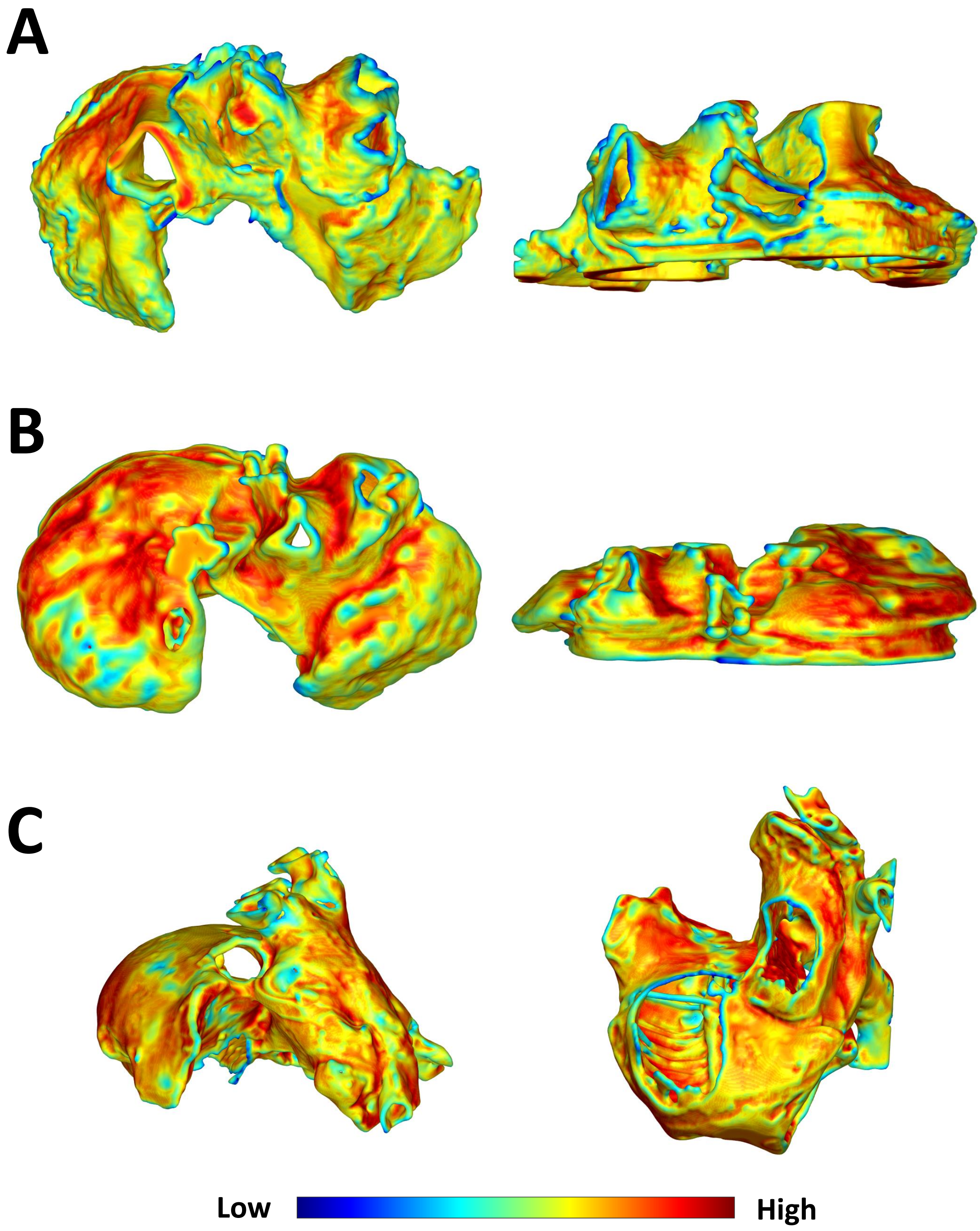

Figure S10. The OVF for (a) the healthy sheep atria, (b) the heart failure sheep atria, and (c) the human atria. Regions with the highest OVF values are not visible since they are within the atrial bulk.

low OVF but a large thickness, since the thickness measurement attempts to calculate the path from endocardium to epicardium. If using the thickness to quantify regions of micro-reentrant risk, spatially isolated fibres would counter-intuitively be identified as regions of high thickness. Using the OVF as an alternative measure avoids this issue.

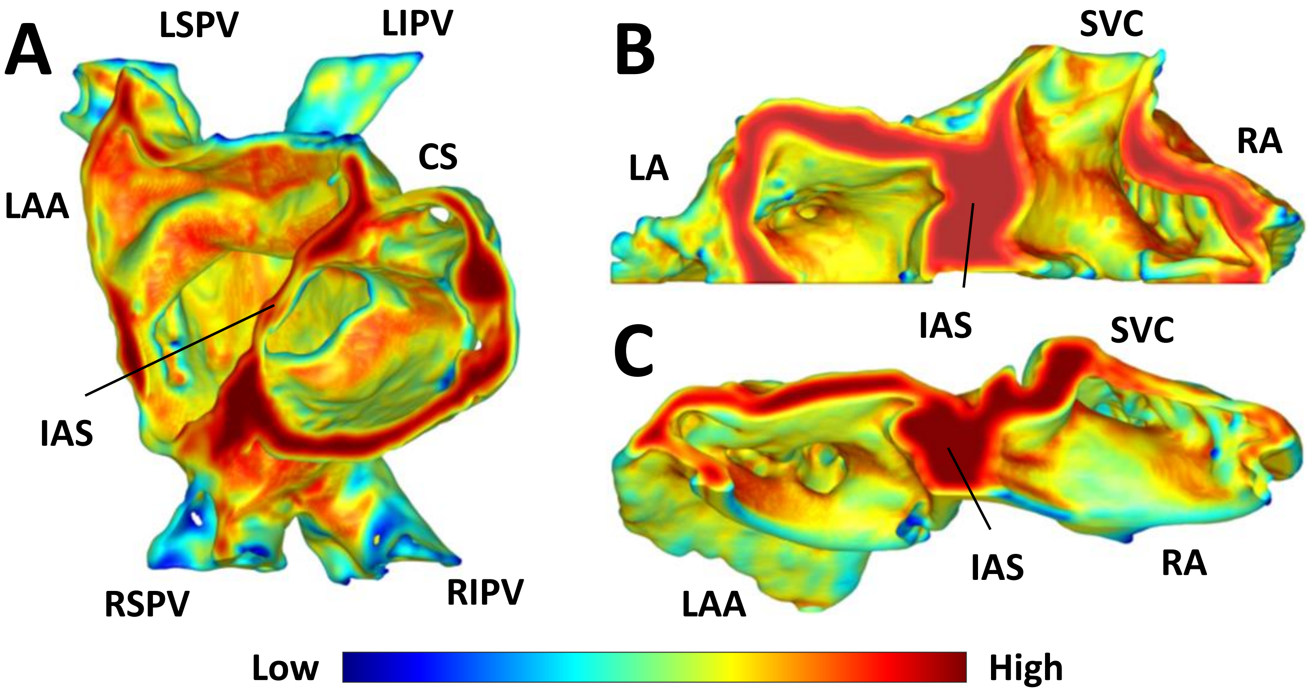

Figure S11. Three atrial cross sections showing the occupied voxel fraction (OVF) in the (A) human atria, (B) healthy sheep atria, and (C) the HF sheep atria. The figure shows that the largest OVF values are found in the atrial bulk, with lower values on the atrial surfaces. Bulk voxels more than 1.5 mm from the surfaces all have an OVF of 1. Regions of the atria which are thin (e.g. LAA in human atria) have a lower OVF than thick regions (e.g. IAS in sheep atria).

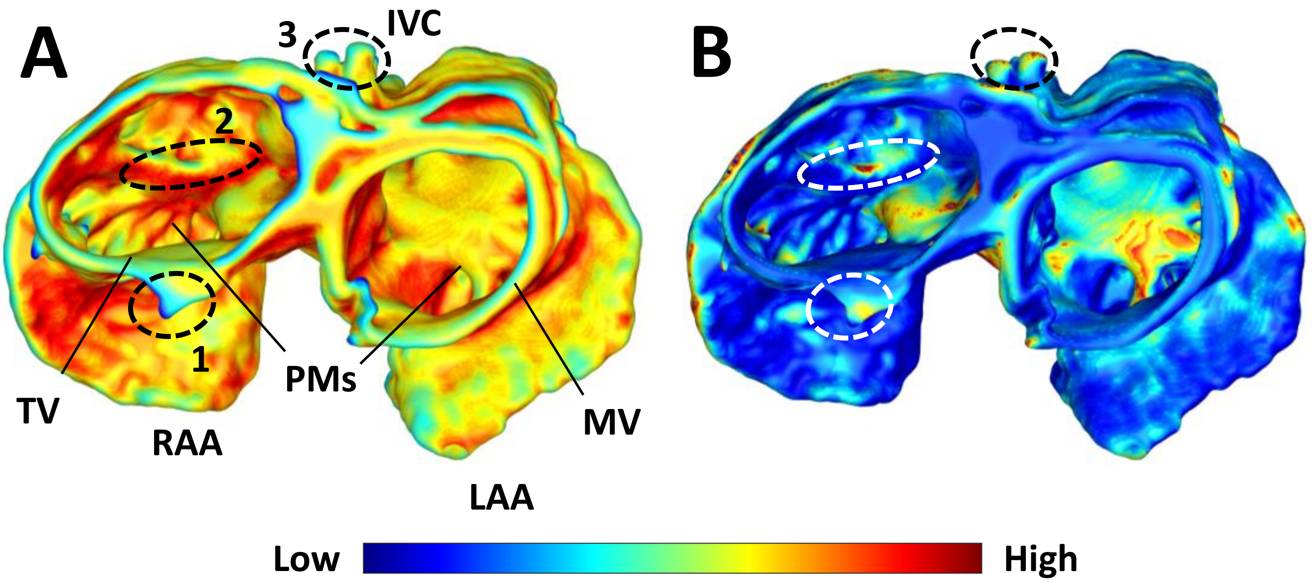

Figure S12. The (A) OVF and (B) wall thickness calculated in the heart failure sheep, shown from the inferior view. Regions which are spatially isolated and have significant wall curvature (regions 1–3), naturally appears as having low OVF. However, the wall thickness implementation results in these regions being measured as having a high thickness. This is particularly important along the PMs, at the TV and MV openings, and at the end of the IVC and PVs.

1.7 Validating spatial network fibre structure

1.7.1 Defining fibre orientation correlation (FOC) and the longitudinal connection fraction (LCF)

In the current paper, we have focused exclusively on generating spatial networks from pre-existing image based models of the atria which include fibre orientation data. Therefore, we must treat the pre-existing fibre datasets as a ground truth and validate that our spatial network methods accurately capture the properties of the underlying fibre dataset. The individual datasets have been validated previously, see section 1.1. However, in the future it will be important to validate the full spatial network pipeline, starting with the initial acquisition of each image-based model and fibre structure, and ending with a comparison between model predictions and the experimentally observed risk substrate.

Each fibre map is defined by a vector field, where the vector at each coordinate corresponds to the estimated fibre direction in that specific voxel. We can measure the local heterogeneity in the fibre map by calculating the fibre orientation correlation (FOC), defined as the average value of the absolute dot product of each voxel's fibre orientation vector with the fibre orientation vector of the voxel's six immediate neighbours (on a cubic mesh). Neighbours outside the atrial geometry are excluded from the calculation. Taking the absolute value ensures that the FOC does not distinguish between parallel and anti-parallel vectors. Each of the vectors is normalised such that the FOC can take a minimum value of 0, corresponding to a vector which is orthogonal to all of its neighbours, and a maximum value of 1, where all vectors are aligned.

We would like to extract an equivalent measure from each spatial network for validation purposes. To do this, we calculate the longitudinal connection fraction (LCF), defined as the number of longitudinal edges (pink edges in Fig. 3D of main paper) attached to each node in a voxel, normalised by the total number of edges connected to each node, either longitudinal or transversal (blue edges in Fig. 3D of main paper). This measure is chosen as a proxy for fibre alignment since there is no natural concept of fibre alignment in the spatial networks. In regions with a high density of aligned fibre tracts, we expect the LCF to be large, whereas in regions with disordered fibres, we expect the LCF to be smaller.

Although geometric factors also impact the LCF, such as proximity to the atrial walls, we expect this effect to be small. If the spatial networks accurately capture the underlying heterogeneity of each fibre dataset, we expect to find a linear relation between the LCF and the FOC.

1.7.2 Comparing FOC and LCF values

Figure S13 shows the FOC and corresponding LCF values for the human and healthy sheep atria. The equivalent for the HF sheep is shown below..

The FOC demonstrates that fibre alignment is significantly stronger for the synthetic human dataset than for the anatomically derived healthy sheep. In the human atria, approximately 90% of voxels have an FOC value greater than 0.90, whereas for the healthy sheep, this is around 16%. Despite this, regional fluctuations in the FOC are observed in both cases, although the range is much larger for the sheep. The differences in the FOC ranges are almost certainly attributable to the synthetic, low resolution nature of the human fibre dataset and excessive smoothing. Regions of reduced FOC in the human are most likely artefacts of the synthetic fibre generation process and align with the boundaries of fibre bundles.

For our purposes, it is important that regional differences in the FOC are accurately captured as equivalent differences in the corresponding LCF values. Figures S13(B) and S13(E) show the LCF at low risk for the human and healthy sheep respectively from the superior view. In both cases we note that the LCF is

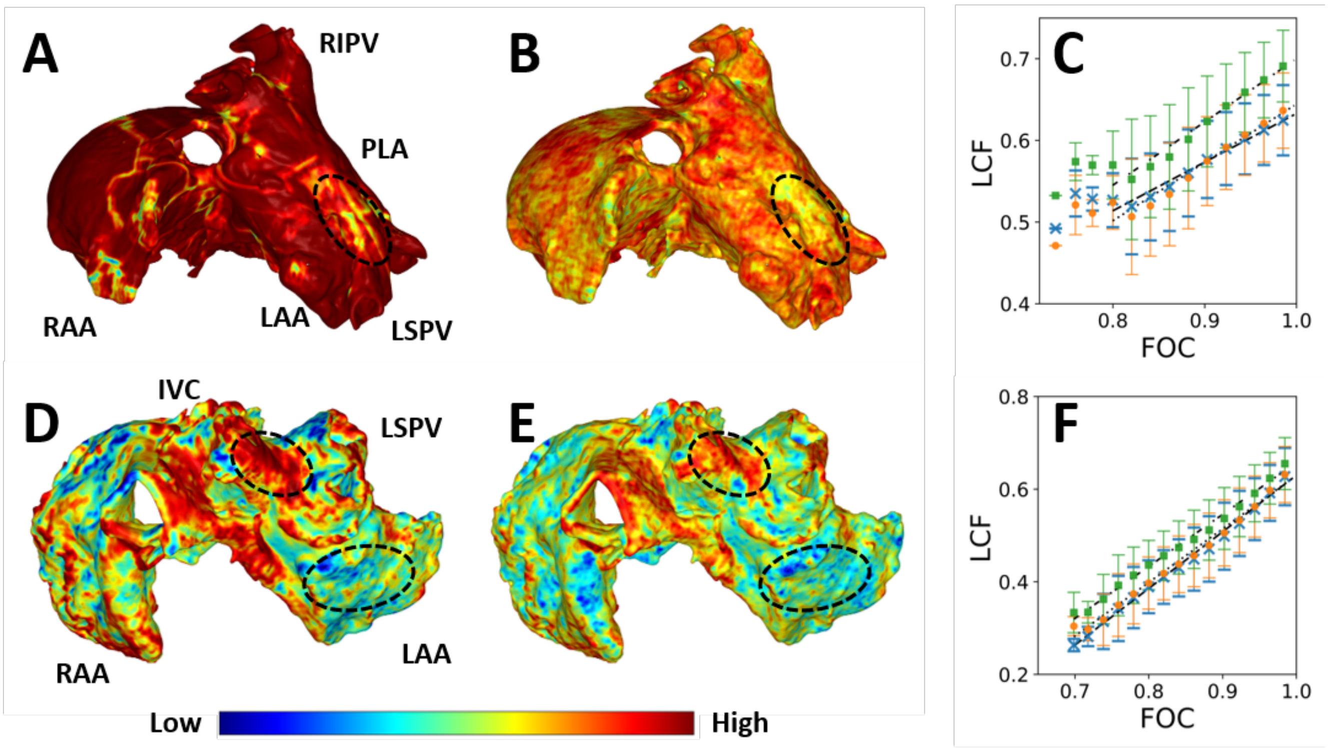

Figure S13. The fibre orientation correlation (FOC) and longitudinal connection fraction (LCF) for the human and healthy sheep atria. (A) Human FOC. (B) Human LCF for low risk spatial network. (C) Binned LCF for all voxels in the human atria against their corresponding FOC values at high (blue crosses) medium (orange points) and low risk (green squares). High, medium and low risk data are linearly fitted (dashed, dotted and dot-dashed respectively) with weighted least squares. High risk, $R^2 = 0.854$. Medium risk, $R^2 = 0.928$. Low risk, $R^2 = 0.896$. (D)–(F) Equivalent for healthy sheep atria. High risk, $R^2 = 0.993$. Medium risk, $R^2 = 0.988$. Low risk, $R^2 = 0.995$.

significantly noisier than the equivalent FOC calculation. This is to be expected since the FOC is an exact measurement derived uniquely from the underlying fibre orientation vectors, whereas the LCF will exhibit random fluctuations from the probabilistic assignment of edges in each spatial network.

For the human atria, most LCF values fall within a small range. Key regions which show reduced FOC such as the junction between the left superior pulmonary vein (LSPV) and the posterior left atrium (PLA) correspond well to regions of reduced LCF. Quantifying the correlation between FOC and LCF values, we bin voxels by their FOC value and calculate the average LCF in each bin, shown in Fig. S13(C). Fitting the data using weighted least squares, we find that strong linear correlations between LCF and FOC values for low ($R^2 = 0.896$), medium ($R^2 = 0.928$) and high risk ($R^2 = 0.854$) spatial networks. Average LCF values decrease with increasing risk as required.

For the healthy sheep atria, the correspondence between LCF and FOC values is clearer than for the human atria, likely due to the wider range of FOC values. Comparing sheep FOC and LCF values, high FOC regions such as the PLA correspond to high LCF values, and conversely, regions with low FOC such as the atrial appendages correspond to low LCF regions. Fitting the average LCF against binned FOC values shows strong linear correlations for the low ($R^2 = 0.995$), medium ($R^2 = 0.988$) and high risk ($R^2 = 0.993$) spatial networks. Comparable results are shown in SM section 1.7.3 for the HF sheep atria.

1.7.3 HF Sheep Fibre Correlation

Figure S14 shows the fibre orientation correlation (FOC) and the longitudinal connection fraction (LCF) at low risk for the HF sheep from the inferior atrial view. The patchiness of both images indicates the significant local heterogeneity in the HF sheep fibre orientations. Compared to the human and healthy sheep, the FOC and LCF values appear to fluctuate across shorter spatial scales, resulting in fewer long fibres and lower average LCF values. The binned FOC values are plotted against their corresponding LCF values in Fig. S14(C). Fitting the binned values, we find strong linear correlations between the FOC and LCF values.

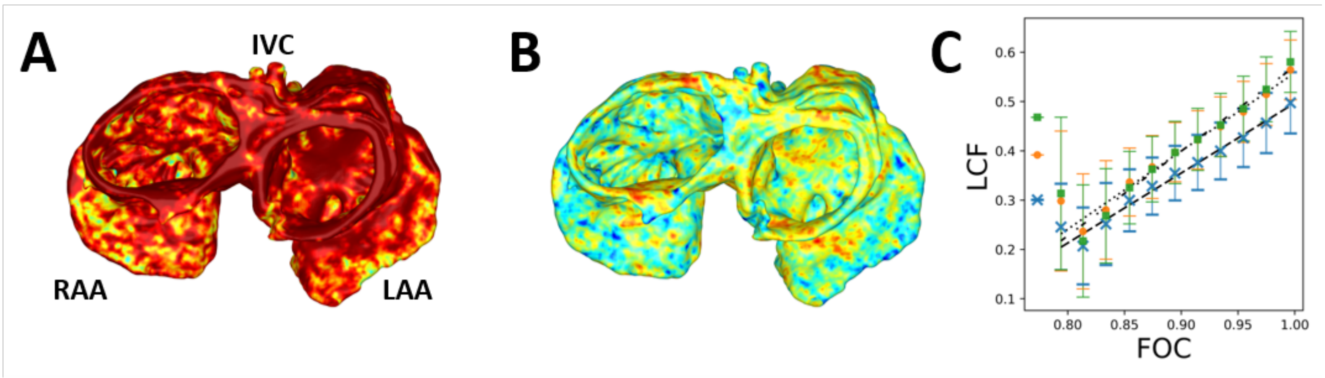

Figure S14. The fibre orientation correlation (FOC) and longitudinal connection fraction (LCF) for the HF sheep atria. (A) FOC. (B) LCF for low risk spatial network. (C) Binned LCF for all voxels in the HF sheep atria against their corresponding FOC values at high (blue crosses) medium (orange points) and low risk (green squares). High, medium and low risk data are linearly fitted (dashed, dotted and dot-dashed respectively) with weighted least squares. High risk, $R^2 = 0.965$. Medium risk, $R^2 = 0.971$. Low risk, $R^2 = 0.974$.

1.8 Connection anisotropy

In pioneering work, Spach and Dolber demonstrated that transverse connections between cardiac muscle fibres are progressively lost as the atria remodel with increasing age (Spach and Dolber (1986)). In particular, Spach and Dolber found that the progressive removal of connections between adjacent fibres resulted in significant anistropy in microscopic conduction velocities along and across fibres. Comparing atrial specimens from young patients with minimal structural remodelling, to older samples with extensive structural modelling, the authors found microscopic longitudinal to transverse conduction velocity ratios (Θ_L/Θ_T) of approximately 4.5 in the former case, and 9.8 in the latter with more extensive structural remodelling. In (Spach et al. (1988)) the authors note that at an anisotropy ratio of 9, atrial preparations start to exhibit longitudinal conduction block which may initiate micro-reentries.

For validation purposes, we would like to check that the fibre-based spatial networks result in similar anisotropy ratios. When defining the anisotropy, Spach et al. note that a ratio of 9 corresponds to "a rectangular circuit in which the length [is] nine times that of the width". We can approximate the average circuit length in regions of strong longitudinal coupling for the spatial networks directly using the risk calculations found in (Christensen et al. (2015); Ciacci et al. (2020)). This includes long circuits of length

$\ell \geq \tau$ which are susceptible to micro-reentry, and shorter loops of length $\ell < \tau$ which are too short for a micro-reentrant circuit to form.

In Fig. S2 we show that the modified ESS algorithm we use to construct the fibres in our spatial networks results in a uniform density of one node per voxel in each spatial network. For simplicity, let us assume that one node is positioned at the centre of each voxel. Consider the voxel v which has two neighbouring voxels in the x direction, two in the y direction, and two in the z direction. Let us assume that the principal fibre orientation is aligned with the x-axis, defined as the longitudinal direction, such that the y and z directions correspond to the transverse directions. Focusing on the x-y plane, the voxel v is connected with probability 1 to the two adjacent nodes along the x-axis, and is connected to the two nodes in the y direction with independent probability $p(x; r, c)$, given by

$$p(x; r, c) = \frac{1}{e^{r(x-c)} + 1}, \tag{S9}$$

where $r = 7$ and we set $x = 1$ since neighbouring nodes are, on average, separated by one voxel spacing. The variable c is the characteristic coupling distance for each network. For notational clarity we will write $p(x; r, c) = p$ for the remainder of this section. In order to calculate the anisotropy ratio, we would like to derive an expression for the typical circuit length ℓ^* using this formalism.

Let us assume node v is connected to its transverse neighbours. Node v is then part of a circuit of length ℓ if it is connected to $\ell - 1$ consecutive longitudinal neighbours along the x-axis with no transverse connections, each with probability $(1 - p)^2$, and the ℓth node has at least one transverse connection with probability $1 - (1 - p)^2$. Therefore, the probability that a circuit has length ℓ is

$$\mathcal{P}(\ell; p) = (1 - p)^{2(\ell-1)} \cdot \left[1 - (1 - p)^2\right]. \tag{S10}$$

Using this equation, we define the typical circuit length as the median length $\ell = \ell^*$ for a particular value of the connection probability p. This is easily calculated by writing,

$$\sum_{\ell=1}^{\ell=\ell^*} (1 - p)^{2(\ell-1)} \cdot \left[1 - (1 - p)^2\right] = 1 - (1 - p)^{2\ell^*} = 0.5, \tag{S11}$$

where we have substituted in the standard expression for the partial sum where $p < 1$.

Rearranging Eq. (S11) and taking the logarithm of each side, we find the median circuit length is

$$\ell^* = \frac{\ln(1/2)}{2 \cdot \ln(1 - p)}. \tag{S12}$$

Noting that such a circuit has width 1, that means that the effective longitudinal to transverse coupling anisotropy in the spatial networks is equal to ℓ^*. Figure S15 shows ℓ^* as a function of the characteristic coupling distance c in Eq. (S9). The largest value of c at which we observe a risk of micro-anatomical reentry in the fibre models is $c = 0.55$ for the HF sheep, corresponding to $\ell^* \approx 8.3$, and $c = 0.525$ for the healthy sheep and human atria, corresponding to $\ell^* \approx 9.8$. The smallest values of c tested in the spatial networks, corresponding to the high risk cases, is $c = 0.425$, giving $\ell^* \approx 19.6$.

These results indicate that the onset of micro-anatomical reentry in the spatial networks starts at anisotropy values which are comparable to the ratio of 9.8 observed in tissue with extensive structural remodelling in

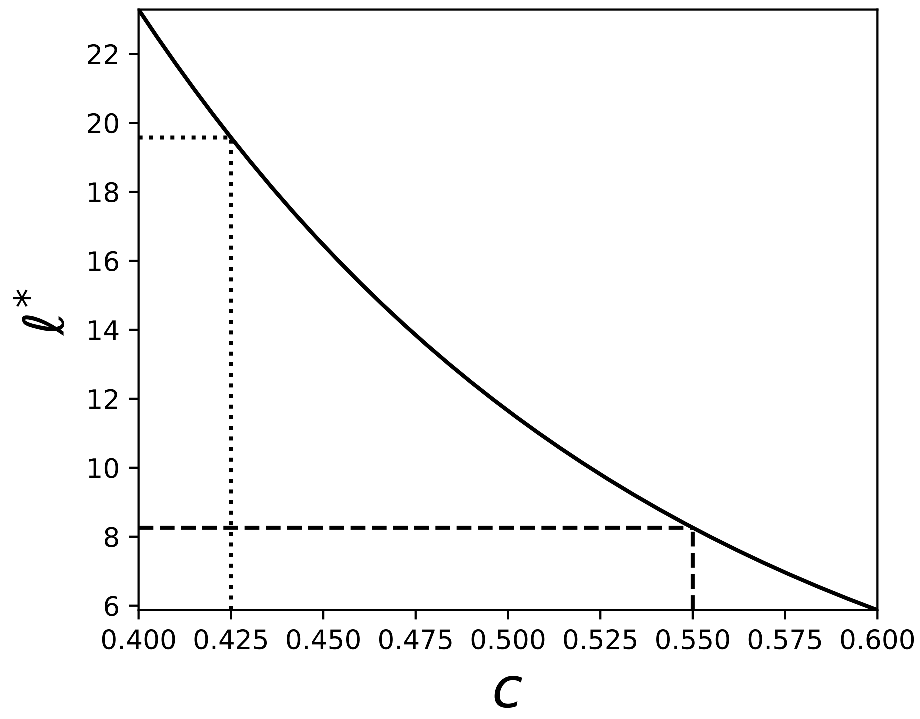

Figure S15. The median circuit length, ℓ^*, in regions of strong longitudinal coupling as a function of the characteristic coupling distance, c. The median circuit length is equal to the median value for the longitudinal to transverse coupling value. Dotted and dashed lines indicate the values of ℓ^* for the minimum and maximum characteristic coupling values used in the fibre spatial networks.

(Spach and Dolber (1986)), and larger than the values expected for healthy tissue with minimal structural remodelling. This is also the ratio at which Spach et al. find that atrial preparations start to exhibit longitudinal conduction block (Spach et al. (1988)).

We can check whether the analytical calculation for the anisotropy ratio is consistent with the spatial networks constructed with reference to the longitudinal connection fraction (LCF). Consider the $\ell + 1$ nodes that are longitudinally connected (along the x direction) to form an isolated fibre of length ℓ. Each node is connected longitudinally to two other nodes, totalling $2(\ell + 1)$ connections. For the fibre to be isolated, the $\ell - 1$ nodes in the centre of the fibre must have zero transverse connections. In contrast, the two nodes at the end of the fibre must have at least one transverse connection each to form a circuit, but may have up to four transverse connections each (one for each neighbouring node in the y and z directions). Hence, the total number of transverse connections for the circuit is typically between 2 and 8.

The LCF is defined as the average number of longitudinal connections, normalised by the total number of connections, either longitudinal or transverse. For the isolated fibre there are $2(\ell + 1)$ longitudinal connections,[3] and between $2(\ell + 1) + 2$ and $2(\ell + 1) + 8$ total connections. Substituting in the typical isolated fibre length ℓ^*, this suggests that the largest LCF values we should observe in each atrial dataset in regions of strong longitudinal coupling should fall within the range

$$\frac{2(\ell^* + 1)}{2(\ell^* + 1) + 8} \leq \mathrm{Max}(LCF) \leq \frac{2(\ell^* + 1)}{2(\ell^* + 1) + 2}. \qquad \text{(S13)}$$

[3] It is important to remember that each longitudinal edge in the isolated fibre is counted twice, since it connects to two of the nodes in the fibre.

Taking $\ell^* \approx 9$, this suggests the largest LCF values we should observe across an extended region of each atrial fibre map at low risk should be approximately

$$0.71 \leq \text{Max}(LCF) \leq 0.91. \tag{S14}$$

In regions of high longitudinal coupling, the largest LCF values observed after Gaussian smoothing is approximately 0.85 for the human atria, 0.88 for the healthy sheep atria, and 0.75 for the HF sheep atria, all of which fall within the accepted range. This implies that our analytical calculation is a good approximation for the degree of anisotropy in regions of strong longitudinal coupling for the three atrial datasets.

1.9 Technical Limitations

Assuming the imaging data used is of an acceptable quality and accuracy, a number of limitations arise in the fibre map modelling process. Firstly, the fibre tractography methods applied to generate global fibre tracts from local fibre orientation data are only an approximation of reality. Without performing precise histology to determine the position of global fibre tracts in our model, not histology focusing solely on local fibre orientation, it is not possible for us to explicitly validate this approach. Since the imaging data used was acquired from previous studies, such a process is not possible in the current work but may be possible in future studies. To mitigate the arbitrariness of any fibre tracts generated, we take a probabilistic view in which a large number of spatial networks are generated and results are aggregated over a wide sample. This ensures that no one fibre map dominates the final results, but a full histological validation of this approach would still be warranted in the future. As a crude check of the robustness of our results, the full simulation pipeline, including the regeneration of fibres from a different set of random seed points, was carried out three times for the healthy sheep heart, showing no evidence that this significantly effected our final results.

Once fibre tracts are generated, nodes are placed along the fibres, and are coupled to nearby nodes based on their separation. The attachment function to connect two nodes is uniform across the atrial structure and does not consider any possible differences in the local connectivity of cardiomyocytes. Likewise, the characteristic coupling, c, that is used in each model is constant across the atrial structure. This can be thought of as applying a uniform density of interstitial fibrosis across the atria. It is known that not all regions of the atria are equally susceptible to the accumulation of fibrosis (Benito et al. (2018)), and likewise, different forms of fibrosis accumulate differently across the atria (Anderson et al. (1979)). Our assumption of uniformity is a reasonable first approximation in the absence of patient specific information regarding the distribution of interstitial fibrosis which may not be visible with late GE-MRI. We do not consider any macroscopic fibrosis in the model. Such information was not available with the datasets acquired, but should be considered in any future work.

Once a spatial network has been constructed, the micro-reentrant substrate is identified by applying a simple discrete diffusion model on the network. The approach is loosely based on the techniques discussed in (Christensen et al. (2015); Falkenberg et al. (2019)), although we stress that its purpose here is strictly to identify locations in which isolated fibres result in micro-anatomical reentry, not to simulate AF dynamics. Assessing the ability of these structures to initiate and maintain AF would warrant a more detailed computational analysis with a phenomenologically accurate model which may be challenging at the resolution of our spatial network, and may struggle to produce large scale statistics.

The regions that are identified by the discrete diffusion model are a set of isolated fibre tracts where unidirectional conduction block at a single node is sufficient to induce a micro-reentrant circuit. The method to apply conduction block assumes uniform risk of block, avoiding the need to specify special electrical properties in key atrial locations. In practice, this may not be the case and the risk of block may vary significantly across the atria. For instance, the proarrhythmic conditions found in the PVs such as conduction velocity slowing and shortened action potential durations are not considered. However, our results do indicate that the PVs can emerge as a key risk substrate without the inclusion of electrical proarrhythmic effects.

Finally, before our approach can have real clinical relevance, it would benefit from further validation. We have demonstrated that the spatial networks accurately preserve the underlying properties of each atrial fibre map, and that our techniques ensure an even density of nodes across the network. For clinical relevance, it is important to validate the approach directly against experimental data, ideally with datasets

with a history of micro-anatomical reentry. If possible raw data should be available such that fibre maps can be generated using a range of methods at different levels of smoothing, and that the influence of small structural holes can be accurately tested. Data may be acquired at different levels of interstitial fibrotic density to assess the validity of our method's longitudinal predictions. If such an approach is not feasible, the method may be tested against advanced simulations of AF dynamics using the mono- or bidomain models in the presence of structural heterogeneities at high resolution.

2 SUPPLEMENTARY RESULTS

2.1 Spatial Distribution of Micro-reentrant substrate in the sheep atria

Figure S16 shows the spatial distribution of the micro-anatomical reentrant substrate for the healthy sheep atria at low, medium and high risk. The equivalent fibre-less null model is shown in Fig. S17, and additional views including atrial cross-sections are shown for both the fibre and fibre-less null models in Fig. S18. The corresponding figures for the HF sheep are shown in Fig. S19 (fibre model), Fig. S20 (fibre-less null model), and Fig. S21 (additional views). Specific examples showing how fibre structure affects the spatial distribution of micro-reentry is shown in section 2.1.3, alongside a statistical analysis of the role of the occupied voxel fraction and the longitudinal connection fraction.

2.1.1 Fibre model

At low risk, micro-reentrant circuits are almost exclusively clustered around the sleeve of the inferior vena cava (IVC). At medium risk, both sheep atria exhibit significant risk around the IVC and the RAA where the endocardium is dominated by ridged PMs. In contrast, the risk of reentry in the right and left atrial bulk, where the endocardium is smooth, is approximately zero. Particularly in the healthy sheep atria, a significant substrate arises along the PV sleeves, whereas for the heart failure sheep the risk along the PVs is non-zero, but not high. In the healthy sheep atria, there is noticeable risk at the opening of the CS, although this substrate is absent in the heart failure sheep atria, likely because the CS is sealed in this dataset.

Finally, at high risk the spatial distribution of the susceptible substrate is widespread for both sheep atria. Significant risk is observed along the IVC, at the PVs, and in both the right and left atria, including both atrial appendages where there are a large number of ridged PMs, although risk remains largely absent in regions where the endocardium is dominated by smooth tissue. Risk is maintained in the healthy atria at the opening of the CS, but is absent in the heart failure sheep.

2.1.2 Fibre-less Null Model

In agreement with the results for the human atrial dataset, the spatial distribution for the risk of micro-reentry is similar between the fibre and fibre-less null models for the sheep atrial datasets.

For the healthy sheep at low risk, the key risk region in both the fibre and fibre-less null models lies along the sleeve of the IVC. However, at medium and high risk, the fibre model shows diffuse risk across the atrial appendages whereas these risk regions are largely absent in the fibre-less null models. Conversely, the fibre-less null model shows extensive risk along the posterior right atrial wall and the opening of the SVC, whereas these regions are largely suppressed in the fibre model. Finally, the opening of the LIPV is identified as a key risk region in the fibre model, but risk in the fibre-less null model remains focused around the IVC at all risk levels, with only minimal risk observed at the LIPV.

For the HF sheep, the differences between the fibre and fibre-less null models are more pronounced, and there are noticeable differences between the healthy and HF sheep. At low risk, the IVC is the principal risk region in the fibre model, whereas the fibre-less null model shows extensive risk along an isolated tract of tissue bordering the TV. At medium and high risk, both the fibre and fibre-less models show risk across the atrial appendages. However, in the fibre model this risk is moderate and relatively spatially confined, whereas the fibre-less null model exhibits significantly higher risk across a wider region, particularly across the RAA. This is the opposite effect to the healthy sheep where diffuse risk is observed across the atrial appendages for the fibre model, but is largely absent in the fibre-less null model. The most likely explanation for these differences is the observation that the HF sheep is significantly thinner than the

healthy sheep atria, see Fig. S9, although differences in the resolution at which the datasets are acquired may also play a factor.

Other differences between the fibre and fibre-less null models for the HF sheep include the extensive risk along the right endocardial wall in the fibre-less null model which is suppressed in the fibre model. The fibre-less null model also demonstrates more extensive risk across the PV sleeves, whereas risk is low in these regions for the fibre model.

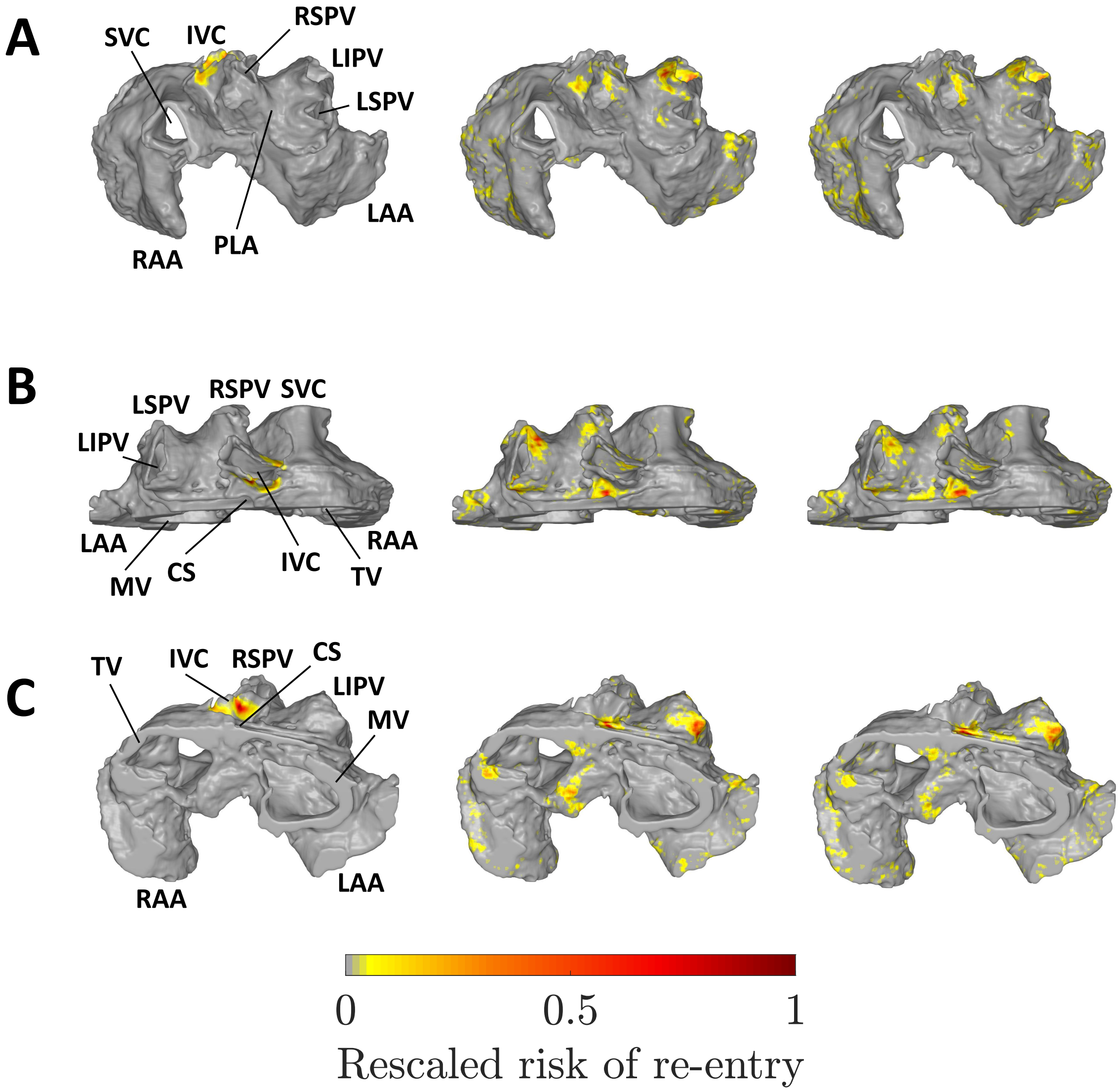

Figure S16. The spatial distribution of the micro-reentrant substrate in the fibre model at low (left), medium (middle) and high risk (right) for the healthy sheep atria. A: Superior view. B: Posterior view. C: Inferior view. IVC/SVC: Inferior/ superior vena cava. PLA: Posterior left atrium. RAA/LAA: Right/ left atrial appendage. TV/MV: Tricuspid/ mitral valve opening. CS: Coronary sinus. RIPV/LIPV/RSPV/LSPV: Right/ left, inferior/ superior, pulmonary vein.

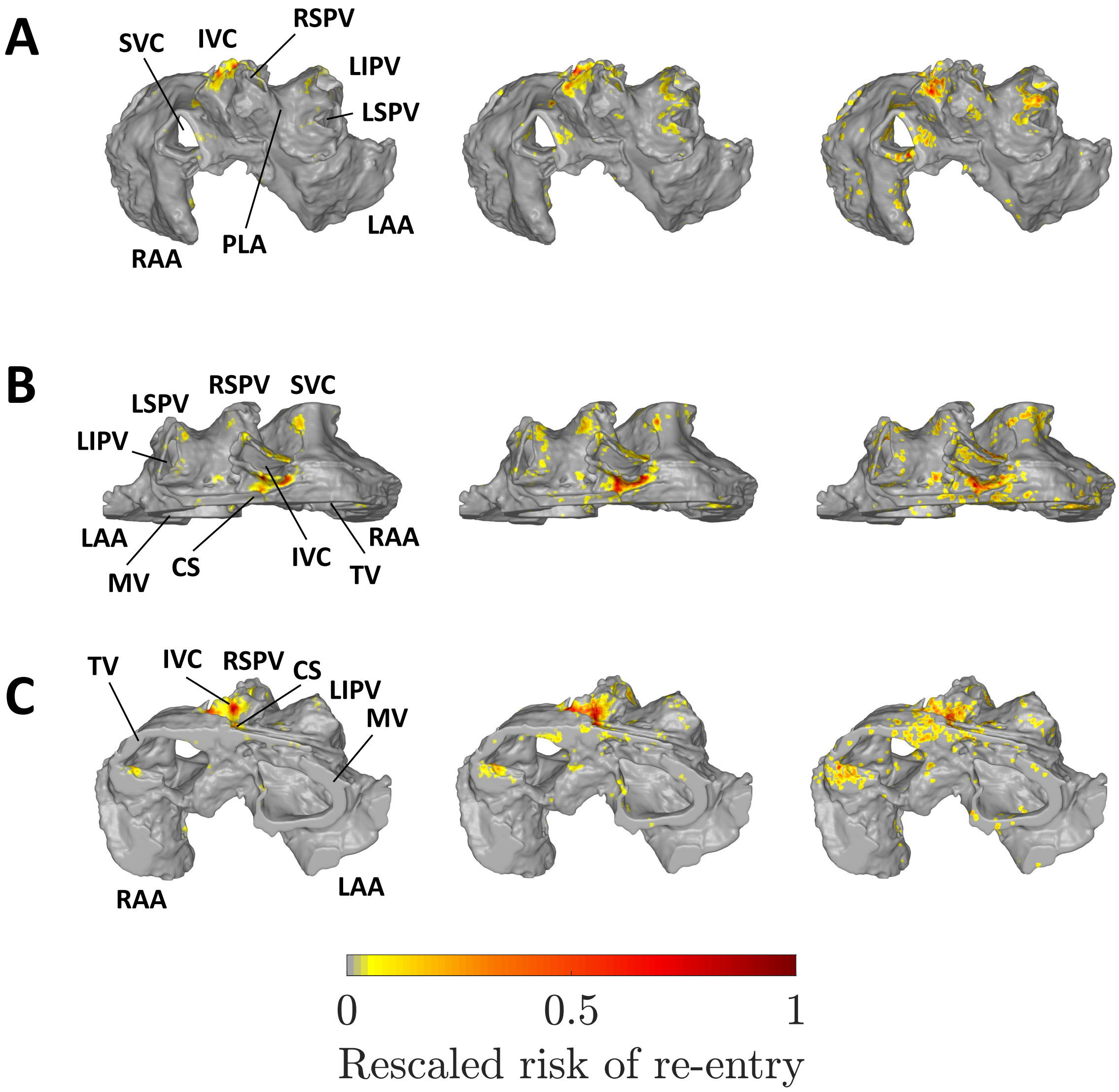

Figure S17. The spatial distribution of the micro-reentrant substrate in the fibre-less null model at low (left), medium (middle) and high risk (right) for the healthy sheep atria. A: Superior view. B: Posterior view. C: Inferior view. IVC/SVC: Inferior/ superior vena cava. PLA: Posterior left atrium. RAA/LAA: Right/ left atrial appendage. TV/MV: Tricuspid/ mitral valve opening. CS: Coronary sinus. RIPV/LIPV/RSPV/LSPV: Right/ left, inferior/ superior, pulmonary vein.

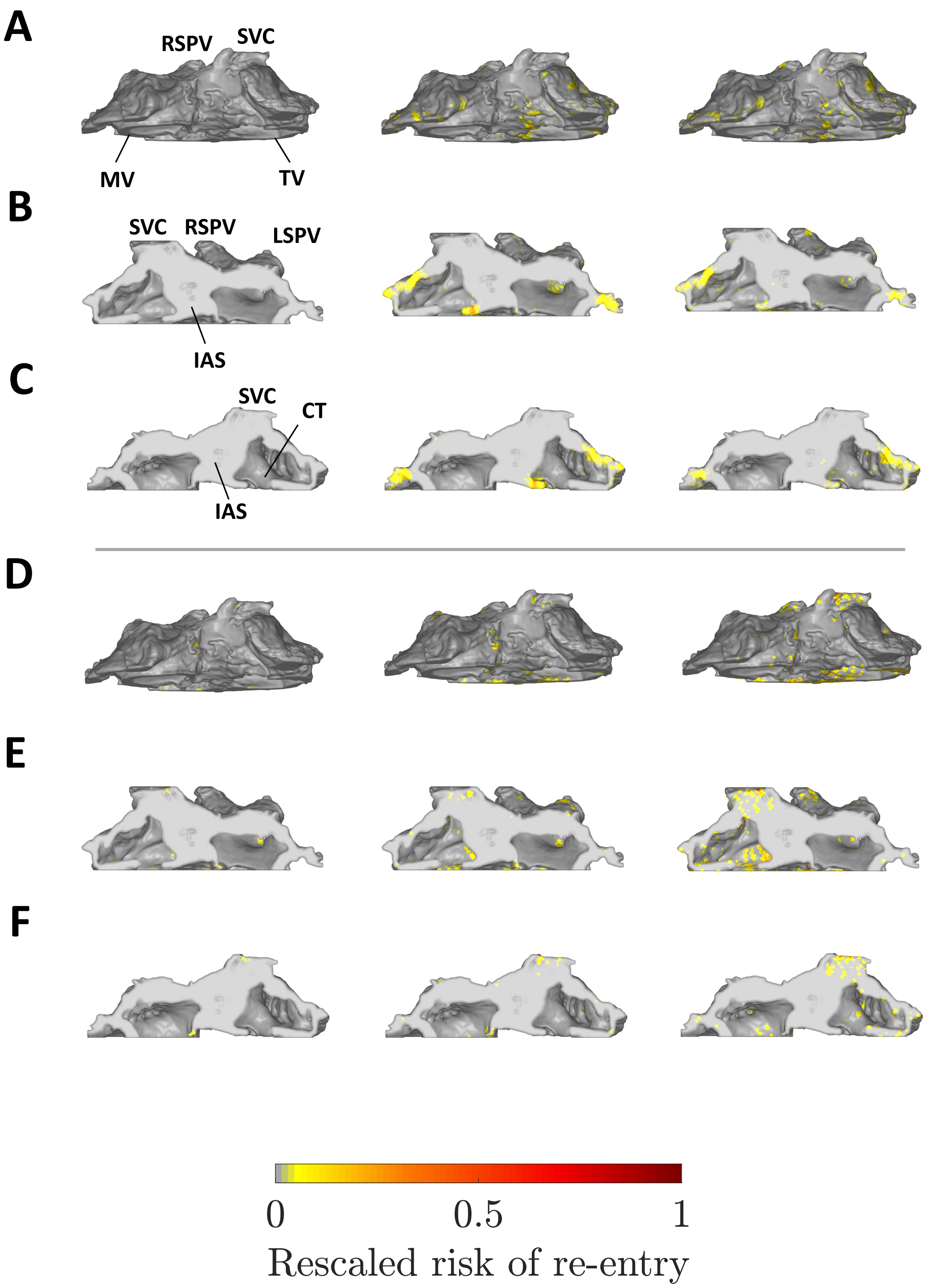

Figure S18. The spatial distribution of the micro-reentrant substrate for the healthy sheep atria in the fibre (A-C) and fibre-less null models (D-F) at low (left column), medium (middle) and high risk (right). A/D: Anterior view. B/E: Cut-through anterior view. C/F: Cut-through posterior view. SVC: Superior vena cava. CS: Coronary sinus. RSPV/LSPV: Right/ left, superior, pulmonary vein. IAS: Inter-atrial septum. CT: Crista terminalis. TV/MV: Tricuspid/ mitral valve opening.

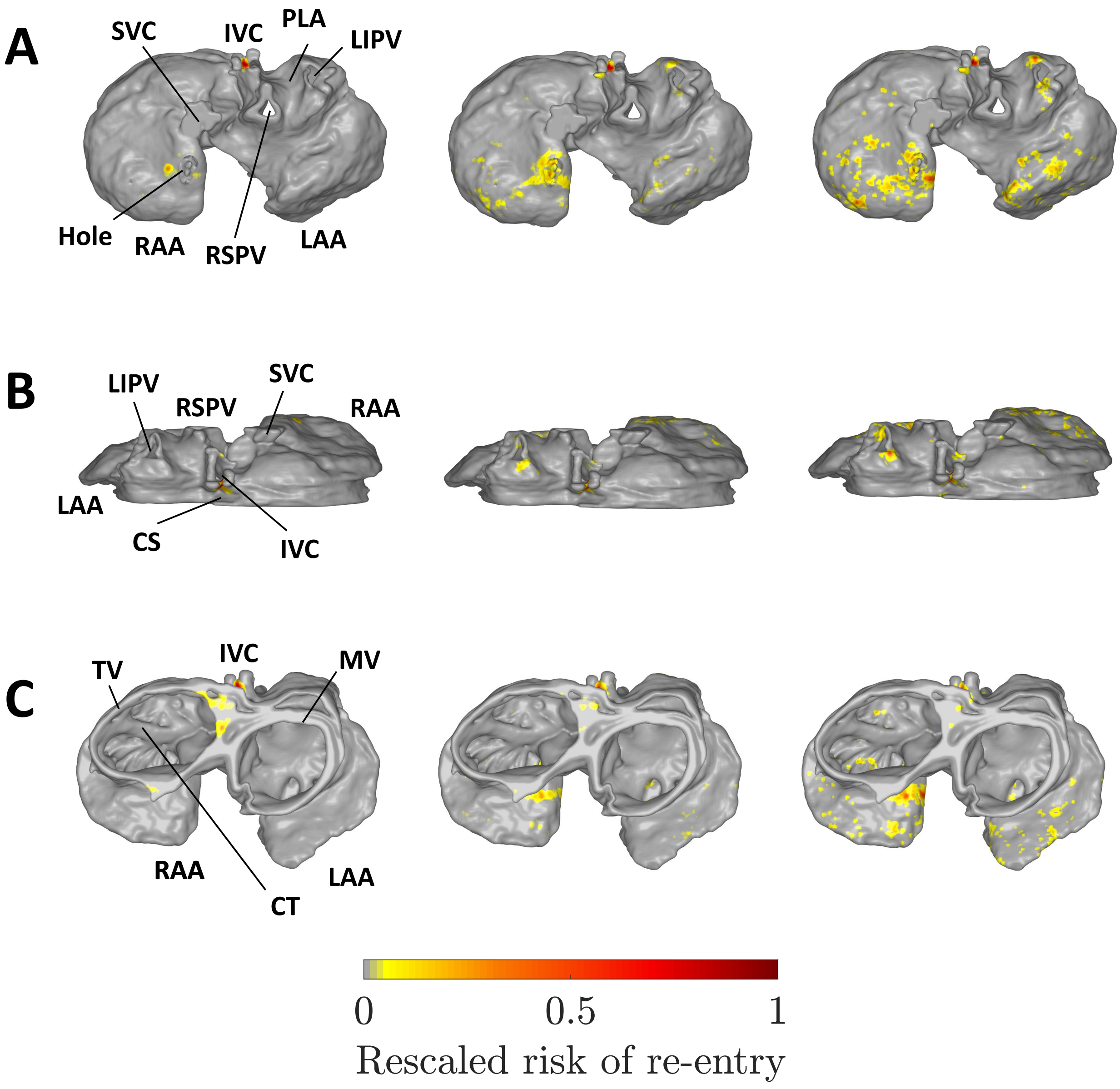

Figure S19. The spatial distribution of the micro-reentrant substrate in the fibre model at low (left), medium (middle) and high risk (right) for the HF sheep atria. A: Superior view. B: Posterior view. C: Inferior view. IVC/SVC: Inferior/ superior vena cava. PLA: Posterior left atrium. RAA/LAA: Right/ left atrial appendage. TV/MV: Tricuspid/ mitral valve opening. CS: Coronary sinus. RIPV/LIPV/RSPV/LSPV: Right/ left, inferior/ superior, pulmonary vein. The hole labelled in A is an artefact of imaging and is not an opening in the dataset.

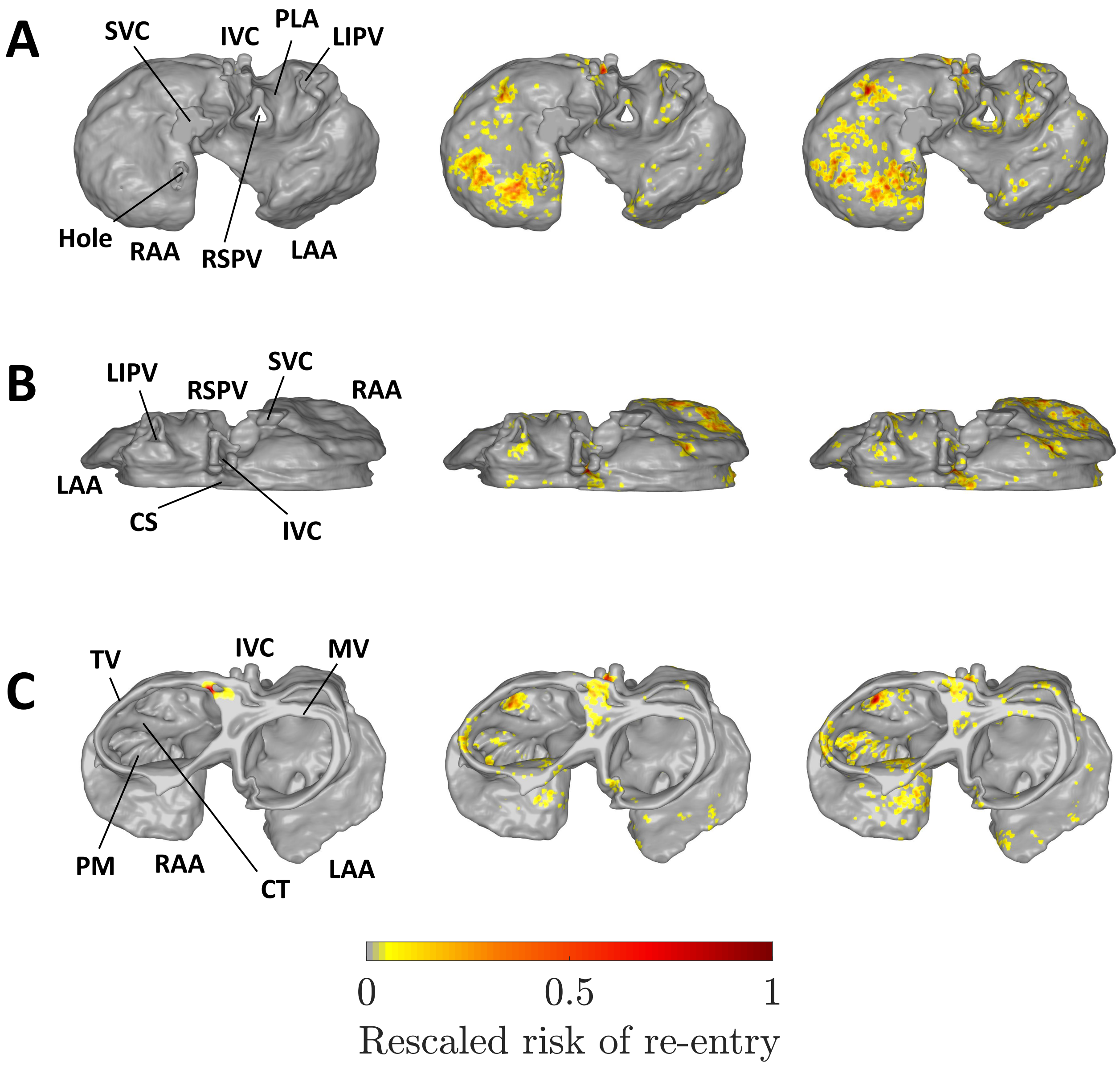

Figure S20. The spatial distribution of the micro-reentrant substrate in the fibre-less null model at low (left), medium (middle) and high risk (right) for the HF sheep atria. A: Superior view. B: Posterior view. C: Inferior view. IVC/SVC: Inferior/ superior vena cava. PLA: Posterior left atrium. RAA/LAA: Right/ left atrial appendage. TV/MV: Tricuspid/ mitral valve opening. CS: Coronary sinus. RIPV/LIPV/RSPV/LSPV: Right/ left, inferior/ superior, pulmonary vein.

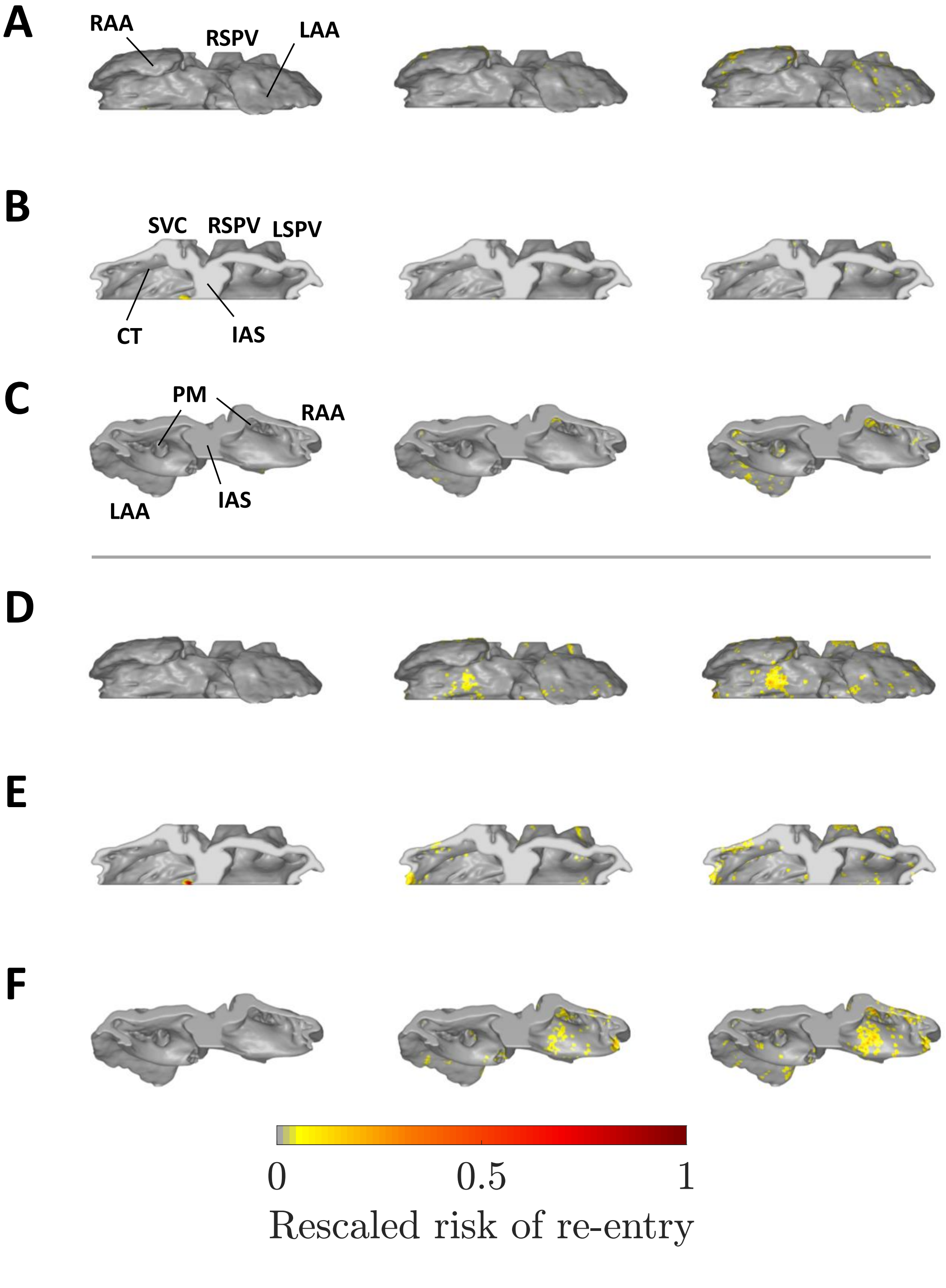

Figure S21. The spatial distribution of the micro-reentrant substrate for the heart failure sheep in the fibre (A-C) and fibre-less null models (D-F) at high (left column), medium (middle) and low coupling (right). A/D: Anterior view. B/E: Cut-through anterior view. C/F: Cut-through posterior view. SVC: Superior vena cava. CS: Coronary sinus. RSPV/LSPV: Right/ left, superior, pulmonary vein. IAS: Inter-atrial septum. CT: Crista terminalis. RAA/LAA: Right/ left atrial appendage. PM: Pectinate muscles.

2.1.3 Effect of fibre structure in sheep atria

Figure S22 shows an example of how the OVF and LCF effect the observed spatial distribution of micro-reentrant risk in the fibre and fibre-less null models for the healthy sheep atria. The example shows the inferior view, with three regions marked for reference. Figure S22(A) shows the distribution of risk for the fibre model at medium risk. Figure S22(B) shows the equivalent for the fibre-less null model.

In the fibre model, risk is observed at the border of the TV (region 1), along the sleeve of the LIPV (region 2) and diffuse risk is present along the bottom of the LAA (region 3). In the fibre-less models, risk is largely absent in these regions, with the dominant risk substrate remaining along the IVC where the OVF is low. The IVC is observed as a risk substrate in the fibre model at low overall risk when the effect of the OVF is most dominant, see Fig. S16. However, at medium risk, the three identified risk substrates for the fibre model are all in regions with low LCF but moderate OVF; recall the highest OVF regions are not visible since they are in the atrial bulk. This illustrates that weak longitudinal coupling can enhance the risk of micro-reentry in the fibre model compared to the equivalent fibre-less null model.

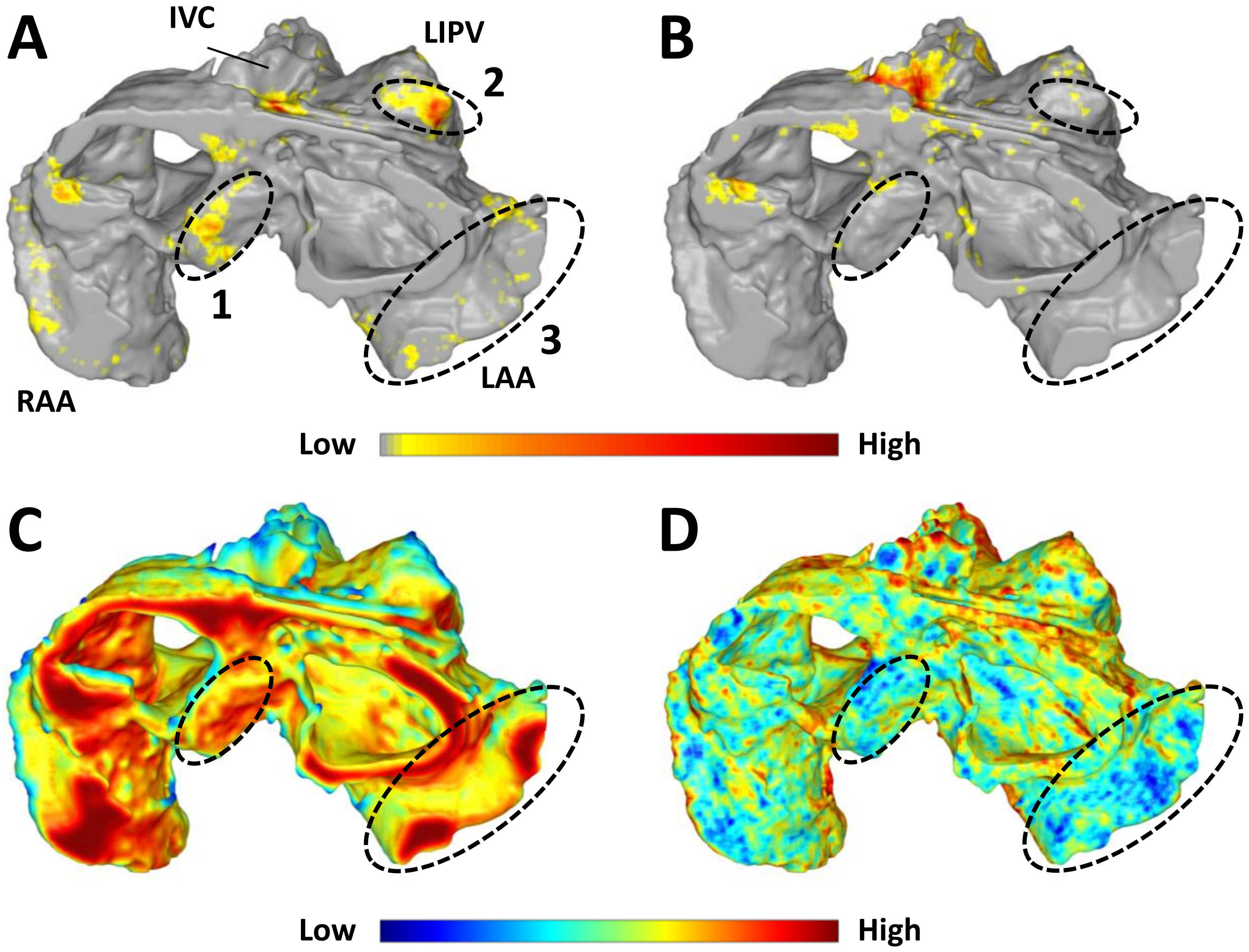

Figure S22. A view of the healthy sheep from the inferior view illustrating factors influencing the spatial distribution of risk in (A) the fibre model, and (B) the fibre-less null model. (C) The occupied voxel fraction (OVF). (D) The longitudinal connection fraction (LCF). Regions (1–3) are referenced in the main text.

Figure S23 shows an example of how the OVF and LCF effect the observed spatial distribution of micro-reentrant risk in the fibre and fibre-less null models for the HF sheep atria. The example shows the anterior view, with one marked region for reference. Figure S23(A) shows the distribution of risk for the fibre model at high risk. Figure S23(B) shows the equivalent for the fibre-less null model. Although the effect of OVF and LCF is statistically significant for the HF sheep, we note that this example is the most difficult to visually interpret when compared to the healthy sheep and human atria, mainly due to the short spatial scales over which the LCF fluctuates. Focusing on region 1, we note that this region of the RA wall is at a lower OVF value than the surrounding region, but has relatively high LCF values. As a result, the OVF shows significant risk in this region, whereas risk is completely suppressed in this region for the fibre model.

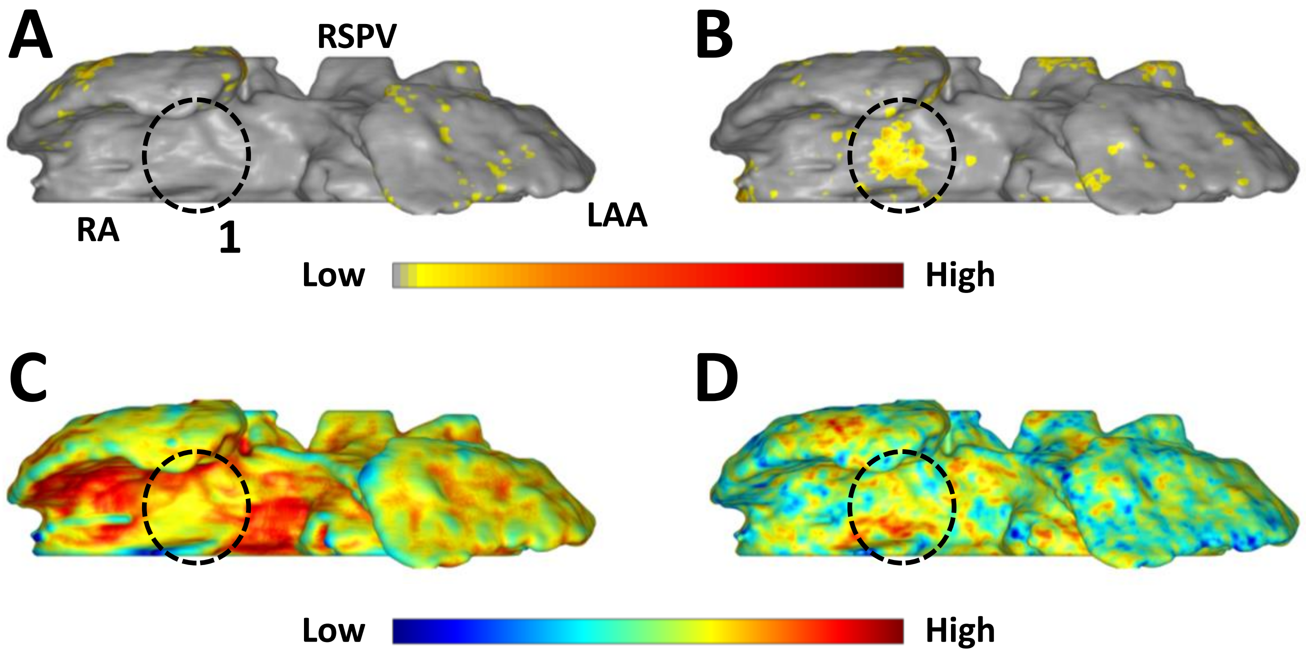

Figure S23. A view of the HF sheep from the anterior view illustrating factors influencing the spatial distribution of risk in (A) the fibre model, and (B) the fibre-less null model. (C) The occupied voxel fraction (OVF). (D) The longitudinal connection fraction (LCF). Region (1) is referenced in the main text.

Quantifying these observations, Figs. S24 and S25 show the distribution of OVF and LCF values in the healthy and HF sheep atria respectively. Each figure shows the distribution where each voxel is weighted equally, weighted according to the risk of micro-anatomical reentry for the fibre model, and weighted according to the risk in the fibre-less null model, each at low and high overall risk. The figures are qualitatively consistent with the data for the human atria shown in Fig. 13 of the main manuscript, although effect sizes are smaller, see below.

Note that in particular for the healthy sheep atrium, the densest region of the OVF and LCF distribution where voxels are uniformly weighted (Figs. S24(A) and (D)) is at OVF ~ 1. This indicates that the majority of voxels in the healthy sheep atria are far from the atrial walls. This is not the case for the human atria or the HF sheep atria.

For the low risk cases in both sheep, the mean of the distributions weighted by both the fibre and fibre-less null models falls at significantly lower OVF values than the uniform case. In particular, no risk of micro-reentry is observed in regions where the OVF is above the 50th percentile, suggesting a complete suppression of micro-reentry in thick regions of the atria.

At high risk, the bias to low OVF values is significantly reduced, but a small bias is still visible. In the healthy sheep fibre model, risk is observed at an OVF ~ 1, but only in regions with reduced LCF. In contrast, micro-reentry is completely suppressed in regions with OVF ~ 1 in the fibre-less null model. This indicates that reduced longitudinal fibre coupling enables micro-reentry in thicker regions of the atria at high risk.

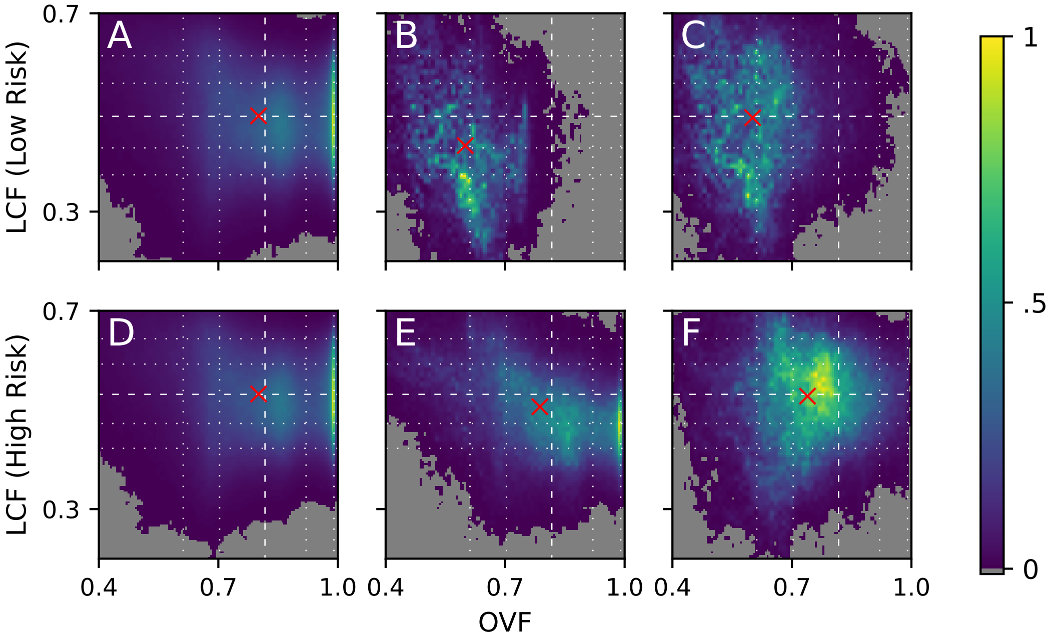

Figure S24. The distribution of LCF and OVF values in the healthy sheep atria at low (A-C) and high (D-F) risk. (A) & (D) The distribution across all the voxels in the healthy sheep atria. (B) & (E) The distribution weighted by the relative risk of reentry in each voxel for the fibre model. (C) & (F) The equivalent for the fibre-less null model. Note, the LCF is not defined for the fibre-less null model, but for illustrative purposes we include the LCF for the equivalent fibre model. Dashed lines: median. Dotted lines: 10th, 25th, 75th and 90th percentiles. Colourbar indicates voxel density relative to distribution maximum; regions in grey correspond to a relative density of zero. Red crosses: weighted mean of distribution.

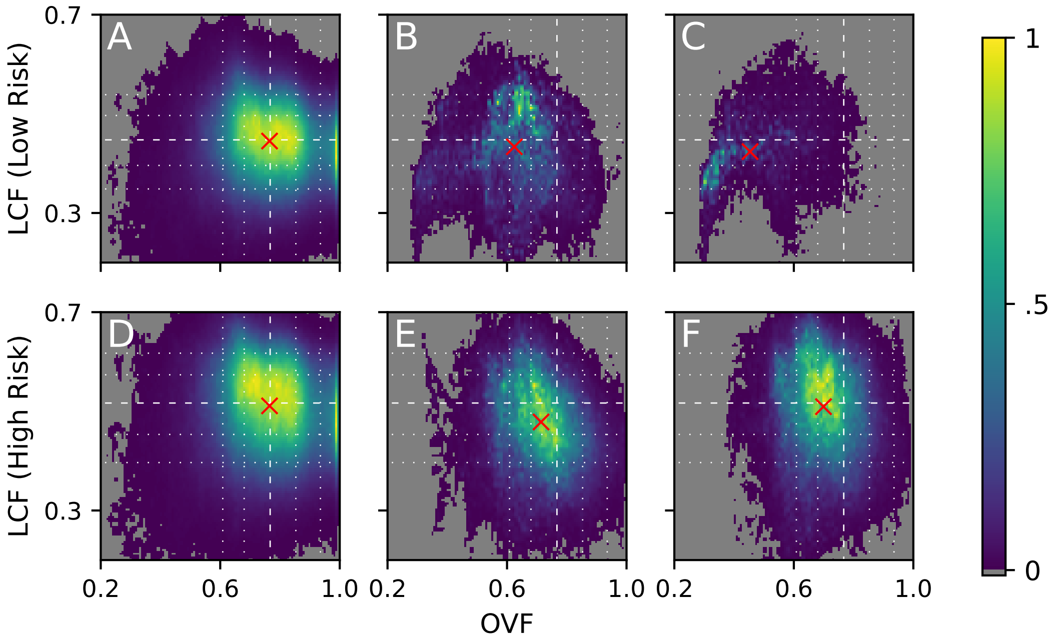

Figure S25. The distribution of LCF and OVF values in the HF sheep atria at low (A-C) and high (D-F) risk. (A) & (D) The distribution across all the voxels in the human atria. (B) & (E) The distribution weighted by the relative risk of reentry in each voxel for the fibre model. (C) & (F) The equivalent for the fibre-less null model. Note, the LCF is not defined for the fibre-less null model, but for illustrative purposes we include the LCF for the equivalent fibre model. Dashed lines: median. Dotted lines: 10th, 25th, 75th and 90th percentiles. Colourbar indicates voxel density relative to distribution maximum; regions in grey correspond to a relative density of zero. Red crosses: weighted mean of distribution.

2.2 Comparing synthetic and anatomically derived fibre structures

The distributions of micro-reentrant risk for the anatomically derived sheep atria are shown in SM section 2.1. Qualitatively, our results are robust across all three datasets. At low risk, the micro-reentrant substrate is highly clustered in a small number of regions. These regions correspond to areas of low OVF. In the fibre-less null models, this bias reduces with increasing risk. In particular, the probability of observing micro-reentry in the null model at OVF ~ 1 is approximately zero across all spatial networks. In the fibre models, a bias to low OVF is observed, but the effect is smaller than for the fibre-less null models.

In the fibre models, there is a bias at all risk levels to areas of reduced longitudinal coupling (LCF). However, effect sizes are significantly larger for the synthetic human atrial dataset than for either sheep atrial dataset, and the effect is less influential than the bias to low OVF, see statistics below. For the HF sheep, at medium and low risk we find a small bias to areas of increased LCF, although visual inspection suggests that these locations correspond to regions with particularly low OVF values. The healthy sheep atria at high risk is the only spatial network in which we observe any significant micro-reentrant risk in regions with OVF ~ 1. However, the LCF values for the majority of those voxels falls around the 25th percentile, suggesting that only with reduced longitudinal coupling can micro-reentry emerge in thick regions of the atrial bulk.

These results suggest that the spatial network approach is relatively robust for datasets with both synthetic and anatomically derived fibre structures. Despite this, the robustness of the method cannot correct for the intrinsic limitations of individual datasets. In particular, there are two key differences between the synthetic human dataset and the anatomically derived sheep datasets which appear to affect our results.

Firstly, the local fibre heterogeneity in the human atria is not realistic, with individual heterogeneities likely arising at the boundaries of the user-defined fibre bundles, see section 1.1. As a consequence, over 90% of the voxels in the datasets are in regions with very high fibre alignment ($FOC > 0.9$), artificially suppressing micro-reentry across vast regions of the atria, and promoting micro-reentry artificially along fibre bundle boundaries. In this proof of concept, these limitations are acceptable for testing our methods, but the results derived from the fibre-model cannot accurately reflect how a real human fibre structure may effect the substrate for micro-reentry. Although both sheep atrial datasets have undergone some smoothing, heterogeneities in these datasets have been histologically validated and are distributed with finer detail across the atria.

The second difference concerns the pre-processing and resolution of the atrial structures. Although both sheep datasets were down-resolved to a voxel resolution that is comparable to the human resolution, visual inspection suggests that the sheep still retain significantly finer structural detail than the human atria. In particular, the fine muscular ridges of the PMs (with low OVF) are preserved in the RAA and LAA in the sheep atria, whereas these regions are completely smoothed in the human. Consequently, the sheep exhibit significant micro-reentrant risk in the RAA and LAA, whereas the human atria show no risk in the LAA, and minimal structural detail at the entrance to the RAA. Conversely, where small structural holes have been sealed in the sheep atria, numerous small holes are found in the human atria, particularly in the superior right atrium and the PV sleeves, where extensive micro-reentrant risk is observed. These regions are susceptible to micro-reentry even at high risk in the human where most of the OVF bias is lost in the sheep atria. This suggests that small inhomogeneities in the atrial structure have a significant impact on where micro-reentry is observed and how extensive the bias to regions with low OVF is.

2.3 Statistical analysis of OVF and LCF distributions

To test for statistically significant differences between the LCF and OVF distributions, we apply a permutation test (Efron and Tibshirani (1994)). Permutation tests are non-parametric and therefore avoid issues that arise due to the non-normality of the OVF distributions for each dataset. LCF values are mostly normally distributed, but we use the permutation test in both cases for consistency.

For Gaussian distributions, a standard measure for the effect size quantifying the difference between two distributions is Cohen's d, defined as

$$d = \frac{\bar{X} - \bar{Y}}{s}, \tag{S15}$$

where $\bar{X}$ and $\bar{Y}$ are the mean values of the two distributions, and s is the pooled standard deviation. Since the OVF distributions are non-normal, we apply the nonparametric analogue of Cohen's d by using the *median absolute deviation*, defined as

$$MAD(X) = C \cdot med\left(|X_i - med(X)|\right), \tag{S16}$$

where X_i is the i-th sample from the distribution X, $med(\cdot)$ denotes the median, and $C = 1.4826$ is a consistency constant that ensures the nonparametric Cohen's d maps to the regular Cohen's d for normal

distributions. Using this form, we can also define the *pooled median absolute deviation* as

$$PMAD(X,Y) = \sqrt{\frac{(n_X - 1) \cdot MAD^2(X) + (n_Y - 1) \cdot MAD^2(Y)}{n_x + n_Y - 2}}. \tag{S17}$$

Hence, we reach the non-parametric equivalent of Cohen's d by replacing the means in Eq. (S15) with the medians, and replacing the pooled standard deviation with $PMAD(X,Y)$, written as

$$d_{NP} = \frac{med(X) - med(Y)}{PMAD(X,Y)}, \tag{S18}$$

which reduces to Cohen's d for a normal distribution. For more details on this method see (Wilcox (2019)).

2.3.1 Statistical analysis of OVF and LCF distributions

Tables S4, S5, and S6 summarise the statistical impact of the occupied voxel fraction in the human, healthy sheep and HF sheep respectively. All tests are carried out using a permutation test with 10,000 random permutations of each pooled distribution. The null hypothesis is that the two tested samples are drawn from the same underlying distribution. The equivalent tests are shown for the LCF distributions in Tables S7, S8 and S9

In each table we compare the OVF (or LCF) distribution for voxels identified as the micro-reentrant substrate in the fibre model at low, medium and high risk, against an equally sized sample of voxels drawn uniformly at random from throughout the atrial geometry. The equivalent is provided for each of the fibre-less null models, tested against uniform sampling. Finally, we test the fibre models against their corresponding fibre-less null models at low, medium and high risk.

For the OVF, we find statistically significant differences ($p < 0.01$) for all distribution comparisons, with the exception of the high risk fibre model compared to the uniform distribution in the healthy sheep atria (see Table S8) where we find $p = 0.06$.

Distribution X	Distribution Y	$\lvert\bar{X} - \bar{Y}\rvert$	$\lvert med(X) - med(Y)\rvert$	p-value	Effect size	Descriptor
Fibre Low Risk	Uniform	0.313	0.336	0	1.95	Huge
Fibre Medium Risk	Uniform	0.200	0.238	0	1.29	Very Large
Fibre High Risk	Uniform	0.117	0.141	0	0.77	Large
Null Low Risk	Uniform	0.240	0.282	0	1.59	Very Large / Huge
Null Medium Risk	Uniform	0.216	0.254	0	1.46	Very Large
Null High Risk	Uniform	0.160	0.190	0	1.02	Large / Very Large
Fibre Low Risk	Null Low Risk	0.071	0.060	0	0.79	Large
Fibre Medium Risk	Null Medium Risk	0.013	0.009	0.001	0.07	Small
Fibre High Risk	Null High Risk	0.041	0.047	0	0.36	Medium

Table S4. Occupied voxel fraction statistical tests in the human atria using a permutation test. Effect size given by nonparametric Cohen's d. Descriptors for nonparametric Cohen's d from (Sawilowsky (2009)).

Focusing on the comparison between the risk distributions and the uniform distributions, we note that the effect sizes are largest for the low risk cases, moderate for the medium risk cases, and lowest for the high risk cases, demonstrating the reduction in the bias to low OVF values with increasing risk. Despite this,

Distribution X	Distribution Y	$\lvert\bar{X}-\bar{Y}\rvert$	$\lvert med(X)-med(Y)\rvert$	p-value	Effect size	Descriptor
Fibre Low Risk	Uniform	0.227	0.290	0	2.49	Huge
Fibre Medium Risk	Uniform	0.011	0.039	0.002	0.26	Small / Medium
Fibre High Risk	Uniform	0.006	0.019	0.06	0.15	Small
Null Low Risk	Uniform	0.203	0.272	0	2.20	Huge
Null Medium Risk	Uniform	0.142	0.223	0	2.05	Huge
Null High Risk	Uniform	0.059	0.111	0	0.81	Large
Fibre Low Risk	Null Low Risk	0.020	0.017	0	0.14	Small
Fibre Medium Risk	Null Medium Risk	0.127	0.164	0	1.09	Large / Very Large
Fibre High Risk	Null High Risk	0.064	0.095	0	0.69	Large

Table S5. Occupied voxel fraction statistical tests in the healthy sheep atria using a permutation test. Effect size given by nonparametric Cohen's d. Descriptors for nonparametric Cohen's d from (Sawilowsky (2009)).

Distribution X	Distribution Y	$\lvert\bar{X}-\bar{Y}\rvert$	$\lvert med(X)-med(Y)\rvert$	p-value	Effect size	Descriptor
Fibre Low Risk	Uniform	0.183	0.217	0	1.41	Very Large
Fibre Medium Risk	Uniform	0.055	0.081	0	0.44	Medium
Fibre High Risk	Uniform	0.043	0.056	0	0.31	Medium
Null Low Risk	Uniform	0.347	0.402	0	2.36	Huge
Null Medium Risk	Uniform	0.101	0.135	0	0.76	Large
Null High Risk	Uniform	0.055	0.094	0	0.53	Medium
Fibre Low Risk	Null Low Risk	0.170	0.192	0	1.71	Very Large / Huge
Fibre Medium Risk	Null Medium Risk	0.043	0.050	0	0.38	Medium
Fibre High Risk	Null High Risk	0.013	0.021	0.008	0.15	Small

Table S6. Occupied voxel fraction statistical tests in the HF sheep atria using a permutation test. Effect size given by nonparametric Cohen's d. Descriptors for nonparametric Cohen's d from (Sawilowsky (2009)).

even at the highest risk levels a small bias to reduced OVF values is retained (except for high risk fibre model in healthy sheep). Comparing the fibre models to the fibre-less null models, at medium and high risk, there is evidence that the bias to small OVF values is stronger in the fibre-less null models than the fibre models.

For the LCF, we find statistically significant differences for all distribution comparisons with the exception of: (1) human null high risk vs. uniform, healthy sheep null low risk vs. uniform, HF sheep null medium risk vs. uniform, and HF sheep null high risk vs. uniform. We do not expect any of the null distributions to be significantly different from the uniform case since the LCF should have no effect in the fibre-less null models. Any differences which are observed are indirect and are most likely due to small correlations between OVF and LCF values. In particular, for spatially isolated ridges such as the PMs, we often find that these regions of low OVF also correspond to regions of high LCF. This is unsurprising since these isolated ridges must have strong longitudinal coupling in order to preserve conduction along the isolated fibre. For those null distributions which are significantly different to the uniform case, we note that the effect sizes are medium or smaller. As expected when comparing the fibre and fibre-less null models, we find statistically significant differences between the LCF distributions, particularly at medium and high risk.

Overall, the effect sizes for the LCF are generally smaller than the equivalent OVF effect sizes. This indicates that OVF is the dominant factor in determining the micro-reentrant substrate in the spatial networks.

Distribution X	Distribution Y	$\|\bar{X} - \bar{Y}\|$	$\|med(X) - med(Y)\|$	p-value	Effect size	Descriptor
Fibre Low Risk	Uniform	0.036	0.034	0	0.81	Large
Fibre Medium Risk	Uniform	0.034	0.031	0	0.59	Medium / Large
Fibre High Risk	Uniform	0.021	0.022	0	0.48	Medium
Null Low Risk	Uniform	0.017	0.021	0	0.39	Medium
Null Medium Risk	Uniform	0.014	0.018	0	0.33	Medium
Null High Risk	Uniform	0.002	0.007	0.015	0.13	Small
Fibre Low Risk	Null Low Risk	0.053	0.055	0	1.03	Large / Very Large
Fibre Medium Risk	Null Medium Risk	0.048	0.049	0	0.83	Large
Fibre High Risk	Null High Risk	0.023	0.029	0	0.54	Medium

Table S7. Longitudinal connection fraction statistical tests in the human atria using a permutation test. Effect size given by nonparametric Cohen's d. Descriptors for nonparametric Cohen's d from (Sawilowsky (2009)).

Distribution X	Distribution Y	$\|\bar{X} - \bar{Y}\|$	$\|med(X) - med(Y)\|$	p-value	Effect size	Descriptor
Fibre Low Risk	Uniform	0.058	0.066	0	0.63	Medium / Large
Fibre Medium Risk	Uniform	0.035	0.038	0	0.42	Medium
Fibre High Risk	Uniform	0.026	0.030	0	0.35	Medium
Null Low Risk	Uniform	0.003	0.005	0.018	0.04	Very Small
Null Medium Risk	Uniform	0.006	0.001	0	0.01	Very Small
Null High Risk	Uniform	0.005	0.003	0	0.03	Very Small
Fibre Low Risk	Null Low Risk	0.055	0.071	0	0.59	Medium / Large
Fibre Medium Risk	Null Medium Risk	0.028	0.039	0	0.40	Medium
Fibre High Risk	Null High Risk	0.021	0.032	0	0.38	Medium

Table S8. Longitudinal connection fraction statistical tests in the healthy sheep atria using a permutation test. Effect size given by nonparametric Cohen's d. Descriptors for nonparametric Cohen's d from (Sawilowsky (2009)).

Distribution X	Distribution Y	$\|\bar{X} - \bar{Y}\|$	$\|med(X) - med(Y)\|$	p-value	Effect size	Descriptor
Fibre Low Risk	Uniform	0.011	0.008	0	0.10	Small
Fibre Medium Risk	Uniform	0.032	0.036	0	0.42	Medium
Fibre High Risk	Uniform	0.034	0.038	0	0.43	Medium
Null Low Risk	Uniform	0.021	0.025	0	0.38	Medium
Null Medium Risk	Uniform	0.002	0.001	0.085	0.007	Small
Null High Risk	Uniform	0.002	0.001	0.023	0.010	Small
Fibre Low Risk	Null Low Risk	0.010	0.017	0	0.23	Small / Medium
Fibre Medium Risk	Null Medium Risk	0.030	0.036	0	0.39	Medium
Fibre High Risk	Null High Risk	0.031	0.039	0	0.43	Medium

Table S9. Longitudinal connection fraction statistical tests in the HF sheep atria using a permutation test. Effect size given by nonparametric Cohen's d. Descriptors for nonparametric Cohen's d from (Sawilowsky (2009)).

REFERENCES

Alonso, S., dos Santos, R. W., and Bär, M. (2016). Reentry and ectopic pacemakers emerge in a three-dimensional model for a slab of cardiac tissue with diffuse microfibrosis near the percolation threshold. *PloS one* 11, e0166972

Anderson, K., Sutton, M., and Lie, J. (1979). Histopathological types of cardiac fibrosis in myocardial disease. *The Journal of pathology* 128, 79

Bærentzen, J. (2001). *On the implementation of fast marching methods for 3D lattices*

Benito, E. M., Cabanelas, N., Nuñez-Garcia, M., Alarcón, F., Figueras I Ventura, R. M., Soto-Iglesias, D., et al. (2018). Preferential regional distribution of atrial fibrosis in posterior wall around left inferior pulmonary vein as identified by late gadolinium enhancement cardiac magnetic resonance in patients with atrial fibrillation. *Ep Europace* 20, 1959–1965

Caan, M. W. A. (2016). *DTI Analysis Methods: Fibre Tracking and Connectivity* (New York, NY: Springer New York). 205–228. doi:10.1007/978-1-4939-3118-7_11

Christensen, K., Manani, K. A., and Peters, N. S. (2015). Simple model for identifying critical regions in atrial fibrillation. *Phys. Rev. Lett.* 114, 028104. doi:10.1103/PhysRevLett.114.028104

Ciacci, A., Falkenberg, M., Manani, K. A., Evans, T. S., Peters, N. S., and Christensen, K. (2020). Understanding the transition from paroxysmal to persistent atrial fibrillation. *Physical Review Research* 2, 023311

Dharmaprani, D., Jenkins, E., Aguilar, M., Quah, J. X., Lahiri, A., Tiver, K., et al. (2021). M/m/infinity birth-death processes – a quantitative representational framework to summarize and explain phase singularity and wavelet dynamics in atrial fibrillation. *Frontiers in Physiology* 11, 1786. doi:10.3389/fphys.2020.616866

Dharmaprani, D., Schopp, M., Kuklik, P., Chapman, D., Lahiri, A., Dykes, L., et al. (2019). Renewal theory as a universal quantitative framework to characterize phase singularity regeneration in mammalian cardiac fibrillation. *Circulation: Arrhythmia and Electrophysiology* 12, e007569. doi:10.1161/CIRCEP.119.007569

Efron, B. and Tibshirani, R. J. (1994). *An introduction to the bootstrap* (CRC press)

Falkenberg, M., Ford, A. J., Li, A. C., Lawrence, R., Ciacci, A., Peters, N. S., et al. (2019). Unified mechanism of local drivers in a percolation model of atrial fibrillation. *Physical Review E* 100, 062406

Hansen, B. J., Zhao, J., Csepe, T. A., Moore, B. T., Li, N., Jayne, L. A., et al. (2015). Atrial fibrillation driven by micro-anatomic intramural re-entry revealed by simultaneous sub-epicardial and sub-endocardial optical mapping in explanted human hearts. *Eur. Heart J.* 36, 2390–2401. doi:10.1093/eurheartj/ehv233

Ho, S. and Sanchez-Quintana, D. (2009). The importance of atrial structure and fibers. *Clinical Anatomy: The Official Journal of the American Association of Clinical Anatomists and the British Association of Clinical Anatomists* 22, 52–63

Ho, S. Y., Anderson, R. H., and Sánchez-Quintana, D. (2002). Atrial structure and fibres: morphologic bases of atrial conduction. *Cardiovascular Research* 54, 325–336. doi:10.1016/S0008-6363(02)00226-2

Krueger, M. W., Schmidt, V., Tobón, C., Weber, F. M., Lorenz, C., Keller, D. U. J., et al. (2011). Modeling atrial fiber orientation in patient-specific geometries: A semi-automatic rule-based approach. In *Functional Imaging and Modeling of the Heart*, eds. D. N. Metaxas and L. Axel (Berlin, Heidelberg: Springer Berlin Heidelberg), 223–232

Merhof, D., Sonntag, M., Enders, F., Hastreiter, P., Fahlbusch, R., Nimsky, C., et al. (2005). Visualization of diffusion tensor data using evenly spaced streamlines. *Vision, Modeling and Visualization* , 257–264

Mori, S. et al. (1999). Three-dimensional tracking of axonal projections in the brain by magnetic resonance imaging. *Annals of Neurology* 45, 265–269. doi:10.1002/1531-8249(199902)45:2⟨265::AID-ANA21⟩3.0.CO;2-3

Sawilowsky, S. S. (2009). New effect size rules of thumb. *Journal of Modern Applied Statistical Methods* 8, 26

Spach, M., Dolber, P., and Heidlage, J. (1988). Influence of the passive anisotropic properties on directional differences in propagation following modification of the sodium conductance in human atrial muscle. a model of reentry based on anisotropic discontinuous propagation. *Circulation research* 62, 811–832

Spach, M. S. and Dolber, P. C. (1986). Relating extracellular potentials and their derivatives to anisotropic propagation at a microscopic level in human cardiac muscle. evidence for electrical uncoupling of side-to-side fiber connections with increasing age. *Circulation research* 58, 356–371

Sun, Y., Bi, Q., Wang, X., Hu, X., Li, H., Li, X., et al. (2019). Prediction of conversion from amnestic mild cognitive impairment to alzheimer's disease based on the brain structural connectome. *Frontiers in Neurology* 9, 1178

Thomas, B. (2020). *An imaging pipeline for extracting atrial tissue architecture*. Ph.D. thesis, University of Auckland, Auckland, New Zealand

Wang, Y., Xiong, Z., Nalar, A., Hansen, B. J., Kharche, S., Seemann, G., et al. (2019). A robust computational framework for estimating 3D Bi-Atrial chamber wall thickness. *Computers in Biology and Medicine* 114, 103444. doi:10.1016/j.compbiomed.2019.103444

Wilcox, R. (2019). A robust nonparametric measure of effect size based on an analog of cohen's d, plus inferences about the median of the typical difference. *Journal of Modern Applied Statistical Methods* 17, 1

Zhao, J., Butters, T. D., Zhang, H., Pullan, A. J., LeGrice, I. J., Sands, G. B., et al. (2012a). An image-based model of atrial muscular architecture: Effects of structural anisotropy on electrical activation. *Circ. Arrhythm. Electrophysiol.* 5, 361–370

Zhao, J., Hansen, B. J., Wang, Y., Csepe, T. A., Sul, L. V., Tang, A., et al. (2017). Three-dimensional integrated functional, structural, and computational mapping to define the structural "fingerprints" of heart-specific atrial fibrillation drivers in human heart ex vivo. *Journal of the American Heart Association* 6, e005922

Zhao, J., Krueger, M. W., Seemann, G., Meng, S., Zhang, H., Dössel, O., et al. (2012b). Myofiber orientation and electrical activation in human and sheep atrial models. In *2012 Annual International Conference of the IEEE Engineering in Medicine and Biology Society*. 6365–6368
